# IgA transcytosis and antigen recognition govern ovarian cancer immunity

**DOI:** 10.1038/s41586-020-03144-0

**Published:** 2021-02-03

**Authors:** Subir Biswas, Gunjan Mandal, Kyle K. Payne, Carmen M. Anadon, Chandler D. Gatenbee, Ricardo A. Chaurio, Tara Lee Costich, Carlos Moran, Carly M. Harro, Kristen E. Rigolizzo, Jessica A. Mine, Jimena Trillo-Tinoco, Naoko Sasamoto, Kathryn L. Terry, Douglas Marchion, Andrea Buras, Robert M. Wenham, Xiaoqing Yu, Mary K. Townsend, Shelley S. Tworoger, Paulo C. Rodriguez, Alexander R. Anderson, Jose R. Conejo-Garcia

**Affiliations:** 1 Department of Immunology, H. Lee Moffitt Cancer Center and Research Institute, Tampa, FL USA; 2 Department of Mathematical Oncology, H. Lee Moffitt Cancer Center and Research Institute, Tampa, FL USA; 3 Department of Pathology, H. Lee Moffitt Cancer Center and Research Institute, Tampa, FL USA; 4 Obstetrics and Gynecology Epidemiology Center, Brigham and Women’s Hospital and Harvard Medical School, Boston, MA USA; 5 Department of Gynecology Oncology, H. Lee Moffitt Cancer Center and Research Institute, Tampa, FL USA; 6 Department of Biostatistics and Bioinformatics, H. Lee Moffitt Cancer Center and Research Institute, Tampa, FL USA; 7 Department of Cancer Epidemiology, H. Lee Moffitt Cancer Center and Research Institute, Tampa, FL USA; 8 Department of Epidemiology, Harvard T.H. Chan School of Public Health, Boston, MA USA

**Keywords:** Ovarian cancer, Tumour immunology

## Abstract

Most ovarian cancers are infiltrated by prognostically relevant activated T cells^1–3^, yet exhibit low response rates to immune checkpoint inhibitors^4^. Memory B cell and plasma cell infiltrates have previously been associated with better outcomes in ovarian cancer^5,6^, but the nature and functional relevance of these responses are controversial. Here, using 3 independent cohorts that in total comprise 534 patients with high-grade serous ovarian cancer, we show that robust, protective humoral responses are dominated by the production of polyclonal IgA, which binds to polymeric IgA receptors that are universally expressed on ovarian cancer cells. Notably, tumour B-cell-derived IgA redirects myeloid cells against extracellular oncogenic drivers, which causes tumour cell death. In addition, IgA transcytosis through malignant epithelial cells elicits transcriptional changes that antagonize the RAS pathway and sensitize tumour cells to cytolytic killing by T cells, which also contributes to hindering malignant progression. Thus, tumour-antigen-specific and -antigen-independent IgA responses antagonize the growth of ovarian cancer by governing coordinated tumour cell, T cell and B cell responses. These findings provide a platform for identifying targets that are spontaneously recognized by intratumoural B-cell-derived antibodies, and suggest that immunotherapies that augment B cell responses may be more effective than approaches that focus on T cells, particularly for malignancies that are resistant to checkpoint inhibitors.

## Abstract (web summary)

In patients with high-grade serous ovarian cancer, robust and protective humoral responses are dominated by B-cell-derived polyclonal IgA that binds to polymeric IgA receptors that are universally expressed on ovarian cancer cells.

## Main

Ovarian cancer is an immunogenic disease in which the pre-established immunoreactive landscape determines the outcome of the patient^1–3,7^. However, as monotherapies such as immune checkpoint inhibitors that augment T cell activity have only very modest response rates in patients with advanced disease^4^. Recent studies have suggested that plasma cell and memory B cell infiltrates, including those in tertiary lymphoid structures^8^, are associated with the cytolytic activity of T cells at ovarian cancer beds, resulting in improved survival of patients^5,6^. Although these studies suggest that humoral responses may potentiate T cell immune surveillance, the roles of different antibody isotypes in malignant progression are controversial.

**Extended Data Fig. 1:**
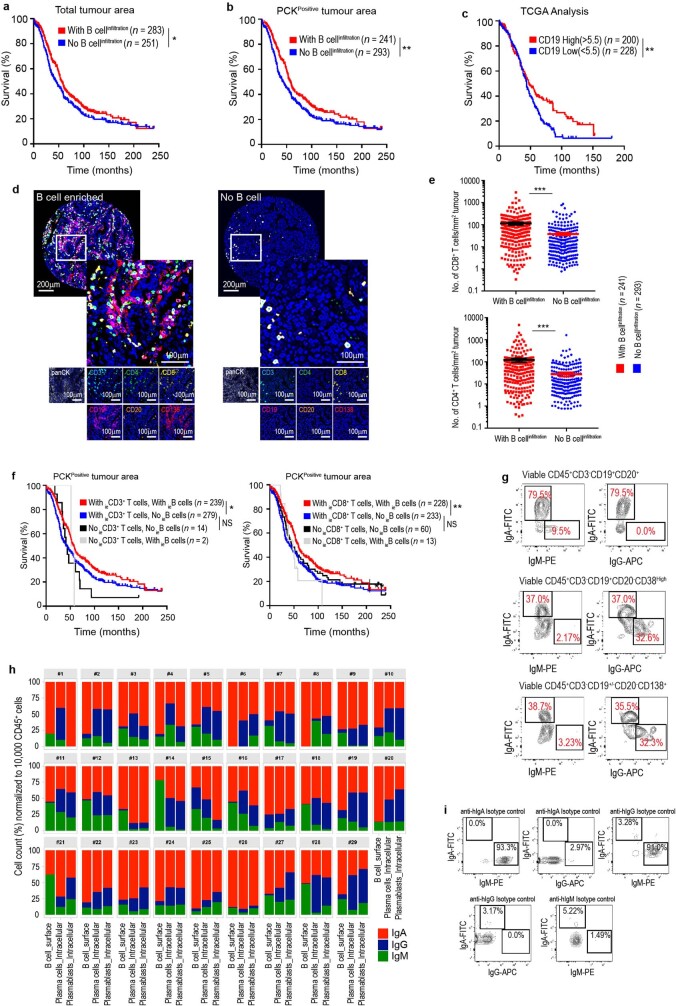
Isotype-switched humoral responses are associated with better outcome and denser T cell infiltration in HGSOC. **a**, **b**, Overall survival associated with the presence of CD19^+^ B cells within the total tumour area (*P* = 0.04) (**a**) or specifically in the PCK^+^ epithelial tumour islets (*P* = 0.0085) (**b**), in HGSOC as assessed by multiplex immunohistochemistry of TMAs corresponding to 534 patients with HGSOC combined from the NECC (*n* = 180), NHS (*n* = 261) and MCC (*n* = 93). B cell infiltration is defined as the presence of CD19^+^ cells on any of the duplicate sections analysed for each tumour. **c**, Survival outcome associated with the expression of CD19 in 428 annotated HGSOCs in TCGA datasets (*P* = 0.0091). *P ≤ 0.05, **P ≤ 0.01, two-sided log-rank (Mantel–Cox) test. **d**, Representative staining of the association between the accumulations of T and B cells at tumour beds (*n* = 534). Scale bar, 200 μm or 100 μm, as indicated. **e**, Accumulation of CD8^+^ (top) and CD4^+^ (bottom) T cells in the PCK^+^ tumour islets are associated with the presence of B cells. CD8: with versus without B cell infiltration, *P* < 0.0001; CD4: with versus without B cell infiltration, *P* < 0.0001. Data are mean ± s.e.m. ***P ≤ 0.001, unpaired two-tailed *t*-test. **f**, Overall survival associated with the presence of intra-epithelial CD19^+^ B cells and total intra-epithelial CD3^+^ T cells (left) or intra-epithelial CD3^+^CD8^+^ T cells (right) within ovarian carcinomas, as assessed by multiplex immunohistochemistry of TMAs corresponding to 534 patients with HGSOC combined from the NECC (*n* = 180), NHS (*n* = 261) and MCC (*n* = 93). With intra-epithelial CD3 T cells and with- intra-epithelial B cells versus with intra-epithelial CD3 T cells and no intra-epithelial B cells, *P* = 0.0123; With intra-epithelial CD3 T cells and no intra-epithelial B cells versus no intra-epithelial CD3 T cells and no intra-epithelial B cells, *P* = 0.0572; with intra-epithelial CD8 T cells and with intra-epithelial B cells versus with intra-epithelial CD8 T cells and no intra-epithelial B cells, *P* = 0.0035; with intra-epithelial CD8 T cells and no intra-epithelial B cells versus no intra-epithelial CD8 T cells and no intra-epithelial B cells, *P* = 0.4113. B cell and T cell infiltration are defined as the presence of CD19^+^ or CD3^+^ cells on any of the duplicate sections analysed for each tumour. *P ≤ 0.05, **P ≤ 0.01, NS, not significant, two-sided log-rank (Mantel–Cox) test. **g**, Representative FACS analysis of immunoglobulin isotypes on the surface of B cells infiltrating freshly dissociated human HGSOCs (*n* = 29). **h**, Bar graphs representing tumour-wise (*n* = 29) FACS analysis comparison of percentages of each Ig^+^ cells among total Ig^+^ B cells (CD45^+^CD3^−^CD19^+^CD20^+^), plasma cells (intracellular in CD45^+^CD3^−^CD19^+^CD20^−^CD138^+^ and CD45^+^CD3^−^CD19^−^CD20^−^CD138^+^) and plasmablasts (intracellular in CD45^+^CD3^−^CD19^+^CD20^-^CD38^high^), normalized to 10,000 viable CD45^+^ cells. **i**, FACS dot plots from analysis of IgA, IgG and IgM antibodies, and respective isotype controls, in human peripheral blood mononuclear cells, to evaluate the fidelity of the antibodies used. Source data

To characterize the role of B cells in ovarian cancer, we first stained a panel of 534 annotated high-grade serous ovarian cancers (HGSOCs) from 3 independent cohorts with T and B cell markers. As expected, CD19^+^ B cell infiltrates were associated with improved overall survival (Extended Data Fig. 1a, b), similar to data from The Cancer Genome Atlas (TCGA) (Extended Data Fig. 1c), and positively correlated with T cell infiltrates (Extended Data Fig. 1d, e). In addition, intra-epithelial T cells only predict improved survival^2^ when B cells co-infiltrate tumour islets (Extended Data Fig. 1f).

**Fig. 1:**
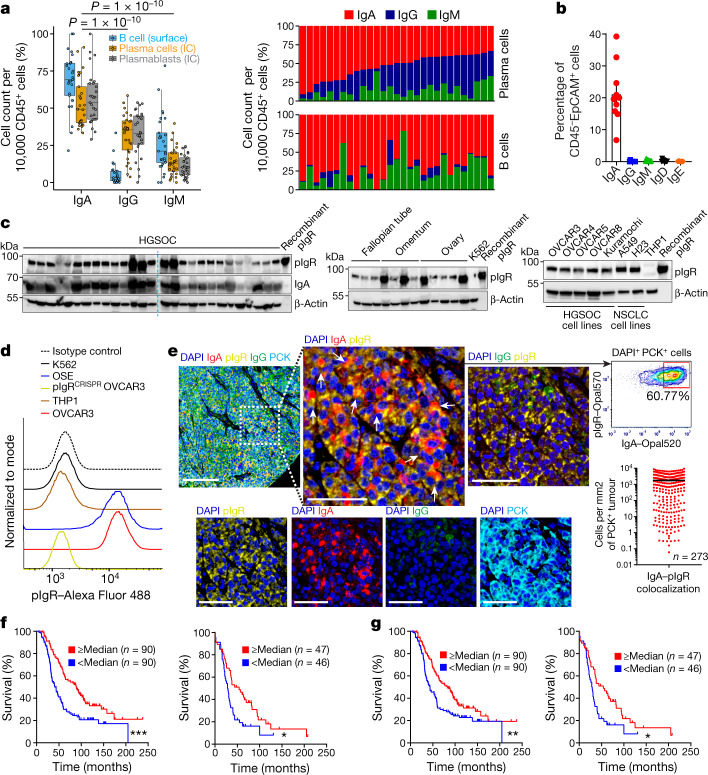
IgA–pIgR colocalization is associated with protective immunity in human ovarian cancer. **a**, Left, percentage of FACS cell counts of IgA^+^, IgG^+^ or IgM^+^ cells among Ig^+^ B cells or plasmablasts or plasma cells, normalized to 10,000 viable CD45^+^ cells. B cells, CD45^+^CD3^−^CD19^+^CD20^+^ cells; plasmablasts, CD45^+^CD3^−^CD19^+^CD20^−^CD38^high^ cells; plasma cells, CD45^+^CD3^−^CD19^+^CD20^−^CD138^+^ and CD45^+^CD3^−^CD19^−^CD20^−^CD138^+^ cells. Each dot represents one tumour (*n* = 29). Details of box plots can be found in Methods. *P* values were obtained by a two-way analysis of variance (ANOVA) followed by Dunnett’s test for multiple comparisons. Supplementary Table 1 provides further details on statistics. Right, bar graphs representing the percentage of each isotype produced by plasma cells (top) or B cells (bottom) in the same tumours, normalized to 10,000 viable CD45^+^ cells. IC, intracellular. **b**, IgA-coated CD45^−^EpCAM^+^ tumour epithelial cells (mean ± s.e.m., *n* = 10) in dissociated HGSOC. **c**, Expression of pIgR protein in independent HGSOC (*n* = 27); tumour-free Fallopian tube (*n* = 3), ovary (*n* = 5) and omental (*n* = 4) samples; ovarian tumour cell lines; and K562 leukaemia cells and THP1 monocyte cells (negative controls). Positive control, recombinant human pIgR. Western blots were repeated twice. NSCLC, non-small-cell lung cancer. **d**, Histograms showing FACS analysis of pIgR, in ovarian surface epithelial (OSE), K562, THP1, wild-type or *PIGR*-ablated (pIgR^CRISPR^) OVCAR3 cells. **e**, Left, representative (*n* = 273) combined staining of IgA, pIgR, IgG, PCK and DAPI. Instances with IgA–pIgR colocalization are indicated with arrows. Scale bar, 50 μm (top left), 20 μm (all other panels). Top right, representative (*n* = 137, IgA–pIgR colocalization ≥ median) dot plot showing IgA–pIgR colocalized signal among DAPI^+^PCK^+^ cells. Bottom right, scattered graph showing number of IgA–pIgR colocalized cells (averaged from duplicated cores) per mm^2^ of PCK^+^ (mean ± s.e.m., *n* = 273). **f**, Increased numbers of cells with IgA–pIgR colocalization per PCK^+^ tumour islet area (averaged from duplicated cores) are associated with improved outcome (threshold, median; *P* = 0.0116, H. Lee Moffett Cancer Centre cohort (MCC) (right); *P* = 0.0002, New England Case–Control study cohort (NECC) (left)). **g**, Density of IgA-coated cells (averaged from duplicated cores) in tumour islets (cells per mm^2^ PCK^+^ area) is associated with improved outcome (*P* = 0.0110 for MCC (right) and *P* = 0.0054 for NECC (left) cohorts). *P ≤ 0.05, **P ≤ 0.01, ***P ≤ 0.001, two-sided log-rank (Mantel–Cox) test. Source data

**Extended Data Fig. 2:**
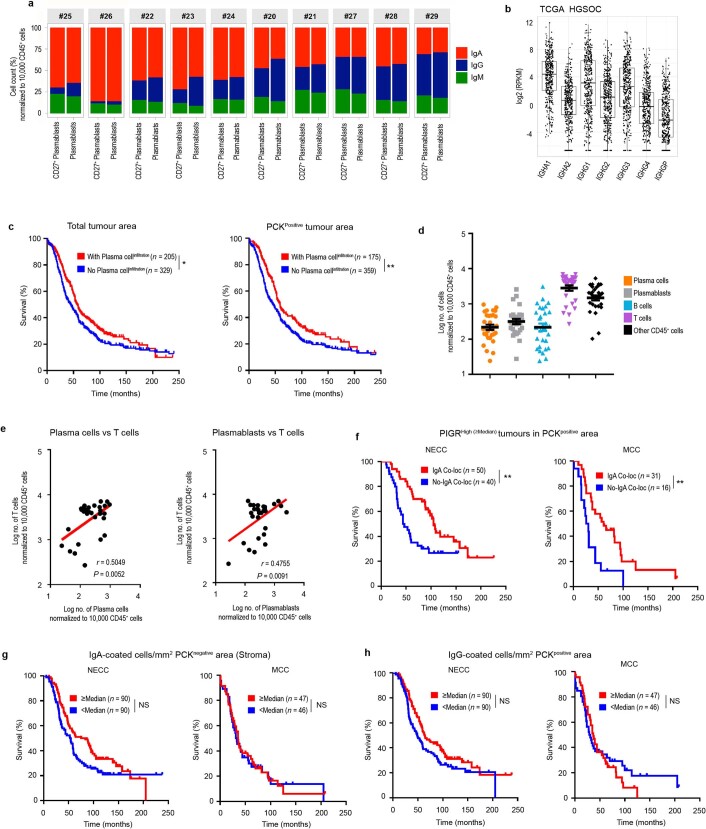
IgA coating of tumour cells is associated with a better outcome in HGSOC. **a**, Bar graphs representing tumour-wise FACS analysis (*n* = 10) comparison of percentages of each Ig^+^ cells among total Ig^+^ plasmablasts (intracellular in CD45^+^CD3^−^CD19^+^CD20^−^CD38^high^) and CD27^+^ plasmablasts (intracellular in CD45^+^CD3^−^CD19^+^CD20^−^CD38^high^CD27^+^), normalized to 10,000 viable CD45^+^ cells. **b**, Relative abundances of IgH chains on the basis of TCGA transcriptional analyses (*n* = 430) for each immunoglobulin heavy chain gene. Abundances are shown in log_2_-transformed reads per kilobase of transcripts (RPKM) values, which corrects for both gene length and sequencing depth. Details of box plots are in Methods. **c**, Overall survival associated with the presence of CD19^+^CD138^+^ plasma cells within the total tumour area (*P* = 0.0285) (left) or specifically in the PCK^+^ epithelial tumour islets (*P* = 0.0053) (right), in HGSOC as assessed by multiplex immunohistochemistry of TMAs corresponding to 534 patients with HGSOC combined from the NECC (*n* = 180), NHS (*n* = 261) and MCC (*n* = 93). Plasma cell infiltration is defined as the presence of CD19^+^CD138^+^ cells on any of the duplicate sections analysed for each tumour. *P ≤ 0.05, **P ≤ 0.01, two-sided log-rank (Mantel–Cox) test. **d**, FACS analysis showing number (log-transformed) of plasma cells (CD45^+^CD3^−^CD19^+^CD20^−^CD138^+^ and CD45^+^CD3^−^CD19^−^CD20^−^CD138^+^), plasmablasts (CD45^+^CD3^−^CD19^+^CD20^−^CD38^high^), B cells (CD45^+^CD3^−^CD19^+^CD20^+^), T cells (CD45^+^CD3^+^) and other leukocytes (CD45^+^CD3^−^CD19^−^CD20^−^CD138^−^) in HGSOC (*n* = 29). The data are normalized to 10,000 viable CD45^+^ leukocytes. Data are mean ± s.e.m. **e**, Graphs showing correlations between log count of T cells and plasma cells (left) (Pearson correlation coefficient (*r*) = 0.5049; two-sided nominal *P* = 0.0052); and between log count of T cells and plasmablasts (right) (Pearson correlation coefficient (*r*) = 0.4755; two-sided nominal *P* = 0.0091). All three cell types represent absolute counts normalized to 10,000 CD45^+^ leukocytes. **f**, Colocalization of IgA with pIgR^+^ cells (IgA–pIgR co-localization ≥ median) in the PCK^+^ tumour islets is associated with an improved outcome in HGSOC, compared to only pIgR^high^ samples (≥median) without IgA colocalization (colocalization < median), in MCC (*P* = 0.0060) and NECC (*P* = 0.0044) cohorts. **P ≤ 0.01, two-sided log-rank (Mantel–Cox) test. **g**, Higher number of IgA-coated cells in the PCK^−^ stromal area (average from duplicated cores) is not associated with an improved outcome in HGSOC, analysed using median IgA-coating threshold in MCC (*P* = 0.8954) and NECC (*P* = 0.0537) cohorts. NS, not significant; two-sided log-rank (Mantel–Cox) test, no multiple comparison adjustment. **h**, A higher number of IgG-coated cells in the PCK^+^ tumour islets (average from duplicated cores) is not associated with an improved outcome in HGSOC, analysed using median IgG-coating threshold in MCC (*P* = 0.6350) and NECC (*P* = 0.0731) cohorts. NS, not significant; two-sided log-rank (Mantel–Cox) test. Source data

To characterize the isotypes produced by these B cells, we analysed viable single-cell suspensions from 29 freshly dissociated HGSOCs. Intracellular staining of plasma cells and CD19^+^CD20^−^CD38^high^CD27^+^ cells (defined as plasmablasts) revealed the dominant production of class-switched IgA, followed by IgG; this is consistent with mRNA expression data from TCGA (Fig. 1a, Extended Data Figs. 1g–i, 2a, b) and with surface staining of B cells (Fig. 1a, Extended Data Fig. 1g–i).

As previously reported^6^, CD138^+^ plasma cell infiltrates were associated with improved survival in patients with HGSOC (Extended Data Fig. 2c), were identified in 80% of dissociated tumours in >1% of total leukocytes and also correlated with intratumoural T cells (Extended Data Fig. 2d, e).

**Extended Data Fig. 3:**
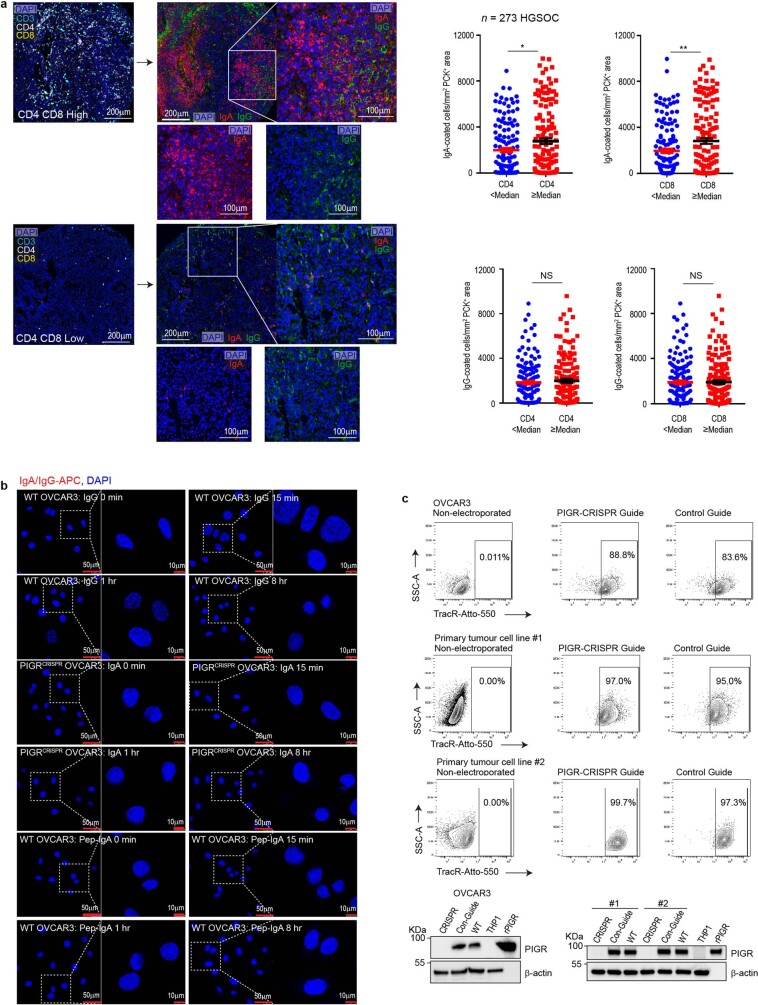
IgA internalization by HGSOC cells is associated with stronger T cell responses, and is dependent on pIgR–Fc interactions. **a**, Representative (*n* = 273) IgA and IgG staining in tumours with a high or low density of CD4^+^ and CD8^+^ T cells. Scale bar, 200 μm or 100 μm, as indicated. CD4^+^ and CD8^+^ T cell accumulation (≥median) is associated with the density of IgA-coated tumour (PCK^+^) cells (IgA: CD4 < median versus CD4 ≥ median, *P* = 0.0209; IgA: CD8 < median versus CD8 ≥ median, *P* = 0.0087); but not associated with IgG-coated tumour (PCK^+^) cells (IgG: CD4 < median versus CD4 ≥ median, *P* = 0.4451; IgG: CD8 < median versus CD8 ≥ median, *P* = 0.8304). Data are mean ± s.e.m. *P ≤ 0.05, **P ≤ 0.01, NS, not significant; unpaired two-tailed *t*-test. **b**, Representative confocal microscopy images of fluorescently (APC) labelled whole or pepsinized irrelevant IgA- or IgG-binding and internalization in pIgR^+^ (WT) or *PIGR*-ablated (PIGR^CRISPR^) OVCAR3 cells after 0 min, 15 min, 1 h or 8 h of incubation. Experiments were repeated three times. Scale bar, 50 μm or 10 μm, as indicated. **c**, FACS dot plots showing electroporation efficiency in pIgR-CRSIPR-guide (centre) or control-guide electroporated cells (right), compared to nonelectroporated OVCAR3 or primary HGSOC cells (*n* = 2) (left). Experiments were repeated three times. Western blots confirmed *PIGR* ablation in OVCAR3 and primary HGSOC cells (*n* = 2, repeated twice). THP1 cells were used as a negative control and recombinant pIgR (rPIGR) was used as a positive control. WT, wild type (non-electroporated cells). Source data

CD45^−^EpCAM^+^ tumour cells were also coated by IgA in all the dissociated HGSOCs we evaluated (Fig. 1b). Accordingly, we found universal expression of the polymeric immunoglobulin receptor (pIgR) in HGSOC, as well as in tumour-free fallopian tube, ovarian and omental tissue (but not in THP1 monocytes or in K562 leukaemia cells) (Fig. 1c). Expression of cell-surface pIgR was confirmed by flow cytometry in cancer and ovarian surface cell lines (Fig. 1d). Consequently, IgA and pIgR colocalized at tumour beds within cytokeratin (PCK)-positive tumour islets in 273 HGSOCs we analysed (Fig. 1e), and IgA–pIgR colocalization—but not pIgR-overexpression alone or stromal IgA—is associated with improved survival (Fig. 1f, Extended Data Fig. 2f, g). Importantly, the coating of tumour cells by IgA—but not IgG—is associated with improved survival (Fig. 1g, Extended Data Fig. 2h), and with increased intra-epithelial CD8^+^ and CD4^+^ T cells (Extended Data Fig. 3a).

**Fig. 2:**
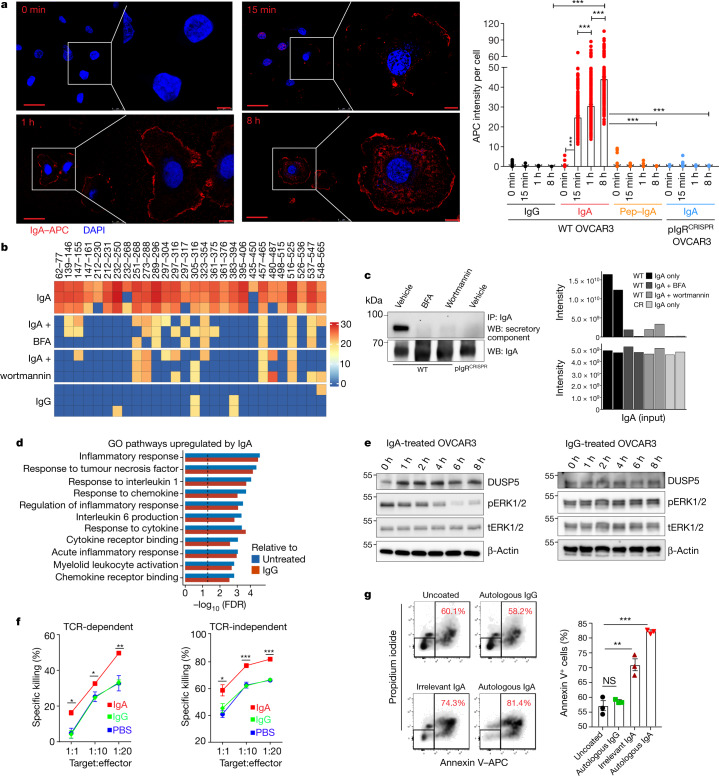
Transcytosis of IgA through pIgR^+^ ovarian cancer cells impairs tumour growth and augments cytotoxic killing mediated by T cells. **a**, Left, images of APC-labelled IgA binding and internalization in pIgR^+^ OVCAR3 cells (repeated three times). Scale bar, 50 μm (main panels), 10 μm (magnified regions). Right, comparison of antibody internalization signal (mean ± s.e.m.) in different treatment conditions and at different temporal points. Each dot represents quantification from one cell. ***P ≤ 0.001, unpaired two-tailed *t*-test. Supplementary Table 2 provides details of statistics. **b**, OVCAR3 cells were incubated with control IgA or IgG for 8 h in the presence of wortmannin, brefeldin A (BFA) or vehicle, and supernatants were subjected to liquid chromatography with tandem mass spectrometry (LC–MS/MS). Heat map of all peptides of the extracellular domain of pIgR (*n* = 3); scale represents log_2_-transformed intensities of pIgR peptide fragments detected in LC–MS/MS. **c**, Left, co-immunoprecipitates of supernatants from IgA-treated pIgR^+^ or *PIGR*-ablated OVCAR3 cells (with and without brefeldin A or wortmannin) blotted for the secretory component of pIgR and IgA (input control). Right, LC–MS/MS analysis of the co-immunoprecipitates showing intensities (log_2_-transformed) of the secretory component of pIgR and IgA (*n* = 2). WT, wild type; CR, *PIGR*-ablated. **d**, Pre-ranked gene-set enrichment analysis (GSEA), showing the top upregulated gene sets in OVCAR3 cells treated with irrelevant IgA compared to IgG or untreated cells (*n* = 3), Kolmogorov–Smirnov test. GO, Gene Ontology. **e**, Progressive increase in DUSP5 and concomitant reduction in phospho-ERK1 and phospho-ERK2 (pERK1/2) after IgA treatment (left) of OVCAR3 cells, but not IgG treatment (right). Experiments were repeated three times. tERK1/2, total ERK1 and total ERK2. **f**, Left, dose-dependent cytotoxic killing of NY-ESO-1-transduced OVCAR3 cells (*n* = 3) by NY-ESO-1–TCR-transduced T cells is augmented by co-incubation with IgA, compared to IgG or PBS. Right, IgA treatment also augmented the anti-tumour activity of FSH-targeted chimeric receptor T cells. Mean ± s.e.m. *P ≤ 0.05, **P ≤ 0.01, ***P ≤ 0.001, ordinary one-way ANOVA. Supplementary Table 2 provides details of statistics. **g**, Cytotoxic killing of primary CD45^−^EpCAM^+^ tumour cells (*n* = 3) by autologous tumour-infiltrating T cells (1:1 ratio) is augmented by co-incubation with autologous (*P* < 0.0001) or irrelevant IgA (*P* = 0.0031), but not autologous IgG (*P* = 0.1951). Mean ± s.e.m. **P ≤ 0.01, ***P ≤ 0.001, NS, not significant; unpaired two-tailed *t*-test. Source data

**Extended Data Fig. 4:**
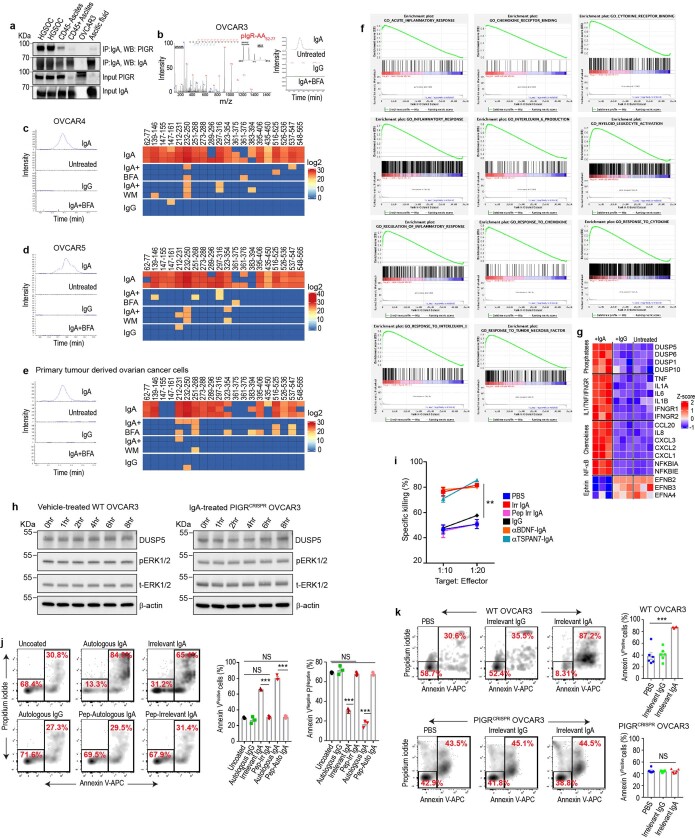
IgA transcytosis through HGSOC cells has substantial anti-tumour effects and sensitizes tumour cells to cytolytic killing by T cells. **a**, Immunoblots showing pIgR co-immunoprecipitation with IgA, using nondenaturing lysates from two HGSOCs, CD45^+^ and CD45^−^ cells isolated from human ovarian cancer ascites, ascitic fluid and OVCAR3 cells (negative control). Inputs were immunoblotted using 1% of the amount of lysate used for the co-immunoprecipitation. The experiments were repeated three times. **b**, OVCAR3 cells were incubated with 0.5 μg ml^−1^ of control IgA or IgG for 8 h in serum-free medium in the presence of wortmannin (1 μM), brefeldin A (1 μg ml^−1^) or vehicle, and supernatants were subjected to mass spectrometry. The amino acids 62–77 fragment of pIgR was found only after incubation with IgA (repeated three times). **c–e**, OVCAR4 (**c**), OVCAR5 (**d**) and primary HGSOC (**e**) tumour cells were incubated with 0.5 μg ml^−1^ of irrelevant IgA or IgG for 8 h in serum-free medium in the presence of wortmannin (1 μM), brefeldin A (1 μg ml^−1^) or vehicle, and supernatants were then subjected to mass spectrometry (left). Right, heat map of all peptides of the extracellular domain of pIgR (*n* = 3). BFA, brefeldin A. WM, wortmannin. **f**, **g**, GSEA enrichment plots (**h**) and heat maps (**g**) using normalized gene expression from RNA-seq analysis from OVCAR3 cells with irrelevant IgA (0.5 μg ml^−1^), IgG (0.5 μg ml^−1^) or no treatment for 24 h (*n* = 3 experiments). **h**, No change in the protein levels of DUSP5, total ERK1/2 or phospho-ERK1/2 after vehicle (PBS) treatment of pIgR^+^ (WT) OVACR3 cells (left) or after IgA treatment of *PIGR*-ablated OVACR3 cells (right), incubated up to 8 h. The experiments were repeated three times. **i**, Dose-dependent cytotoxic killing of OVCAR3 cells by FSH-targeted chimeric receptor T cells is augmented by co-incubation with 0.5 μg ml^−1^ of irrelevant IgA, anti-TSPAN7–IgA or anti-BDNF–IgA compared to IgG, pepsinized irrelevant IgA or PBS. *n* = 3 per group. **P ≤ 0.01, unpaired two-tailed *t*-test. **j**, Cytotoxic killing of autologous CD45^−^EpCAM^+^ tumour cells (with corresponding decrease of annexin V^−^propidium iodide (PI)^−^ viable cells) by autologous T cells (added at 1:1 ratio) is augmented by co-incubation with 0.5 μg ml^−1^ of autologous IgA or irrelevant IgA but not with autologous IgG, pepsinized autologous or irrelevant IgA, as compared to uncoated cells (*n* = 3). Annexin V^+^: irrelevant IgA versus pepsinized irrelevant IgA, *P* < 0.0001; autologous IgA versus pepsinized autologous IgA, *P* < 0.0001; uncoated versus pepsinized irrelevant IgA, *P* = 0.3769; uncoated versus pepsinized autologous IgA, *P* = 0.2208. Annexin V^−^PI^−^ cells: irrelevant IgA versus pepsinized irrelevant IgA, *P* < 0.0001; autologous IgA versus pepsinized autologous IgA, *P* < 0.0001; uncoated versus pepsinized irrelevant IgA, *P* = 0.3329; uncoated versus pepsinized autologous IgA, *P* = 0.1916. Data are mean ± s.e.m. *** *P* ≤ 0.001, NS, not significant; unpaired two-tailed *t*-test. **k**, Cytotoxic killing of pIgR^+^ OVCAR3 cells, but not pIgR^CRISPR^ OVCAR3 cells, by FSH-targeted chimeric receptor T cells (added at 1:1 ratio) is augmented by co-incubation with 0.5 μg ml^−1^ of irrelevant IgA compared to IgG-coated or uncoated cells (*n* = 6). Wild-type OVCAR3: PBS or irrelevant IgG versus irrelevant IgA, *P* < 0.0001. pIgR^CRISPR^ OVCAR3: PBS versus irrelevant IgA, *P* = 0.7728; irrelevant IgG versus irrelevant IgA, *P* = 0.9176. ***P ≤ 0.001, NS, not significant; unpaired two-tailed *t*-test. Source data

To determine whether IgA–pIgR interactions elicit transcytosis through tumour cells, we first incubated pIgR^+^ OVCAR3 ovarian cancer cells with fluorescently labelled non-antigen-specific IgA or IgG (Fig. 2a, Extended Data Fig. 3b). Confocal microscopy confirmed that IgA was selectively internalized and deposited on the cell surface within 8 h (Fig. 2a). Internalization was abrogated upon pepsin-mediated removal of the Fc or CRISPR-mediated ablation of *PIGR* (Fig. 2a, Extended Data Fig. 3b, c), and co-immunoprecipitation analyses of IgA and pIgR confirmed their physical interaction in human HGSOC (Extended Data Fig. 4a). In support of the notion that IgA indeed transcytoses through tumour cells, several peptides of the secretory component were detected in the supernatants of OVCAR3, OVCAR4, OVCAR5 or primary ovarian cancer cells incubated with IgA, but not when these cells were co-incubated with the transcytosis inhibitors wortmannin and brefeldin A^9,10^, or when cells were incubated with IgG (Fig. 2b, Extended Data Fig. 4b–e, Supplementary Data 1, 2). Finally, IgA co-immunoprecipitated with the secretory component in OVCAR3 supernatants, and this was again abolished by transcytosis inhibitors or *PIGR* ablation in tumour cells (Fig. 2c, Supplementary Data 3).

Notably, IgA transcytosis induced broad transcriptional changes in inflammatory pathways in tumour cells, including the upregulation of IFNγ receptors (Fig. 2d, Extended Data Fig. 4f, g) and downregulation of tumour-promoting ephrins^11^ (Extended Data Fig. 4g, Supplementary Data 4). In addition, several DUSP phosphatases—which are known to counteract phosphorylation events downstream of the RAS pathway^12^—were simultaneously increased upon incubation with non-antigen-specific IgA (but not IgG or vehicle, or in *PIGR*-ablated, IgA-treated cells), at both the mRNA (Extended Data Fig. 4g) and protein levels (Fig. 2e, Extended Data Fig. 4h). Finally, increases in DUSP5 were associated with impaired MEK–ERK signalling, as demonstrated by reduced levels of phospho-ERK1 and phospho-ERK2 (Fig. 2e).

**Extended Data Fig. 5:**
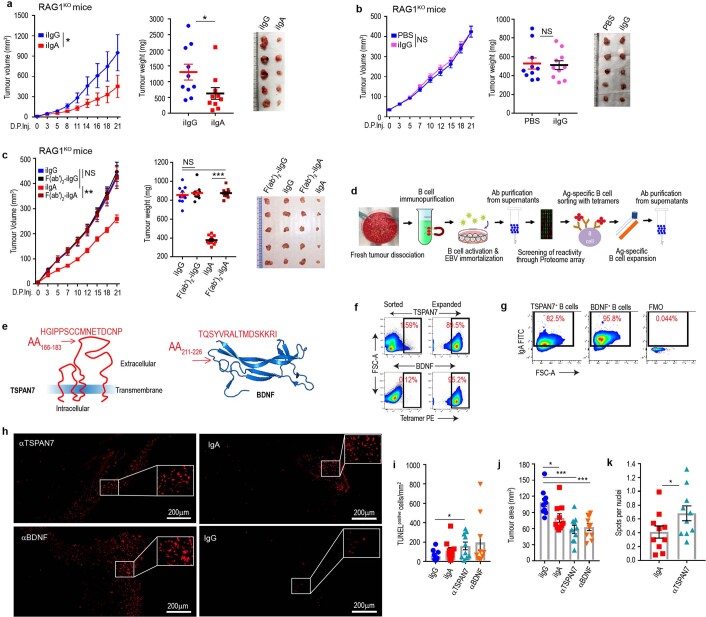
TSPAN7- and BDNF-specific antibodies produced at tumour beds delay the progression of established tumours. **a**, RAG1-deficient mice inoculated subcutaneously with 10^7^ OVCAR3 cells received 100 μg per 20g body weight of irrelevant (i)IgA or irrelevant (i)IgG peritumourally at days 7, 11, 15, 19 and 23 after tumour inoculation. Tumour growth curves (left), tumour weight (centre) and representative differences in tumour volume (right) are shown. Growth curve statistics: iIgG versus iIgA, *P* = 0.0130, paired two-tailed *t*-test. Tumour weight statistics, iIgG versus iIgA, *P* = 0.043, unpaired two-tailed *t*-test. Data are mean ± s.e.m. *P ≤ 0.05. **b**, Tumour growth curves (left), as well as tumour volume (right) and weight (centre) in OVCAR3-tumour-bearing RAG1-deficient mice receiving irrelevant IgG antibodies or vehicle (PBS). Growth curve statistics: iIgG versus PBS, *P* = 0.1840, paired two-tailed *t*-test. Tumour weight statistics: iIgG versus PBS, *P* = 0.8275, (unpaired two-tailed *t*-test). Data are mean ± s.e.m. NS, not significant. **c**, Tumour growth curves (left), as well as tumour volume (right) and weight (centre) in OVCAR3-tumour-bearing RAG1-deficient mice receiving full-length or pepsinized (Fc-removed) irrelevant IgG or irrelevant IgA antibodies. Curves and tumour weights were pooled from 2 independent experiments (10 mice per group in total). Growth curve statistics: iIgA versus F(ab′)_2_–iIgA, *P* = 0.0030; iIgA versus iIgG, *P* = 0.0578; iIgG versus F(ab′)_2_–iIgG, *P* = 0.0547, paired two-tailed *t*-test. Tumour weight statistics: iIgA versus F(ab′)_2_–iIgA, *P* < 0.0001; iIgG versus F(ab′)_2_–iIgG, *P* = 0.5585; iIgG versus F(ab′)_2_–iIgA, *P* = 0.5382, unpaired two-tailed *t*-test. Data are mean ± s.e.m. **P ≤ 0.01, *** *P* ≤ 0.001, NS, not significant. In **a**–**c**, growth curves and tumour weights were pooled from 2 independent experiments (*n* = 10 mice per group in total). **d**, Schematic of the optimized protocol for separating, immortalizing, characterizing and selecting tumour-reactive B cells from HGSOCs. **e**, Tetramers spanning the indicated loop in BDNF or the extracellular domain of TSPAN7 were used to sort reactive B cells immortalized from ten independent HGSOCs. **f**, The reactivity of expanded cells was confirmed using the same tetramers. **g**, IgA represents the majority of TSPAN7- or BDNF-reactive B cells sorted from HGSOCs. **h**, Representative TUNEL (Alexa Fluor 647) staining images in xenograft tumours developed in RAG1-knockout mice. Scale bar, 200 μm. **i**, Estimation of TUNEL^+^ cells normalized to tumour area. iIgG versus anti-TSPAN7, *P* = 0.0293. Data are mean ± s.e.m. *P ≤ 0.05, unpaired two-tailed *t*-test. **j**, Tumour area quantification. iIgG versus iIgA, *P* = 0.0146; iIgG versus anti-BDNF, *P* = 0.0002; iIgG versus anti-TSPAN7, *P* = 0.0002. Data are mean ± s.e.m. *P ≤ 0.05, ***P ≤ 0.001, unpaired two-tailed *t*-test. **k**, Quantification of irrelevant IgA and anti-TSPAN7–IgA antibody uptake in OVACR3 xenografts. *P* = 0.0343. Data are mean ± s.e.m. *P ≤ 0.05, unpaired two-tailed *t*-test. In **h**–**k**, *n* = 10 mice per group pooled from 2 independent experiments. Source data

To define the functional relevance of phenotypic changes induced by IgA transcytosis in ovarian cancer cells, we expressed the cancer testis antigen NY-ESO-1 in HLA-A2^+^FSHR^+^ OVCAR3 HGSOC cells, as well as an HLA A2-restricted T cell receptor (TCR) in human T cells that recognizes SLLMWITQC (which corresponds to amino acids 157–165 of NY-ESO-1)^13^. The dose-dependent cytotoxic activity of tumour-antigen-redirected T cells was enhanced upon incubation with irrelevant IgA, compared to control IgG or vehicle (Fig. 2f, left). These effects were independent of changes in MHC-I expression, as the cytotoxic activity of human T cells engineered to express an FSH-targeted chimeric receptor^14^—which recognizes FSHR in OVCAR3 cells independently of MHC-I—was enhanced to a similar extent (Fig. 2f, right). Comparable IgA-dependent sensitization of tumour cells to T-cell-mediated killing was identified using expanded tumour-infiltrating lymphocytes and autologous tumour cells from different patients (Fig. 2g), and a similar enhancement was observed using different tumour antigen-specific IgA (Extended Data Fig. 4i). Increased T cell cytotoxicity required interaction between the Fc domain of IgA and pIgR, because it was abolished using pepsinized antibodies or *PIGR*-ablated OVCAR3 cells (Extended Data Fig. 4j, k). Accordingly, treatment with non-antigen-specific IgA significantly delayed the growth of OVCAR3 tumours in RAG1-deficient tumour-bearing mice, compared to control IgG or pepsinized IgA (Extended Data Fig. 5a–c). Suppression of tumour growth was not due to any tumour-promoting effect of IgG^15^, as tumour-bearing mice treated with PBS or pepsin-treated (F(ab′)_2_) immunoglobulin fragments grew at the same rate as their control IgG-treated counterparts (Extended Data Fig. 5b, c).

**Fig. 3:**
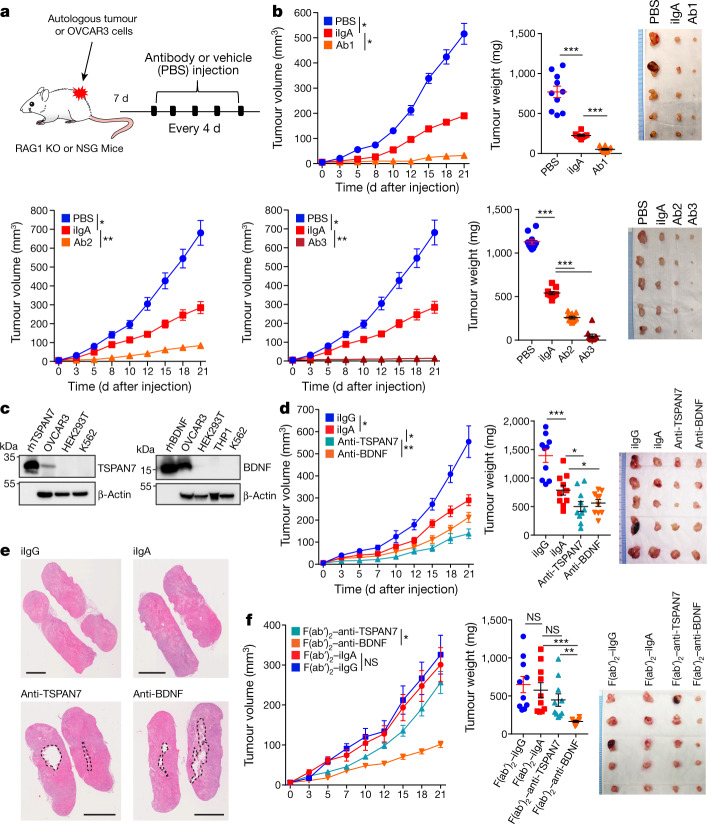
Tumour-antigen-specific IgA produced in the ovarian cancer microenvironment antagonizes ovarian cancer progression. **a**, Schematic of design of experiment shown in **b**, **d**, **f**. Antibody at 100 μg per 20 g body weight or equal volume of vehicle (PBS) was intratumourally injected. KO, knockout. **b**, Autologous tumour growth curves (left), weight (centre) and volume (right) in tumour-bearing RAG1-knockout mice receiving control (irrelevant IgA (iIgA)) or recombinant dimeric IgA antibodies (labelled Ab1, Ab2 or Ab3) produced with three different matching IgA sequences clonally expanded in two different HGSOC. Respective autologous HGSOC cells were used (tumour no. 1 for Ab1 and tumour no. 2 for Ab2 or Ab3). Supplementary Table 3 provides details of statistics. **c**, IgA purified from TSPAN7- and BDNF-reactive immortalized B cells recognizes the corresponding recombinant proteins in western blot analysis, along with endogenous TSPAN7 and BDNF expressed in OVCAR3 cells. HEK293T, THP1 and K562 cells were included as negative controls. Experiments were repeated three times. rhTSPAN7, recombinant human TSPAN7; rhBDNF, recombinant human BDNF. **d**, Tumour growth curves (left), weight (centre) and tumour volume (right) in tumour-bearing RAG1-knockout mice receiving control or tumour-derived antibodies. Supplementary Table 4 provides details of statistics. **e**, Representative images (*n* = 10 per group from 2 independent experiments) of central necrosis in tumours from mice receiving IgA from tumour-derived B cells. Scale bars, 4 mm. **f**, Antibodies used in **d** were digested with pepsin to remove their Fc domain and resulting F(ab′)_2_ fragments used to treat OVCAR3 tumour-bearing RAG1-knockout mice under identical conditions. Tumour growth curves (left), weight (centre) and tumour volume (right). Supplementary Table 5 provides details of statistics. In **b**, **d**, **f**, growth curves and tumour weights were pooled from 2 independent experiments (*n* = 10 mice per group in total). Data are mean ± s.e.m. *P ≤ 0.05, **P ≤ 0.01, ***P ≤ 0.001, NS, not significant; paired two-tailed *t*-test for growth curves or unpaired two-tailed *t*-test for tumour weights. Source data

To define the relative contribution of antigen recognition by tumour-derived IgA to delayed malignant progression, we produced recombinant dimeric IgA antibodies by cloning the matching VH and VL sequences of three clonally expanded, IgA-producing tumour-derived B cells, as determined by single-cell B cell receptor sequencing (Supplementary Data 5). All recombinant antibodies abrogated the progression of autologous cancer cells in vivo more effectively than did control IgA antibodies (Fig. 3a, b).

**Fig. 4:**
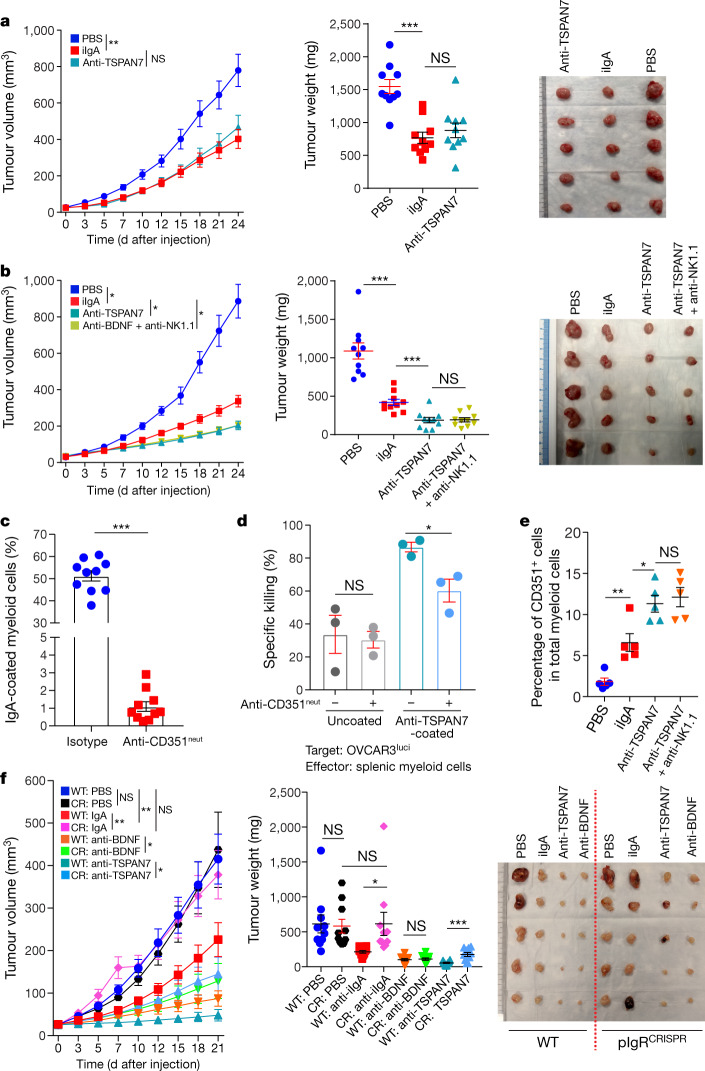
Antigen-specific IgA redirects Fcα/μR-positive myeloid cells against cell-surface antigen-positive tumour cells. **a**, Tumour growth curves (left), tumour weight (centre) and volume (right) in tumour-bearing NSG mice receiving control or tumour-derived antibodies. Supplementary Table 6 provides for details of statistics. **b**, Tumour growth curves (left), tumour weight (centre) and volume (right) in tumour-bearing RAG1-knockout mice receiving control or tumour-derived antibodies ± intraperitoneal injections of anti-NK1.1 or control antibodies. Supplementary Table 7 provides for details of statistics. In **a**, **b**, growth curves and tumour weights were pooled from 2 independent experiments (*n* = 10 mice per group in total). **c**, Binding of IgA antibodies to splenic CD11b^+^ cells from tumour-bearing RAG1-knockout mice (*n* = 10), after incubation with Fcα/μR (CD351)-neutralizing antibodies or isotype controls. *P* < 0.0001. **d**, Cytotoxic killing of OVCAR3 cells by splenic myeloid cells from tumour-bearing RAG1-knockout mice (1:1 ratio) is augmented by coating the tumour cells with anti-TSPAN7, and inhibited by neutralizing CD351 (anti-CD351^neut^) (*n* = 3). Supplementary Table 8 provides details of statistics. OVCAR3^Luci^, OVCAR3 cells transduced with luciferase-expressing vector. **e**, Increased accumulation of CD351^+^ myeloid cells in xenografts in RAG1-knockout mice treated with intratumoural anti-TSPAN7, compared to irrelevant IgA or vehicle, irrespectively of NK1.1 depletion (*n* = 5). Supplementary Table 8 provides for details of statistics. **f**, Tumour growth curves (left), tumour weight (centre) and volume (right) in wild-type pIgR^+^ (WT) or *PIGR*-ablated (pIgR^CRISPR^ (CR)) OVCAR3 tumour-bearing RAG1-knockout mice receiving control or tumour-derived antibodies. Two independent experiments were performed with similar results; tumour growth was represented from 1 experiment (*n* = 5 mice per group); tumour weights were pooled from 2 experiments (*n* = 10 mice per group in total). Supplementary Table 9 provides details of statistics. Data are mean ± s.e.m. *P ≤ 0.05, **P ≤ 0.01, ***P ≤ 0.001, NS, not significant; paired two-tailed *t*-test for growth curves or unpaired two-tailed *t*-test for tumour weights and other experiments. Source data

**Extended Data Fig. 6:**
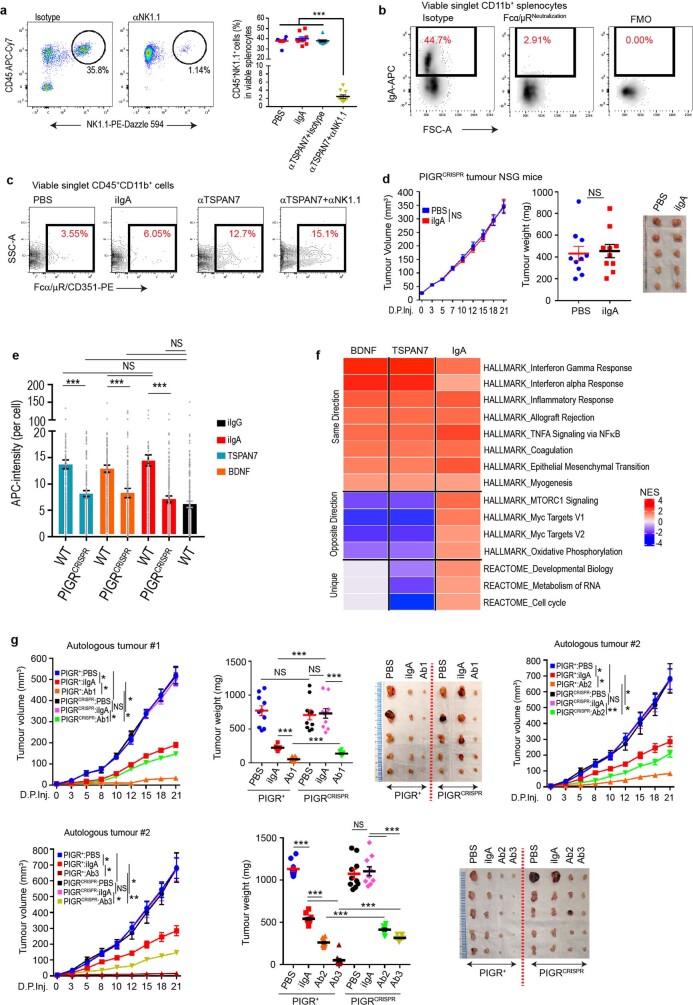
Tumour-derived IgA abrogates tumour growth through antigen-dependent redirection of Fcα/μR^+^ myeloid cells and antigen-independent, pIgR-mediated transcytosis. **a**, Dot plots showing FACS analysis of splenocytes for NK1.1 depletion in RAG1-KO mice (left). Scatter plot showing CD45^+^NK1.1^+^ cells percentages among viable splenocytes in respective treatment-group mice (*n* = 10 mice per group pooled from 2 independent experiments) (right). *P* < 0.0001, ordinary one-way ANOVA. Data are mean ± s.e.m. ***P ≤ 0.001. **b**, Representative FACS dot plots showing binding of IgA antibodies to splenic CD11b^+^ cells from tumour-bearing RAG1-deficient mice, after incubation with Fcα/μR (CD351)-neutralizing antibodies or isotype controls (*n* = 10). **c**, Representative FACS dot plots show CD351^+^ cells among viable CD45^+^CD11b^+^ cells in xenografts in RAG1-deficient mice treated with intratumoural anti-TSPAN7 (with or without NK1.1-depletion), compared to irrelevant IgA or vehicle (PBS) (*n* = 10 mice per group pooled from 2 independent experiments). **d**, Tumour growth curves (left), as well as tumour volume (right) and weight (centre) in pIgR^CRISPR^ OVCAR3-tumour-bearing NSG mice receiving irrelevant IgA antibodies or vehicle (PBS). Paired or unpaired two-tailed *t*-tests for tumour growth curves or tumour weight comparisons, respectively. Data are mean ± s.e.m. NS, not significant. **e**, Internalized intensity of antibodies (APC) were quantified and scattered bar graph showing comparison of antibody internalization in different treatment conditions, in which each dot represents quantification from one cell, pooled from three independent experiments. Supplementary Table 11 provides details of statistics. Data are mean ± s.e.m. ***P ≤ 0.001, NS, not significant; unpaired two-tailed *t*-test. **f**, Pathway analysis of RNA-seq from OVCAR3 cells treated with irrelevant IgA (0.5 μg ml^−1^), anti-TSPAN7–IgA (0.5 μg ml^−1^), anti-BDNF–IgA or no treatment for 24 h (*n* = 3). **g**, Autologous tumour growth curves, tumour weight and volume in wild-type pIgR^+^ (WT) or *PIGR*-ablated (pIgR^CRISPR^) autologous tumour-bearing RAG1-deficient mice receiving control or recombinant dimeric IgA antibodies (Ab1, Ab2 or Ab3) produced with three different matching IgA sequences clonally expanded in two different HGSOCs. Respective autologous HGSOC cells were used (tumour no. 1 for Ab1 and tumour no. 2 for Ab2 or Ab3). Data are mean ± s.e.m. *P ≤ 0.05, **P ≤ 0.01, ***P ≤ 0.001, NS, not significant. Supplementary Table 12 provides details of statistics. In **d**, **g**, curves and tumour weights were pooled from 2 independent experiments (*n* = 10 mice per group in total). Source data

To identify the specificities of antibodies that are spontaneously generated in ovarian cancer, we optimized a system for the isolation, activation, immortalization and characterization of B cells immunopurified from ten freshly dissociated HGSOCs, using human proteome arrays (Extended Data Fig. 5d). We found that IgA and IgG antibodies secreted by tumour-derived B cells recognized a broad range of tumour antigens, many of which have extracellular domains or represent secreted proteins (Supplementary Data 6). To define the functional relevance of extracellular antigen recognition, we focused on TSPAN7 (a tetraspanin that is overexpressed in human carcinomas^16^) and on BDNF (a secreted molecule that is associated with poor prognosis in HGSOC^17^) (Extended Data Fig. 5e). We tetramerized a battery of biotinylated 16–20-mer peptides contained in the extracellular domains of these molecules using fluorescent streptavidin, and sorted tetramer-reactive B cells by fluorescence-activated cell sorting (FACS) from our immortalized batches of intratumoural B cells, and cultured them separately (Extended Data Fig. 5f). These B cells predominately produced IgA (Extended Data Fig. 5g) that specifically recognized these targets expressed in HGSOC tumour cells, as well as recombinant TSPAN7 and BDNF (Fig. 3c). Notably, both TSPAN7- and BDNF-reactive IgA: (1) antagonized tumour growth in vivo more effectively than did irrelevant IgA (Fig. 3d); (2) induced areas of central necrosis and TUNEL^+^ cells (Fig. 3e, Extended Data Fig. 5h–j); and (3) was engulfed by tumour cells more effectively than was irrelevant IgA (Extended Data Fig. 5k). The anti-tumour effects of BDNF-specific antibodies were retained upon removal of the Fc domain (which suggests the neutralization of secreted BDNF), and pepsinized anti-TSPAN7 antibodies lost their anti-tumour activity, which is suggestive of antibody-dependent cellular cytotoxicity or phagocytosis (Fig. 3f). Accordingly, the superior activity of TSPAN7 antibodies (as compared to control IgA) disappeared in NOD-SCID-gamma (NSG) mice, which lack functional macrophages, dendritic cells and natural killer cells (Fig. 4a). Natural killer cell depletion in tumour-bearing RAG1-knockout mice (Extended Data Fig. 6a) had no effect on anti-tumour activity (Fig. 4b). In further support of antibody-dependent cellular phagocytosis, splenic myeloid cells from tumour-bearing (CD89-deficient) mice bound IgA through Fcα/μR (CD351) (Fig. 4c, Extended Data Fig. 6b), and killed OVCAR3 targets more effectively upon coating with TSPAN7-reactive IgA (Fig. 4d). Importantly, there were increases in CD351^+^ myeloid cells at tumour beds after treatment with TSPAN7 antibodies as compared to control IgA (Fig. 4e, Extended Data Fig. 6c). Therefore, polyclonal tumour antigen-specific IgA responses hinder malignant progression through at least two independent mechanisms.

To define the role of pIgR-mediated IgA transcytosis in anti-tumour activity, we challenged RAG1-deficient mice with *PIGR*-ablated OVCAR3 tumours. Notably, the protective effect of non-antigen-specific IgA disappeared in both RAG1-deficient and NSG mice (Fig. 4f, Extended Data Fig. 6d) and tumour-derived TSPAN7 and BDNF antibodies showed decreased anti-tumour effects, consistent with the capacity to transcytose through tumour cells (Extended Data Fig. 6e, f, Supplementary Data 7). In further support of the relevance of antigen recognition, significant anti-tumour effects were elicited by clonally expanded IgA, but not by control irrelevant IgA against *PIGR*-ablated autologous tumours (Extended Data Figs. 3c, 6g).

Collectively these data demonstrate that IgA produced in the ovarian cancer microenvironment contributes to thwarting malignant progression through both antigen recognition, and via non-specific transcytosis through pIgR^+^ tumour cells. These findings indicate that immunotherapies that boost both coordinated B and T cell responses against human ovarian cancer—an immunogenic disease that is currently resistant to checkpoint inhibitors—are likely to show increased therapeutic benefit. In support of this notion, a similar synergy has previously been suggested for other malignancies^18–21^. Furthermore, IgA-based immunotherapies could be more effective than conventional IgG-targeted interventions against ovarian cancer or other pIgR^+^ mucosal tumours.

## Methods

No statistical methods were used to predetermine sample size. The experiments were not randomized. Investigators were not blinded to allocation during experiments and outcome assessment, unless otherwise mentioned.

### Human samples

Human ovarian carcinoma tissues were procured under protocols approved by the Committee for the Protection of Human Subjects at Dartmouth–Hitchcock Medical Center (no. 17702); and under a protocol approved by the H. Lee Moffitt Cancer Center (MCC no. 19767 and MCC no. 18974). Part of the tumour tissues were either snap-frozen for protein extraction, or were freshly dissociated and cryopreserved. Viable cells in thawed samples were gated for FACS analysis using viability dyes (BioLegend). We have analysed tissue microarrays (TMAs) procured from the Moffitt Cancer Center Tissues Core, which include 93 HGSOC and some control tissues (MCC cohort) (approval MCC no. 50264). Tumour tissue from 261 HGSOCs from 2 prospective cohort studies (the Nurses’ Health Study (NHS) and NHSII^22^) and from 180 HGSOCs from a population-based case–control study (New England Case–Control Study (NECC cohort)) were used. In brief, the NHS enrolled 121,700 female registered nurses, aged 30–55, residing in 1 of 11 states of the USA in 1976. Similarly, the NHSII enrolled 116,429 female registered nurses aged 25–42 residing in 1 of 14 states of the USA in 1989. At baseline, women completed a mailed baseline questionnaire on their lifestyle and medical history. Questionnaires to collect updated information have since been mailed biennially. Incident epithelial ovarian cancer cases were identified from questionnaires, reports from family or linkage to the National Death Index, and confirmed by medical record review or cancer registry linkage. The study protocol was approved by the institutional review boards of the Brigham and Women’s Hospital and Harvard T. H. Chan School of Public Health, and those of participating registries as required. Return of the questionnaires is considered to imply informed consent.

The NECC used state-wide cancer registries and hospital tumour boards to identify 2,040 cases of epithelial ovarian cancer from eastern Massachusetts and New Hampshire from 1992 to 2008^23^. Women were ineligible if they were younger than age 18, did not have a telephone, did not speak English, moved, died, had a prior bilateral oophorectomy or their physician declined permission to contact. The institutional review boards at Brigham and Women’s Hospital and Dartmouth Medical School approved the study protocol, and each participant provided written informed consent.

The NHS and NHSII requested paraffin-embedded tissue blocks containing representative tumour samples from cases of epithelial ovarian cancer with a pathology report. The NECC accessed tumour blocks from patients, most of whom were diagnosed at Brigham and Women’s Hospital. In NECC, funding was available to obtain tissue blocks for only a subset of cases, oversampling high-grade serous tumours. Tissue blocks were reviewed to verify histology and grade and make TMAs. TMAs were arrayed at the Dana–Farber/Harvard Cancer Center Specialized Histopathology Core by taking at least three, and up to six, core biopsies with a 1.0 mm (NECC) or 0.6 mm (NECC, NHS and NHSII) diameter from ovarian cancer tissue blocks and re-embedding the cores into a single block. All specimens of HGSOC used and analysed in this study are described in Supplementary Table 10.

### Cell lines

OVCAR3, A549, NIH-H23, HEK-293T, K562 and THP1 cells were purchased from ATCC; OVCAR4, OVCAR5 and OVACR8 cells were procured from National Cancer Institute; Kuramochi cell line was procured from JCRB Cell Bank. Human OSE cells were purchased from ScienCell Research Laboratories (no. 7310). We transduced OVCAR3 tumour cells with a luciferase-expressing vector (OVCAR3^luci^). To make OVCAR3 cells as target of NY-ESO-1-specific T cells, OVCAR3^luci^ cells were transduced to express NY-ESO-1 (OVCAR3^luci^-NY-ESO-1). Tumour-sorted CD45^−^EpCAM^+^ primary HGSOC cells were cultured continuously in R10 medium (RPMI-1640, 10% FBS, penicillin (100 IU ml^−1^), streptomycin (100 μg ml^−1^), l-glutamine (2 mM), sodium pyruvate (0.5 mM)) (ThermoScientific) until they adhere and grow similar to a cell line. All cell lines, except OSE, were routinely cultured in R10 medium. OSE cells were routinely cultured in recommended complete medium, purchased from ScienCell Research Laboratories. Cell lines routinely tested for negative mycoplasma contamination.

### CRISPR–Cas9-mediated ablation of *PIGR* in OVCAR3 cells

CRISPR RNA (crRNA) targeting *PIGR* 5′-CUUCACAACAGAGCGACGAUGUUUUAGAGCUAGAAA-3′ (IDT) was reconstituted to make 100 μM in nuclease-free duplex buffer (IDT), and then mixed at equimolar concentration with Alt-R CRISPR–Cas9 *trans*-acting crRNA (tracrRNA), Atto550 (IDT) in a sterile PCR tube. Guide RNA was prepared by annealing crRNA and tracrRNA duplexes by heating at 95 °C for 5 min in PCR thermocycler, followed by gradual slow cooling to room temperature. Nine μl of crRNA–tracrRNA duplexes was mixed with 6 μl (180 pmol) of TrueCut Cas9 Protein v.2 (Invitrogen), followed by incubation at room temperature for 10 min to form Cas9 ribonucleoproteins (RNPs). OVACR3 or primary human HGSOC cells were suspended into final concentration of 5 × 10^6^ cells per ml. Fifteen microlitres of the Cas9 RNPs was added to 100 μl of the cell suspension and electroporated using Neon Tranfection System (ThermoFisher), performed at 1170 V, 30 ms for 2 pulses. Cells were then cultured in R10 medium. Electroporation was confirmed by analysing Atto500 (tracrRNA) signal by flow cytometry on the next day, and loss of pIgR from the OVACR3 cells or primary HGSOC cells was confirmed by western blot five days later.

### Mice and tumours

Female, 4–6-week-old *Rag1*-deficient (*Rag1*^*−/−*^) mice and NSG mice of the same age group were procured from Charles River Laboratories and Jackson Laboratory, respectively; and maintained by the animal facility of H. Lee Moffitt Cancer Center, with a 12 light/12 dark cycle, at about 18–23 °C with 40–60% humidity. Mouse experiments were approved by the Institutional Animal Care and Use Committee at the University of South Florida.

Flank tumours were initiated by injecting 1 × 10^7^ OVCAR3 or autologous primary human HGSOC cells (wild-type or *PIGR*-ablated) cells. Tumour volume was calculated as: 0.5 × (*L* × *W*^2^), in which *L* is length and *W* is width. Tumour tissues were dissected mechanically into single-cell suspensions for flow cytometry, or retained for RNA and protein isolation.

Intratumoural or peritumoural injections of antibodies were done on several days, starting from day 7 after the tumour challenge, at a dose of 100 μg per 20 g body weight.

Natural killer cells were depleted from RAG1-deficient mice by intraperitoneal injections of 200 μg of NK1.1-neutralizing antibodies (anti-NK1.1, BioXCell, PK136, BE0036) 3 days before tumour challenge, followed by 100-μg injections on every 3 or 4 days.

Tumour volumes in mice were measured using code names on the cages and ear tags, instead of specific information about the treatments that the mice received. Apart from this, no blinding method was used for mouse studies.

### Ovarian-cancer-sorted B cell immortalization, antibody purification, proteome array and pepsin digestion

Cryopreserved single-cell suspensions of HGSOC were thawed in 37 °C water bath followed by clearing of the cryoprotectant medium by centrifugation. After performing annexin V^+^ dead cell removal, viable single-cell samples were bead-sorted with human CD19 microbeads (Miltenyi Biotec, 130-050-301), according to manufacturer’s recommendation. Isolated CD19^+^ B cells were counted and 2 × 10^5^ B cells per ml R10 were expanded for 5 days using human B cell expansion reagents (R&D Systems, CDK005), according to manufacturer’s recommendation. Expanded B cells were then challenged with Epstein–Barr virus (ATCC-VR-1492) added at 1:10 ratio, and kept in R10 without expansion supplements. In 2 to 3 weeks, most of the Epstein–Barr-virus-infected B cells had died; very few sustained the challenge and become immortalized. Immortalized B cells were grown to confluency. The conditioned medium was collected and concentrated using filters (Millipore Amicon, UFC900325). Part of the concentrated supernatants was analysed for reactivity against human protein antigens, using a proteome array that includes about 81% of all human proteins spotted into glass slides (CDi Lab).

Specific antigen-reactive B cells were FACS-sorted using tetramers, prepared by biotinylated peptides (GenScript) and PE-labelled streptavidin (BioLegend, 405203), and grown. Peptides were added from a 5.0 mg ml^−1^ stock to B cells in suspension at a final concentration of 1.0 μg per 10^7^ B cells in 200 μl medium and incubated for 30 min at 4 °C, followed by a wash with PBS and incubation with streptavidin–PE added at 1:20 dilution. We used the peptides HGIPPSCCMNETDCNP and TQSYVRALTMDSKKRI to sort TSPAN7- and BDNF-specific B cells, respectively, from the pool of immortalized B cells.

Sorted cells were diluted in fresh medium and distributed in round-bottom 96-well plates in a way such that each well received one cell. Additional 10,000 B cells, irradiated with 100 Gy X-ray and 30 min UV, were added to each well as feeder cells. Then individual clones of B cells were grown over a period of 1 month. Conditioned medium was collected, concentrated using Amicon filters, and from the concentrated medium, human IgA or IgG were purified using purification kits (LigaTrap, LT-146KIT and LT-095KIT) according to recommended protocols.

To prepare F(ab′)_2_ fragments, Fc regions of human IgA or IgG antibodies were pepsin-digested using a pepsin digestion kit (ThermoScientific, 44988) according to the manufacturer’s recommendation.

### Cytotoxicity assay

CD3 T cells were bead-sorted from peripheral blood lymphocytes and transduced with a retroviral construct of NY-ESO-1 TCR or human FSH–CER^14^. NY-ESO-1 TCR was made using a sequence corresponding to an HLA-A2-restricted TCR that recognizes SLLMWITQC, corresponding to residues 157 to 165 of NY-ESO-1^13^ (publicly available http://www.google.com/patents/US8143376), was purchased from IDT and ligated into pBMN-I-GFP. In luciferase-compatible 96-well plates, 10,000 OVCAR3^luci^-NY-ESO-1 or OVCAR3^luci^ cells were placed. After 4 h wells were washed, fresh medium was added and cells were treated with non-specific, native human IgA (Abcam, ab91025) or IgG (Abcam, ab98981) at 0.5 μg ml^−1^ final concentration. After 2 h incubation at 37 °C, we added the appropriate number of T cells per well in a final volume of 200 μl. Cells were incubated for 4 h or 16 h in a 37-°C incubator, and checked for cytotoxicity using the luciferase assay (Promega).

For the autologous cytotoxicity assay, total CD3^+^ T cells, CD19^+^ B cells and CD45^−^EpCAM^+^ tumour epithelial cells were sorted after removal of dead cells. The B cells were immortalized, and IgA or IgG antibodies from concentrated supernatants of growing B cell culture-conditioned medium were separated as described in ‘Ovarian-cancer-sorted B cell immortalization, antibody purification, proteome array and pepsin digestion’. The tumour cells were grown in R10 medium to make continuous cultures. Ten thousand tumour cells were placed in 96-well plates. After 4 h wells were washed, fresh medium was added and cells were treated with autologous B-cell-derived whole or pepsinized IgA, autologous IgG, or irrelevant whole or pepsinized human IgA at 0.5 μg ml^−1^ final concentration. After 2 h incubation at 37 °C, we added 10,000 T cells per well in a final volume of 200 μl. Cells were incubated for 16 h in a 37-°C incubator. Total apoptotic cells (annexin V^+^) and viable cells (annexin V^−^propidium iodide^−^) were analysed by flow cytometry.

Wild-type or *PIGR*-ablated OVACR3 cells were incubated with irrelevant IgA or IgG for 2 h, FSH–CER T cells were added (1:1) and after 16 h incubation in a 37-°C incubator, total apoptotic cells (annexin V^+^) and viable cells (annexin V^−^propidium iodide^−^) were analysed by flow cytometry.

Ten thousand OVCAR3^luci^ cells were placed in 96-well plates and after 4 h wells were washed followed by incubation with or without anti-TSPAN7–IgA (0.5 μg ml^−1^) antibodies for 2 h at 37 °C, and then splenic myeloid cells from tumour-bearing mice, pre-incubated with isotype or anti-CD351 blocking antibody for 30 min in ice, were added to the OVACR3 cells at 1:1 ratio and incubated for 12 h, and checked for cytotoxicity using the luciferase assay (Promega).

Cytotoxicity was calculated as (maximum viability control – individual well)/(maximum viability control – maximum death control) × 100, as a percentage.

### Multiplex immunohistochemistry

**Extended Data Fig. 7:**
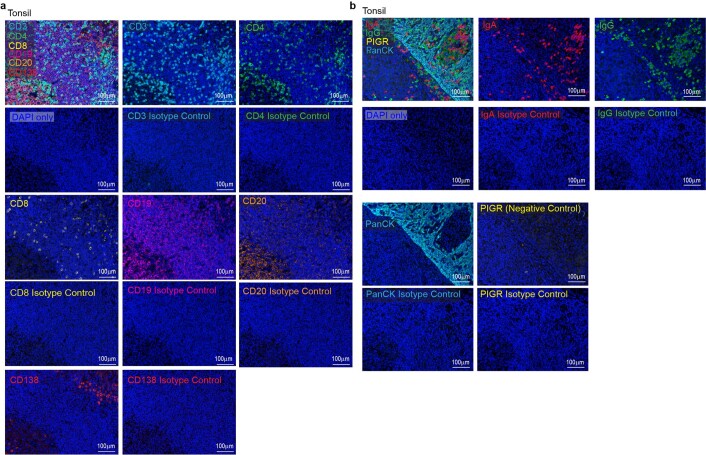
Multiplex immunohistochemistry staining optimization for CD3, CD4, CD8, CD19, CD20, CD138, IgA, IgG, pIgR and PCK in tonsil tissues. **a**, **b**, Multiplex staining of tonsil tissue sections (*n* = 5) with anti-human CD3, CD4, CD8, CD19, CD20 and CD138 antibodies or respective isotype controls (**a**), and IgA, IgG, pIgR and PCK antibodies or respective isotype controls (**b**). Scale bar, 100 μm.

**Extended Data Fig. 8:**
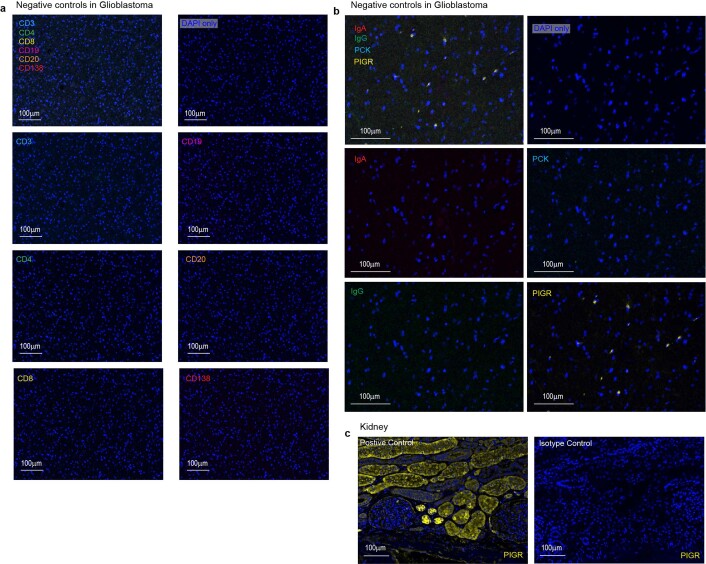
Multiplex immunohistochemistry staining optimization for CD3, CD4, CD8, CD19, CD20, CD138, IgA, IgG, pIgR and PCK in glioblastoma tissues, and for pIgR in kidney tissues. **a**, **b**, Multiplex staining of glioblastoma tissue sections (*n* = 5) with anti-human CD3, CD4, CD8, CD19, CD20 and CD138 antibodies (**a**), and IgA, IgG, pIgR and PCK antibodies (**b**). Scale bar, 100 μm. **c**, Multiplex staining of healthy kidney tissue sections (*n* = 4) with anti-human pIgR antibody and respective isotype control. Scale bar, 100 μm.

FFPE TMAs were immunostained using the PerkinElmer OPAL 7-Colour Automation IHC kit on the BOND RX autostainer (Leica Biosystems) and the following anti-human antibodies: CD3 (Dako, A0452, 1:100), CD4 (Cell Marque, EP204, 104R-25, 1:100), CD8 (Dako, C8/144B, M7103,1:800), CD19 (Dako, LE-CD19, M7296, 1:50), CD20 (Dako, L26, M0755, 1:800), CD138 (Dako, MI15, M7228, 1:500), pIgR (Abcam, ab96196, 1:100), IgA (Abcam, EPR5367-76, ab124716, 1:1000), IgG (Abcam, EPR4421, ab109489, 1:500), and pan-cytokeratin (PCK, Dako, AE1/AE3, M3515, 1:200). Nuclei were stained with DAPI. Precisely, tissues were baked at 65 °C for 2 h then transferred to the BOND RX (Leica Biosystems) followed by automated deparaffinization, and antigen retrieval using OPAL IHC procedure (PerkinElmer). Autofluorescence slides (negative control) were included, which use primary and secondary antibodies omitting the OPAL fluorophores. Slides were scanned and imaged with the PerkinElmer Vectra3 Automated Quantitative Pathology Imaging System. Multilayer TIFF images were exported from inForm v.2.4.8 (PerkinElmer) and loaded into HALO v.3.0.311.328 (Indica Labs) for quantitative image analysis. Each fluorescent fluorophore is assigned to a dye colour and positivity thresholds were determined per marker on the basis of published nuclear or cytoplasmic staining patterns. Quantifications in tumour islets and stroma were distinguished by PCK staining. Datasets were exported with cytoplasmic, nuclear and total cell counts for each fluorescent marker from the sample set. Cell segmentation files were generated through inForm and dot plots were generated and analysed by FCS Image v.7.0. Standardization of multiplex immunohistochemistry staining experiments with appropriate positive and negative control tissues, including isotype control antibodies, are summarized in Extended Data Figs. 7, 8. Precisely, human tonsil sections were used as a positive control for CD3, CD4, CD8, CD19, CD20, CD138, IgA, IgG and PCK, and as a negative control for pIgR. Healthy kidney tissue sections were used as a positive control for pIgR. Glioblastoma sections were used as a negative control for CD3, CD4, CD8, CD19, CD20, CD138, IgA, IgG and PCK. Respective positive-control tissue adjacent sections were stained with isotype control antibodies to rule out false-positive staining.

### Microscopy

#### Antibody internalization

Whole or pepsinized, non-antigen-specific or specific, IgA or IgG antibodies were conjugated with APC-conjugation kit (Abcam). Fifty thousand OVACR3 cells (wild type or *PIGR*-ablated) were placed onto a coverslip within 6-well plates and after 12 h treated with the antibodies. After different hours of incubations, cells were washed, fixed and mounted using a DAPI-containing mounting reagent (CST). Images were captured in a confocal microscope (Leica SP8) using LAS X (v.3.5.5.19976) software. Quantitative acquisition was performed using Zeiss Imager Z2 upright microscope using ZEN 2.3 (blue edition) software. CZI image files were imported into Definiens Tissue Studio v.4.7 (Definiens) to quantify cellular CY5 intensity. The software was used to run nucleus and cell detection algorithms to segment each cell, nucleus and cytoplasm and calculate the mean CY5 intensity within in these compartments. Intensity and size thresholds were set to minimize false-positive detection caused by artefacts and background fluorescence.

#### TUNEL assay

Xenograft tumour FFPE sections were stained for TUNEL^+^ cells using TUNEL Alexa Fluor 647 Imaging assay kit (Thermo) according to the manufacturer’s recommendation. Tile images were captured with an ORCA-Flash 4.00 V3 CMOS camera (Hamamatsu Photonics K.K.) and Zen 2 Blue software (Carl Zeiss) and stored in CZI file format. The software was also used to stitch the image tiles into whole-slide images. Representative images were exported to 8-bit TIFF format. CZI image files were imported into Definiens Tissue Studio v.4.7 to quantify the number of TUNEL^+^ cells. The software was used to run a nucleus detection algorithm on the CY5 channel of the whole-slide image. Intensity and size thresholds were set to minimize false-positive detection caused artefacts and background fluorescence. An adjacent section for each tumour was stained with haematoxylin and eosin, scanned using automated slide scanner (Aperio-Leica Scanner Console (v.102.0.7.5)) and the images was examined for necrotic holes within the tissues. Each tissue section was manually annotated to determine the area of the entire tissue section and large necrotic holes. The number of TUNEL^+^ cells was normalized by total tissue area minus large necrotic hole areas that appear in many of the tumour sections.

#### Antibody uptake analysis in xenograft tumours

Xenograft-tumour FFPE sections were rehydrated, antigen was retrieved, blocked and incubated with anti-human IgA antibodies overnight, followed by incubation with Alexa Fluor 647-conjugated secondary antibodies (CST, 4414) and mounted. CZI image files were imported into Definiens Tissue Studio v.4.7 to quantify the number of positive foci in the tissue. The software was used to run a nucleus detection algorithm on the DAPI channel and a spot detection algorithm on the CY5 channel. Intensity and size thresholds were set to minimize false-positive detection caused by artefacts and background fluorescence. The number of positive foci per tissue was normalized by total number of nuclei.

### Flow cytometry

Flow cytometry was performed by staining with Zombie NIR (BioLegend) or Zombie Yellow (BioLegend) or DAPI (ThermoScientific) viability dye, blocking with anti-CD16/32 (BioLegend), and staining for 30 min at 4 °C with the following anti-human antibodies: CD45 (BD Biosciences, HI30, 1:300), CD3 (BD Biosciences, SK7, 1:200), CD19 (BD Biosciences, HIB19, 1:200), CD20 (BioLegend, 2H7, 1:200), CD38 (BD Biosciences, HIT2, 1:200), CD138 (BioLegend, MI15, 1:200), CD27 (BD Biosciences, M-T271, 1:200), IgA (Tonbo Biosciences, 35-8016-M001, 1:20), IgG (BioLegend, M1310G05, 1:200), IgM (BioLegend, MHM-88, 1:200), IgD (BD Biosciences, IA6-2, 1:100), IgE (BD Biosciences, G7-26, 1:100), EpCAM (BD Biosciences, KS1/4, 1:200), pIgR (ThermoScientific, PA5-35340, 1:50) or tetramers against TSPAN7 or BDNF. For intracellular staining for immunoglobulin isotypes, cells were first incubated with surface staining antibodies (30 min in ice), followed by fixation (30 min at room temperature) (eBioscience) and finally incubation with the antibodies for intracellular markers in the permeabilization buffer (eBioscience) (45 min at room temperature).

**Extended Data Fig. 9:**
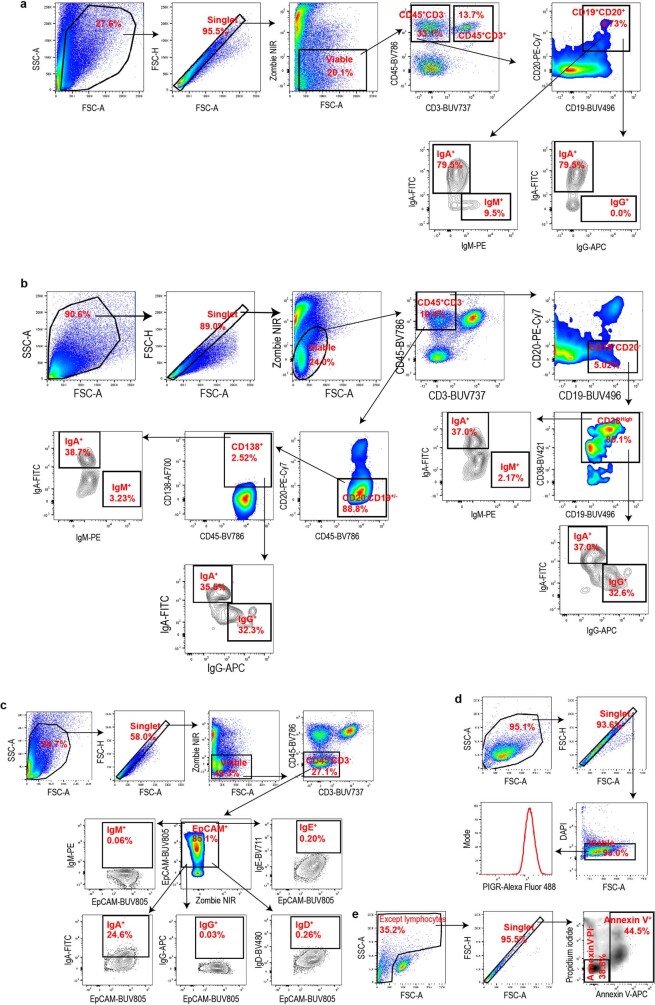
Flow cytometry gating strategies. **a**, **b**, Gating strategies for FACS analysis shown in Fig. 1a, Extended Data Figs. 1g, h, i, 2a, d, e. **c**, Gating strategies for FACS analysis shown in Fig. 1b. **d**, Gating strategies for FACS analysis shown in Fig. 1d. **e**, Gating strategies for FACS analysis shown in Fig. 1g, Extended Data Fig. 4j, k.

**Extended Data Fig. 10:**
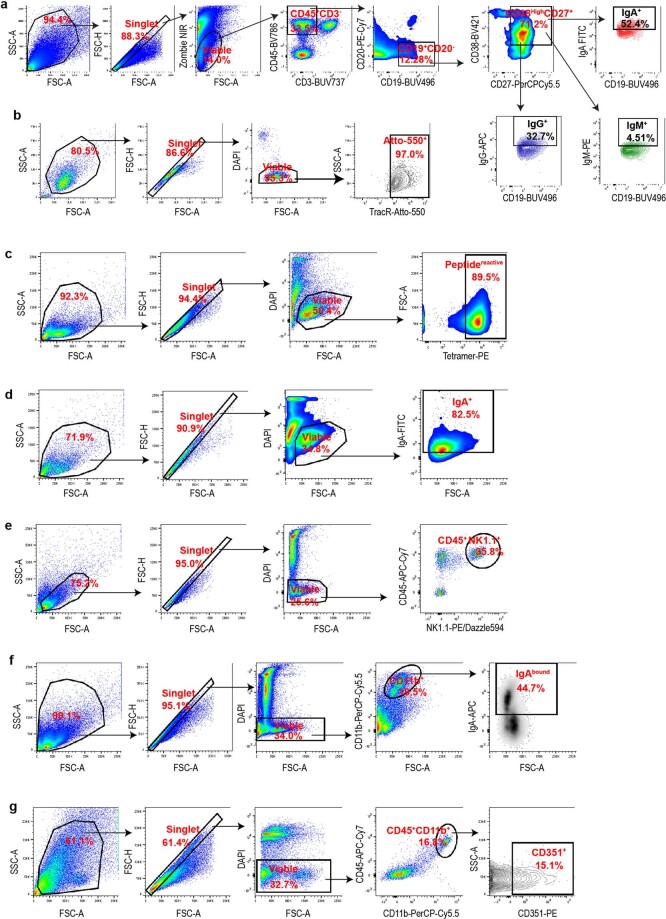
Further flow cytometry gating strategies. **a**, Gating strategies for FACS analysis shown in Extended Data Fig. 2a. **b**, Gating strategies for FACS analysis shown in Extended Data Fig. 3c. **c**, Gating strategies for FACS analysis shown in Extended Data Fig. 5f. **d**, Gating strategies for FACS analysis shown in Extended Data Fig. 5g. **e**, Gating strategies for FACS analysis shown in Extended Data Fig. 6a. **f**, Gating strategies for FACS analysis shown in Extended Data Fig. 6b. **g**, Gating strategies for FACS analysis shown in Extended Data Fig. 6c.

Mouse xenograft-tumour single-cell suspensions or splenocytes were blocked with Fc blocker (BioLegend) and analysed by flow cytometry after incubation for 30 min at 4 °C with following anti-mouse antibodies: CD45 (BioLegend, 30-F11), CD11b (BioLegend, M1/70), CD351 (BioLegend, TX61) or with APC-conjugated human IgA. Splenocytes from RAG1-deficient mice were mechanically dissociated and red blood cells were removed, followed by neutralization of Fcα/μ receptor (Fcα/μR) by incubation with CD351-neutralizing antibodies (BioLegend, TX61, 137303) or with isotype controls (BioLegend, 400123) at a concentration of 2.0 μg per 10^6^ cells in 100 μl volume for 30 min in ice. After washing, splenocytes were then incubated with APC-conjugated human IgA for another 30 min in ice and analysed by flow cytometry. Appropriate isotype controls and fluorescence minus one were run. Samples were subsequently run using BD FACS LSRII or sorted using BD FACS ARIA. Data were collected using BD FACS Diva v.8.0.1 and analysed using FlowJo v.10.7.1. Gating strategies used for flow cytometry analyses are summarized in Extended Data Figs. 9, 10.

### RNA sequencing

OVCAR3 cells in culture in 2% FBS-containing RPMI medium were treated with or without 0.5 μg ml^−1^ of natural human IgA or IgG for 24 h. Total RNA was isolated from cultured cells using RNA isolation kit (Qiagen) and analysed for RINe. An Illumina NextSeq 550 instrument was used to generate 75-basepair paired-end RNA-sequencing (RNA-seq) reads. Base calls were converted to FASTQ files using bcf2fastq (v.2.20). Paired-end RNA-seq reads were aligned to the GRCh37 human reference genome using STAR^24^ (v.2.5.3a) following adaptor trimming by cutadapt^25^ (v.1.8.1). For OVCAR3 cells, uniquely mapped reads were counted by feautreCounts^26^ (v.1.5.3) using Gencode V30 transcript annotations for human. Differential expression analysis was performed using DESeq2 (v.1.30.0)^27^. Heat maps were generated using *z*-score-transformed log_2_(1 + normalized count).

For each differential expression analysis comparing antibody treated groups with the untreated group, genes were ranked based on −log_10_(*P* value) × (sign of log_2_(fold change)). The preranked gene list was used to perform preranked GSEA^28^ (v.4.0.2) to assess enrichment of hallmarks, curated gene sets and Gene Ontology^29^ terms in MSigDB^28^. The resulting normalized enrichment score and false-discovery-rate-controlled *P* values were used to assess the IgA-induced transcriptome changes.

### IgA transcytosis and pIgR LC-MS/MS

One hundred thousand OVCAR3, OVCAR4, OVCAR5 or primary HGSOC-tumour cells were placed in 6-well plates and washed with PBS after 12 h before treating with or without brefeldin A (1 μg ml^−1^) or wortmannin (1 μM) in serum-free RPMI medium. After 4 h cells were treated with or without native human IgA (Abcam, ab91025) or IgG (Abcam, ab98981) at 0.5 μg ml^−1^ final concentration. After 12 h, conditioned medium was collected and filtered for contaminant debris removal. Proteins were extracted from the conditioned medium, reduced by DTT, digested by trypsin and subjected to LC–MS/MS analysis by the Moffitt Cancer Center Proteomics Facility. MaxQuant (v.1.5.2.8) was used to analyse the data, identify and quantify the proteins^30^.

### Western blot and co-immunoprecipation

Cells and mechanically dissociated tumour samples were lysed in RIPA buffer (ThermoScientific) with protease–phosphatase inhibitor cocktail (SigmaAldrich) and cleared by centrifugation. Proteins were quantified by BCA assay (ThermoScientific). Membranes were blotted with anti-pIgR (Abcam, ab96196, 1:500), anti-IgA (Abcam, EPR5367-76, ab124716, 1:2,500), anti-phospho-ERK1/2 (CST, D1H6G, 5726, 1:1,000), anti-ERK1/2 (CST, L34F12, 4696, 1:2,000), anti-DUSP5 (Abcam, EPR19684, ab200708, 1:1,000) and anti-β-actin (CST, 13E5, 5125 or 4970, 1:5,000) antibodies. For TSPAN7 and BDNF, isolated IgA antibodies from concentrated conditioned medium from TSPAN7- and BDNF-reactive B cells were used. Immunoreactive bands were developed using horse radish peroxidase-conjugated secondary antibodies (CST, 7074, 1:5,000; CST, 7076, 1:5,000; Abcam, ab97215, 1:5,000) and enhanced chemiluminescence substrate (GE HealthCare).

HGSOC tumour tissue chunks were pulverized and lysed using nonreducing nondenaturing lysis reagent provided in the co-immunoprecipitation kit (Pierce) used. Pellets of OVCAR3 cells, CD45^+^ and CD45^−^ fractions of human ovarian cancer ascites were also lysed with the same nondenaturing lysis reagent. Cell-free human ovarian cancer ascitic fluids were filtered, concentrated and diluted with the nondenaturing lysis reagent. Proteins were immunoprecipitated using anti-human IgA antibody (Abcam, EPR5367-76, ab124716, 1:20) and eluted following the manufacturer’s instructions. Elutes and inputs were immunoblotted for pIgR and IgA. Conditioned medium from transcytosis experiment in OVCAR3 (wild-type or *PIGR*-ablated) cells was concentrated and immunoprecipitated with anti-human IgA antibody and part of elutes were western-blotted for the secretory component of pIgR (Abcam, SC-05, ab3924, 1:500) or analysed by LC–MS/MS.

### Analysis of TCGA data

Molecular and clinical data from TCGA for ovarian serous cystadenocarcinoma (designated OV) were downloaded from the cBio Cancer Genomics Portal (http://www.cbioportal.org/), Broad Firehose website (https://gdac.broadinstitute.org/) and Genomic Data Commons Data Portal (https://portal.gdc.cancer.gov/). A total of 428 patients with matched clinical information and tumour RNA-seq data was used in this study. Raw RNA-seq reads were aligned to the GRCh37 human transcriptome using STAR^24^ (v.2.5.3a). Uniquely aligned reads were counted against Gencode v.19 using htseq-count^31^ (v.0.6.1) and then normalized using DESeq2^27^ taking into account batches and RNA composition bias. All statistical analysis and visualization was performed using log_2_-transformed normalized count. Kaplan–Meier survival analysis and the log-rank tests were performed to compare overall survival between groups.

### 10X Genomics single-cell V(D)J (BCR) sequencing and recombinant antibody production

Single-cell V(D)J (BCR) sequencing was performed using the 10XGenomics Chromium system. A single-cell suspension derived from HGSOC-sorted B cells was analysed for viability using the Nexcelom Cellometer K2 and then loaded onto the 10X Genomics Chromium Single Cell Controller at a concentration of 1,000 cells per μl to encapsulate around 5,000 cells per sample. In brief, the single cells, reagents and 10X Genomics gel beads were encapsulated into individual nanolitre-sized gel beads in emulsion and then reverse transcription of poly-adenylated mRNA was performed inside each droplet. The cDNA libraries were then completed in a single bulk reaction using the10X Genomics Chromium NextGEM Single Cell V(D)J Reagent Kit and enriched for B cell receptor sequences using the VDJ primers provide in the kit. Following PCR purification, fragment DNA libraries were prepared according to the 10X Genomics protocol. The V(D)J enriched libraries were sequenced to at least 5,000 reads per cell on the Illumina NextSeq 500 instrument. Demultiplexing, barcode processing, alignment and gene counting were performed using the 10X Genomics CellRanger V(D)J (v.3.1.0) software.

BCR reads sequenced by V(D)J assay were aligned to GRCh38 reference transcriptome using Cell Ranger VDJ (v.3.1.0, 10X Genomics). BCR heavy and light chains were assembled and annotated using cellranger vdj with --reference = refdata-cellranger-vdj-GRCh38-alts-ensembl-3.1.0 to determine clonotypes. Recombinant dimeric IgA antibodies were produced by Genscript. In brief, corresponding DNA sequences for immunoglobulin heavy chain, light chain and J chain were synthesized, and the complete sequence was subcloned into pcDNA3.4 vector and expressed in HD 293F cells. Dimeric IgA1 antibodies were eluted from cell culture supernatants. Molecular weight and purity were analysed by SDS–PAGE and high-performance liquid chromatography.

### Statistics and reproducibility

Unless mentioned otherwise, all data are presented as mean with s.e.m. Two-tailed *t*-tests (unpaired and paired, as appropriate) were performed between two groups, and one-way ANOVA were performed for comparisons between more than two groups, unless indicated otherwise. Pearson correlation analysis was performed for correlation analysis. To compare IgA^+^ and IgG^+^ or IgM^+^ cell percentage in B cells, plasmablasts and plasma cells, two-way ANOVA followed by Dunnett’s ad hoc tests for multiple comparison was performed on arcsine-transformed percentage data (IgA versus IgG or IgM, *P* value = 1 × 10^−10^). Analyses were carried out in Graph Pad Prism (v.7.0) or R (v.3.6.1) software. A significance threshold 0.05 for *P* values was used. Box plots were generated using gglot2 in R (3.6.1) with following parameters: horizontal black lines with each box present median values; boxes extend from 25th to 75th percentile of values; whiskers extend to a maximum of 1.5 × interquartile range (75th percentile–25th percentile) beyond the boxes; the lowest dots are the minimum values and highest dots are the maximum values for each box. All experiments are represented by several biological replicates or independent experiments, unless otherwise mentioned. The number of replicates per experiment is indicated in the legends. Bar plots are mean ± s.e.m. with overlaid data points representing independent experiments or replicates. All western blot analyses were independently replicated at least two times in case of human-derived specimens and three times in case of in vitro experiments, with similar results. Co-immunoprecipitation experiments were independently replicated a minimum two times with similar results. For all multiplex immunohistochemistry of TMAs, individual tumours have replicated cores (2–6, from different areas of the tumour) on the TMAs, and averaged quantification values from replicated cores were used. The TMA slides were scanned twice, analysed, and used with <1% data variation. CRSIPR-mediated knockdown experiments were repeated three times independently. Additional information and test results of statistical analysis are provided in the figure legends and Supplementary Tables 1–12. No sample size calculations were performed before the study for human specimens. For most functional in vitro analyses, sample sizes were chosen on the basis of the availability of target cells. Mouse experiments used at least five mice per group per experiments. Because this study focuses on ovarian cancer, only female mice were included in the experimental design. The experiments were not randomized. HGSOC tumour and ascites specimens were obtained from de-identified patients and were not randomized. Peripheral blood mononuclear cells from de-identified donors without cancer were acquired and analysed. Mice were not intentionally randomized. Tumour volumes in mice were measured using code names on the cages and ear tags, instead of specific information about the treatments that the mice received. Apart from this, no blinding method was used for mouse studies. RNA-seq, BCR sequencing, multiplex immunohistochemistry quantifications, fluorescence microscopy quantifications or LC–MS/MS were performed with unidentifiable demarcation. In case of in vitro experiments, samples were often assigned code numbers to facilitate blinded flow cytometry, microscopy and luciferase assay. After all data were collected, the results were analysed and decoded. For analysis of human specimens, blinding is not applicable as no interventions were tested.

### Reporting summary

Further information on research design is available in the Nature Research Reporting Summary linked to this paper.

## Online content

Any methods, additional references, Nature Research reporting summaries, source data, extended data, supplementary information, acknowledgements, peer review information; details of author contributions and competing interests; and statements of data and code availability are available at 10.1038/s41586-020-03144-0.

## Supplementary information

Supplementary Information This file contains Supplementary Fig. 1 (the uncropped western blots) and Supplementary Tables 1-12.

Reporting Summary

Supplementary Data 1 Peptides of the secretory component of pIgR detected in supernatants of OVCAR3 cells incubated with non-antigen specific IgA (A), but not in the presence of the transcytosis inhibitors wortmannin (A+WT) or brefeldin-A (A+BFA); or upon incubation with control IgG (G; G+WT; G+BFA); or with vehicle treatment (U). (*n* = 3).

Supplementary Data 2 Peptides of the secretory component of pIgR detected in supernatants of OVCAR4, OVCAR5 and primary HGSOC tumour cells incubated with non-antigen specific IgA, but not in the presence of the transcytosis inhibitors wortmannin or brefeldin-A; upon incubation with control IgG; or with vehicle treatment. (*n *= 3).

Supplementary Data 3 Peptides of the secretory component of pIgR detected in Co-immunoprecipitates of IgA from supernatants of IgA-treated pIgR^+^ (WT) OVCAR3 cells, but not in the presence of the transcytosis inhibitors wortmannin or brefeldin-A, and also not in supernatants of IgA-treated pIgR-CRISPR (CR) OVCAR3 cells. (*n *= 2).

Supplementary Data 4 RNA-seq analysis of OVCAR3 cells 8 h after incubation with irrelevant IgA, IgG or no treatment, *n *= 3/group. Data were processed as shown in Method. The normalized gene count, log2 fold-change, nominal *P *value, and FDR adjusted *P *value were shown for each gene.

Supplementary Data 5 Single cell BCR-seq.-RNA seq. analyses, performed with B cells sorted from two HGSOC.

Supplementary Data 6 Signals detected for IgA and IgG purified from supernatants of pooled B cells derived from 4 tertiary lymphoid structure (TLS)^+^ and 2 TLS^-^ HGSOCs. Four HuProt^TM^ arrays (v3.1; CDI) were used for the profiling assay of the samples. The supernatants were probed directly on the arrays without dilution and incubated 1 hour at room temperature with gentle shaking. After probing, the arrays were washed according to the protocol and probed with Alexa-647-anti-human IgG (Fc) or Cy3-anti-human IgA secondary antibodies under conditions optimized by CDI Labs for signal detection. **Data Analysis:**• Non-specific hits that directly bind to the secondary antibody were eliminated from the analysis of the samples. • CDI software was used to quantify the specificity of each individual sample to specific proteins on the array based on Z Scores. • Z score is the average Z score of the duplicate spots of a given protein (each protein is printed in duplicate on a HuProtTM array). The Z score of each spot on a given array is calculated according to the algorithm below: Z= [F532 or F635 – F532 or F635(avg)] / F532 or F635(std) F532 or F635(avg) and F532 or F635(std) are the average and standard deviation of the F532 or F635 values of all spots on the array, respectively. • S score is the difference of the Z Scores of a given protein and the one ranked next to it. • F532 or F635 is the average foreground signal intensity of 2 replicate spots of a given protein in the detection channel 532 nm or 635 nm. • B532 or B635 is the average background signal intensity of 2 replicate spots of a given protein in the detection channel 532 nm or 635 nm. • Range includes 3 numbers, the F532 or F635 values of the 2 replicate spots and the difference between them. If the difference is too high (compared to the F532 or F635 value), it indicates the 2 spots are not consistent and the hit may be less reliable.

Supplementary Data 7 RNA-seq analysis of OVCAR3 cells 8 h after incubation with αTSPAN7-IgA, α-BDNF-IgA or no treatment, *n* = 3/group. Data were processed as shown in Method. The normalized gene count, log2 fold-change, nominal *P* value, and FDR adjusted *P* value were shown for each gene.

## Data Availability

The RNA-seq data and single-cell BCR sequencing data related to this study are available at the NCBI Gene Expression Omnibus under accession number GSE146820. The mass spectrometry proteomics data are available in PRIDE with identifier PXD018079. Molecular and clinical data from The Cancer Genome Atlas for ovarian serous cystadenocarcinoma (OV) are available at the cBio Cancer Genomics Portal (http://www.cbioportal.org/), Broad Firehose website (https://gdac.broadinstitute.org/) and Genomic Data Commons Data Portal (https://portal.gdc.cancer.gov/). The datasets generated during the current study are available from the corresponding author upon reasonable request. Source data are provided with this paper.

## Source data

Source Data Fig. 1 Source Data Fig. 2 Source Data Fig. 3 Source Data Fig. 4 Source Data Extended Data Fig. 1 Source Data Extended Data Fig. 2 Source Data Extended Data Fig. 3 Source Data Extended Data Fig. 4 Source Data Extended Data Fig. 5 Source Data Extended Data Fig. 6
