# Supplementary material for: IgA transcytosis and antigen recognition govern ovarian cancer immunity
Source: Nature. 2021 Feb 3;591(7850):464–70. doi: 10.1038/s41586-020-03144-0 (PMC7969354; doi:10.1038/s41586-020-03144-0)
Supplement: Supplementary file 1 — This file contains Supplementary Fig. 1 (the uncropped western blots) and Supplementary Tables 1-12. [file 41586_2020_3144_MOESM1_ESM.pdf]

natureresearch

https://doi.org/10.1038/s41586-020-03144-0

Supplementary information

IgA transcytosis and antigen recognition govern ovarian cancer immunity

In the format provided by the authors and unedited

Supplementary Figure 1

Uncropped western blots

Fig1c_Center and Right

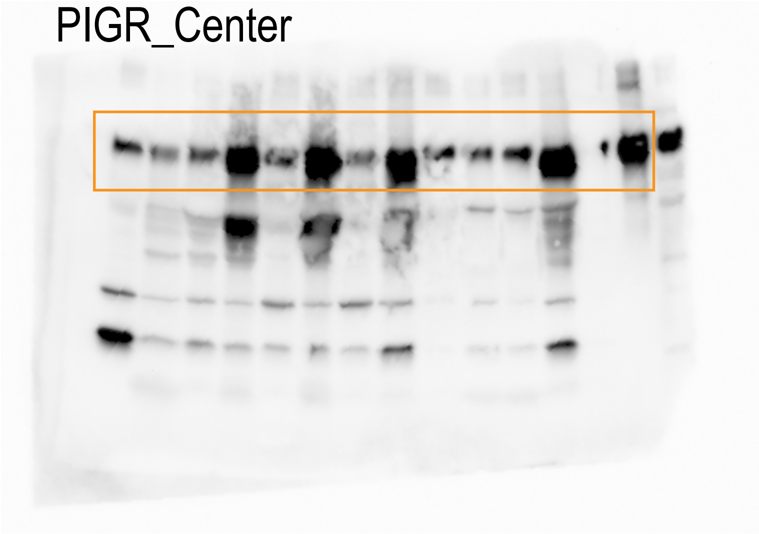

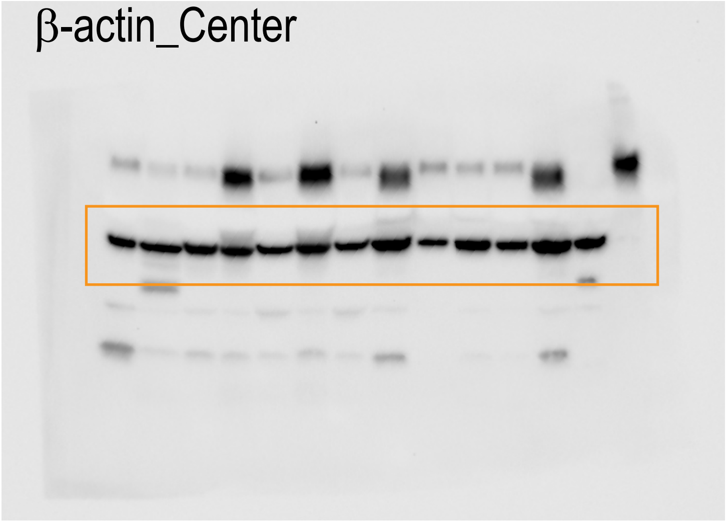

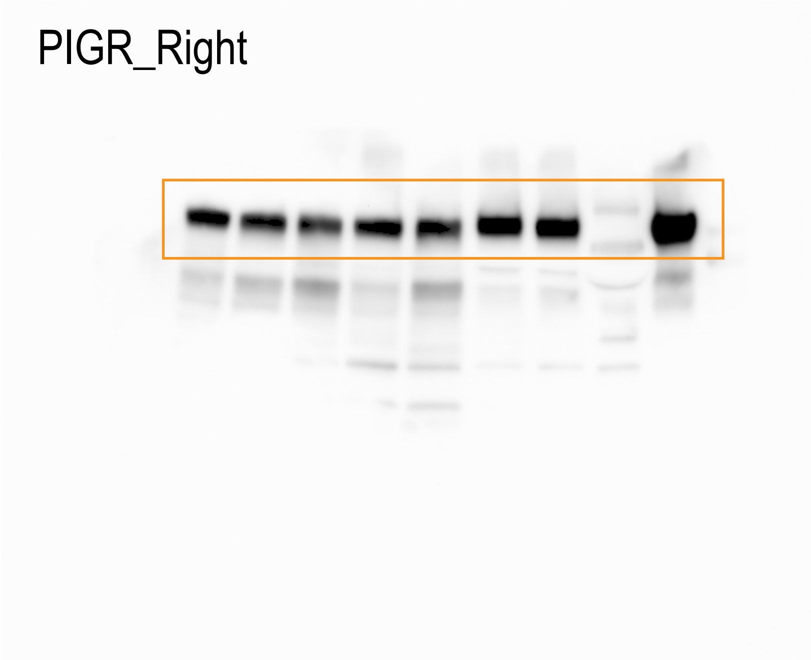

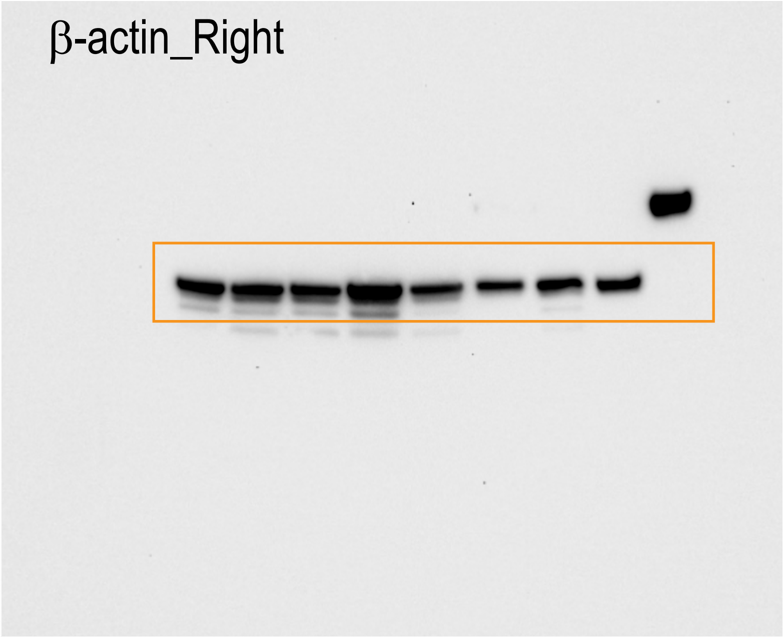

Fig1c_Left

PIGR_Left_Blot1

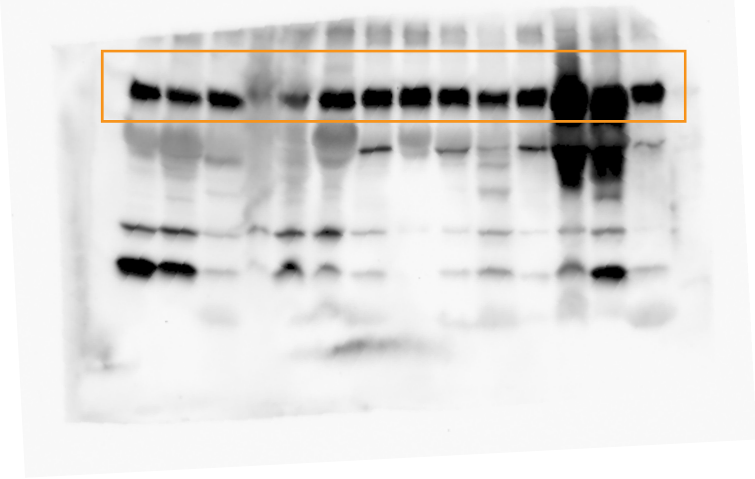

PIGR_Left_Blot2

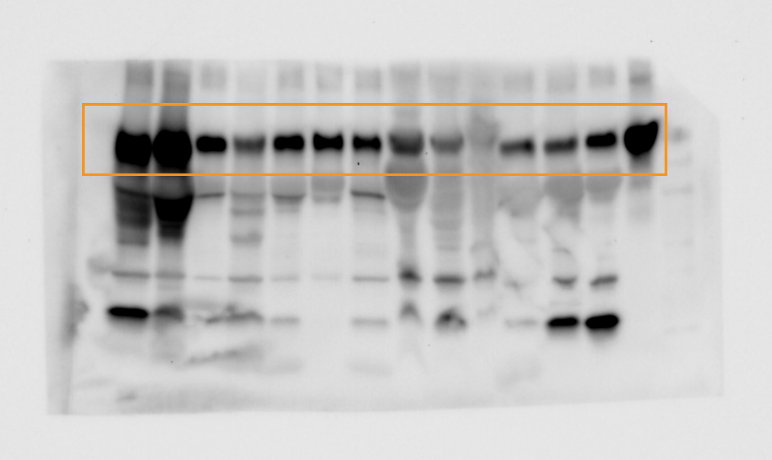

IgA _Left_Blot1

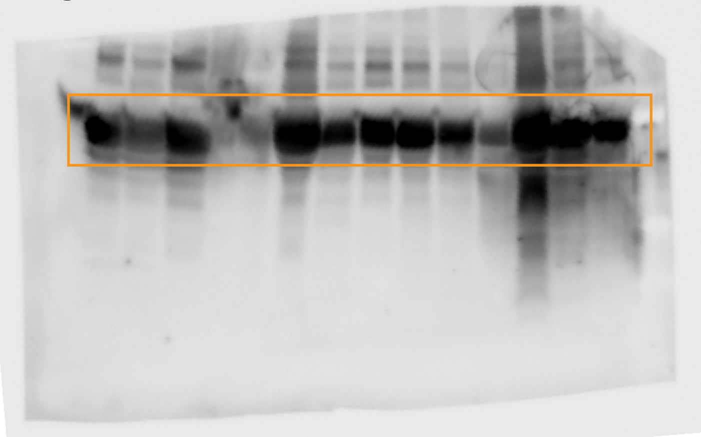

IgA _Left_Blot2

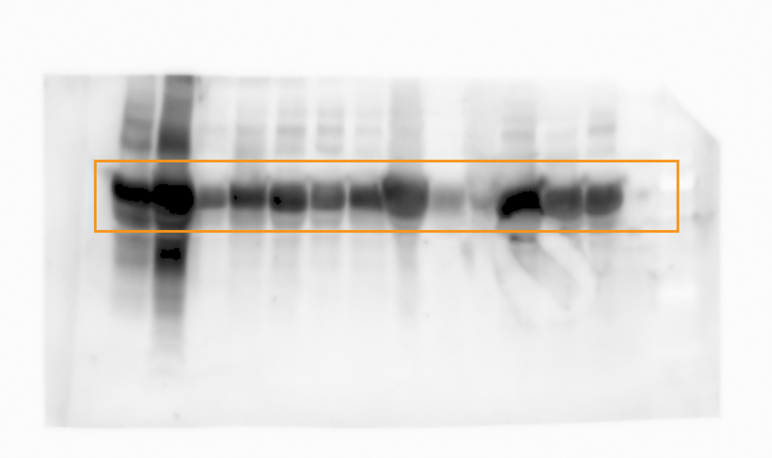

β–actin_Left_Blot1

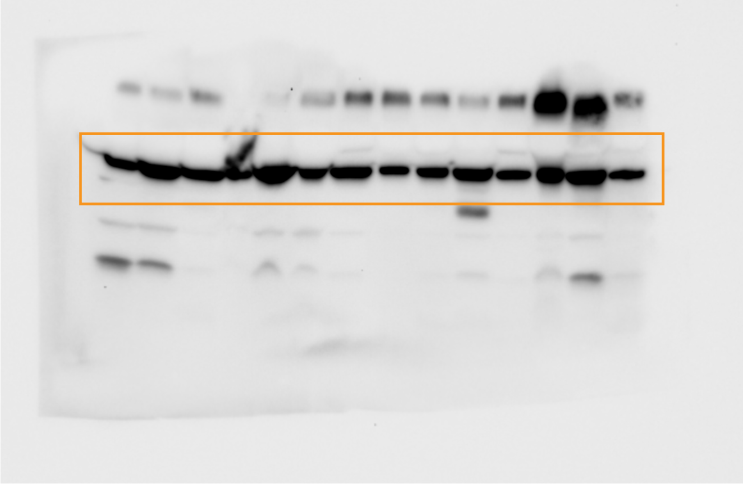

β–actin_Left_Blot2

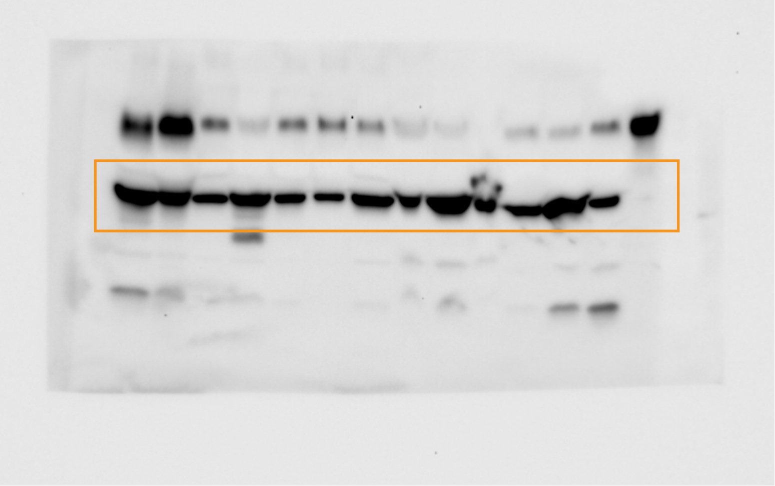

Fig. 2c

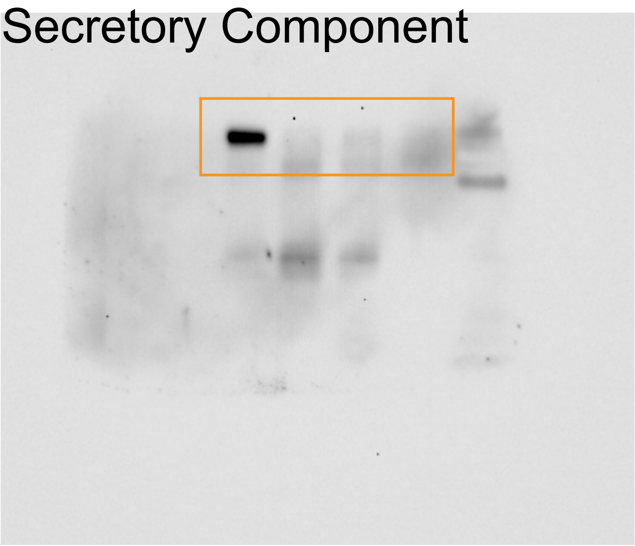

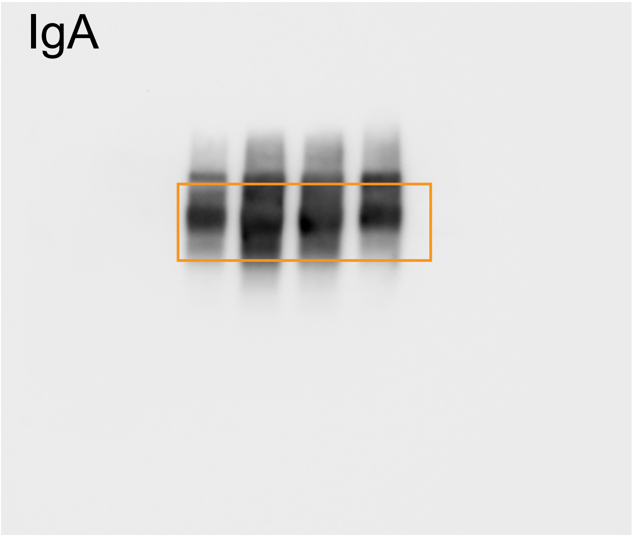

Fig.2e

Left panel (IgA treated OVCAR3)

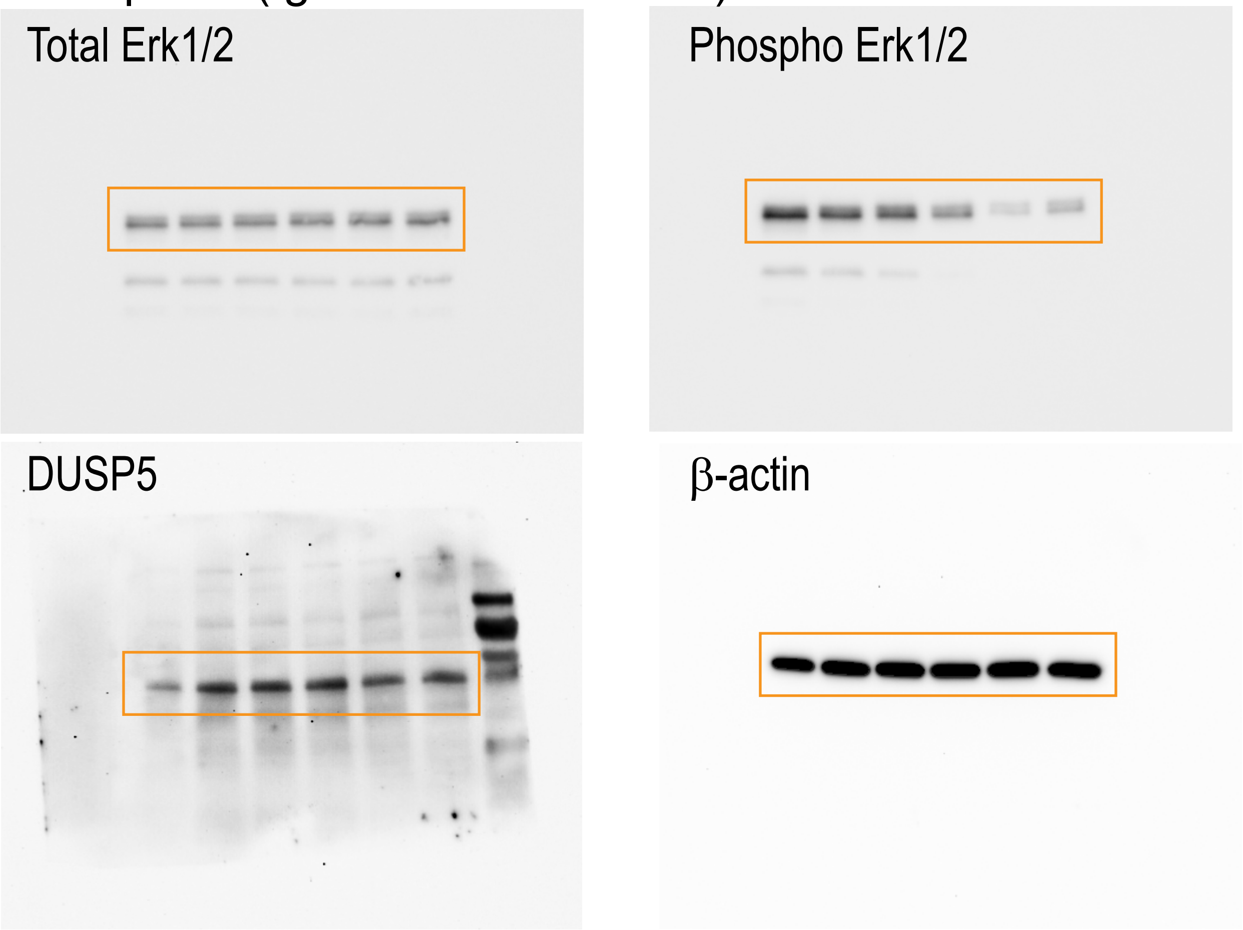

Right panel (IgG treated OVCAR3)

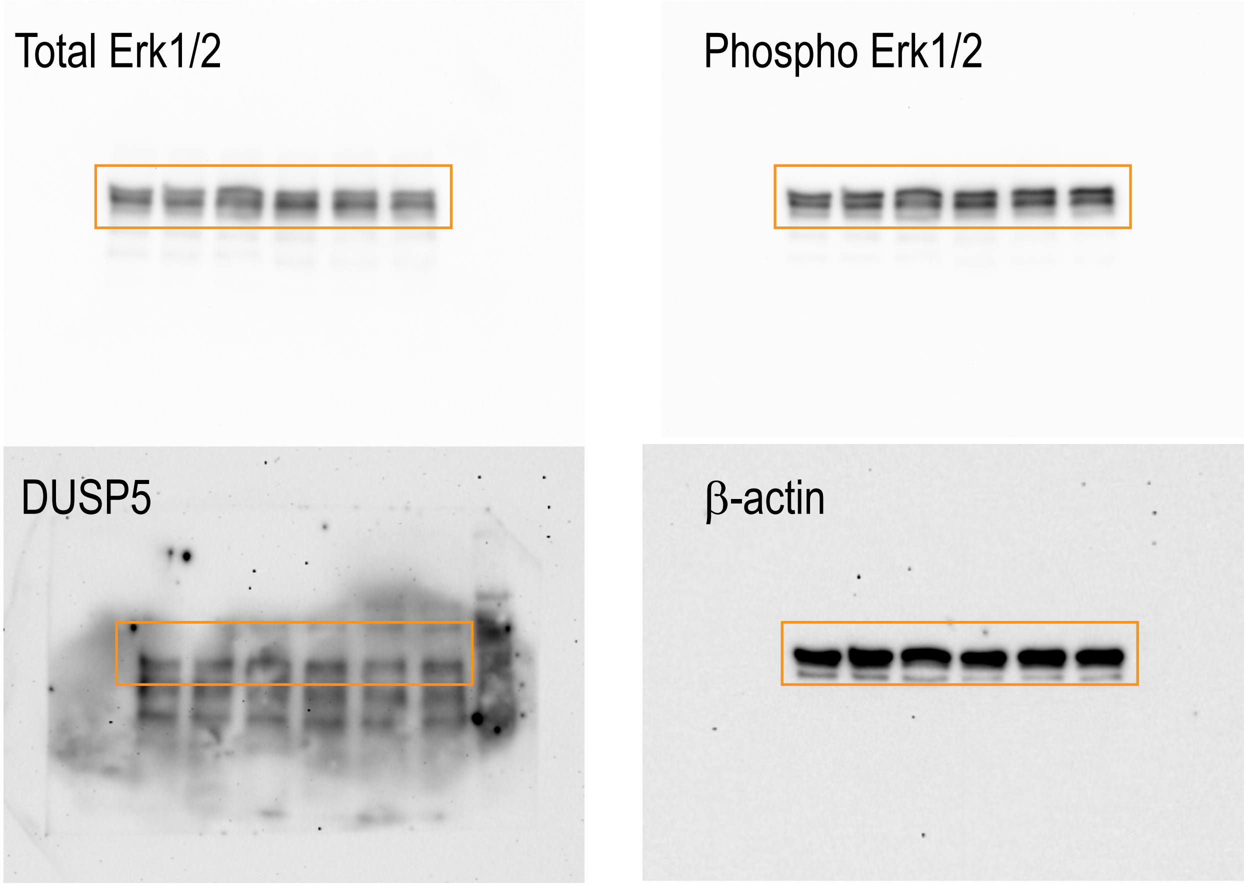

Fig.3c

Left panel

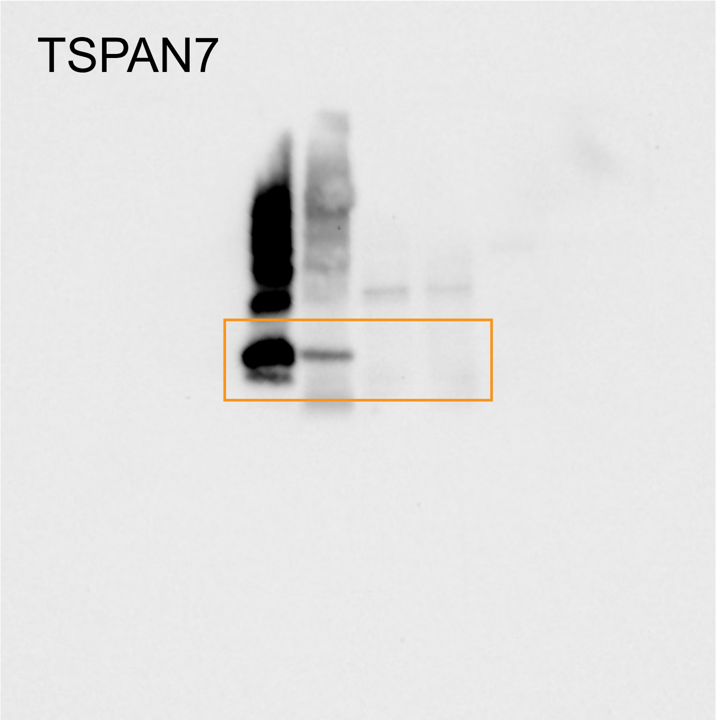

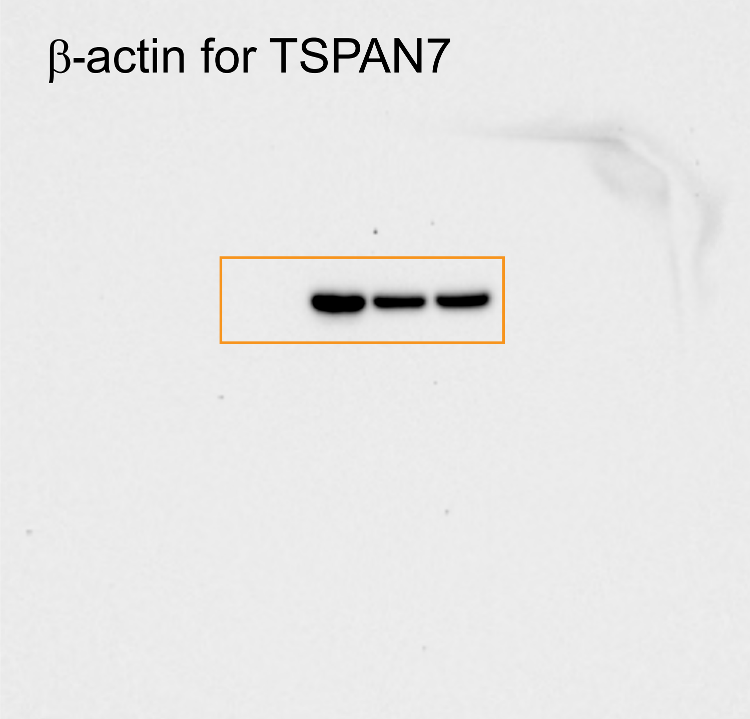

Right panel

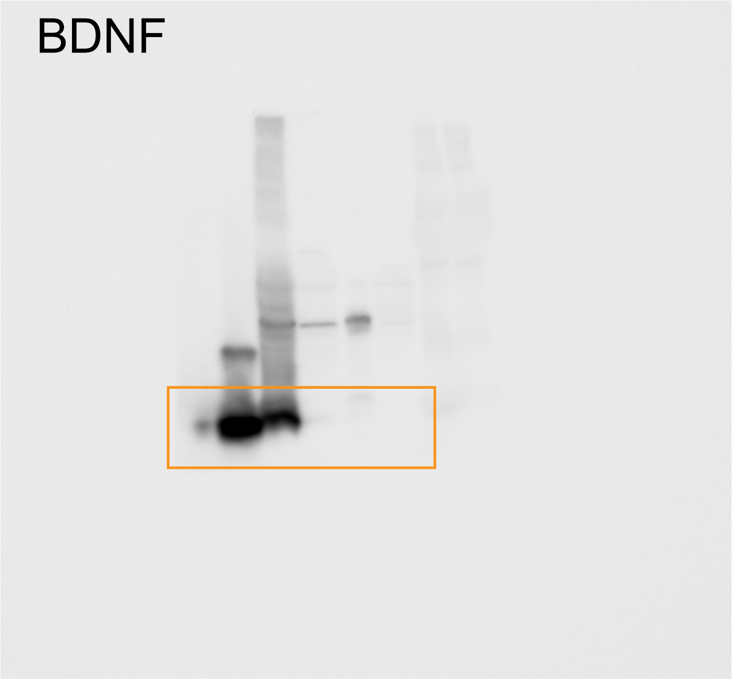

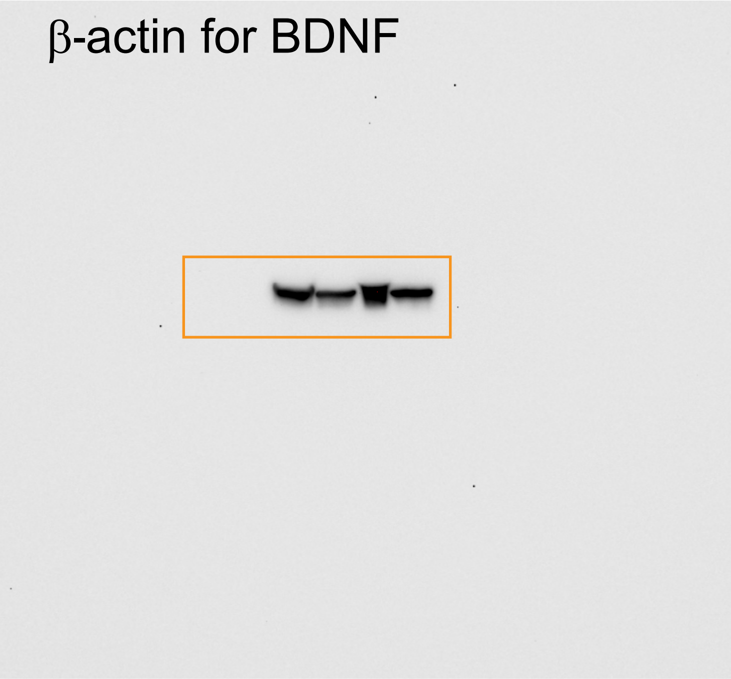

Extended Data Figure 3c

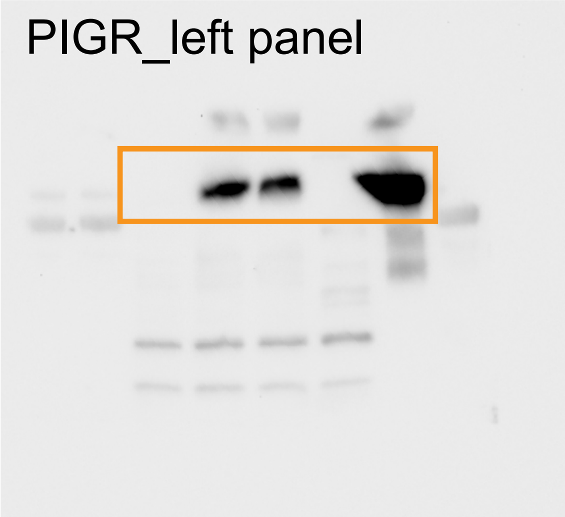

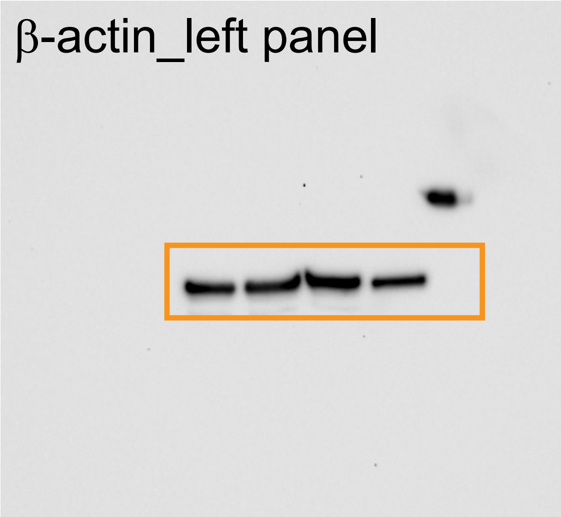

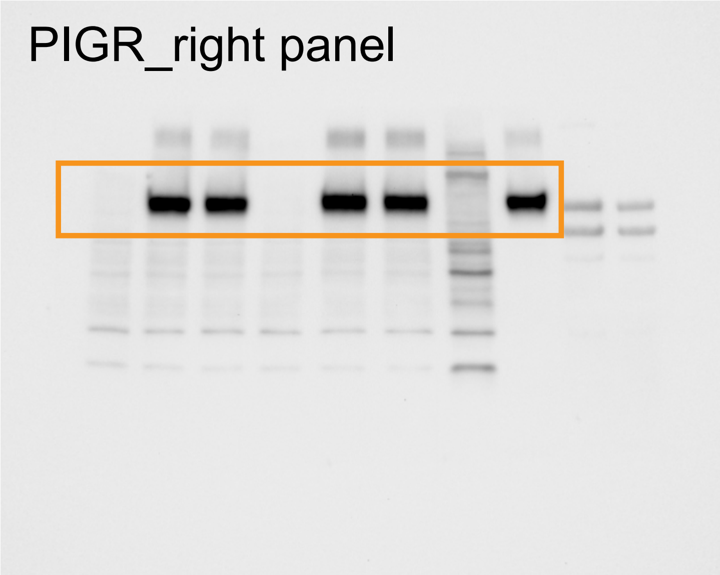

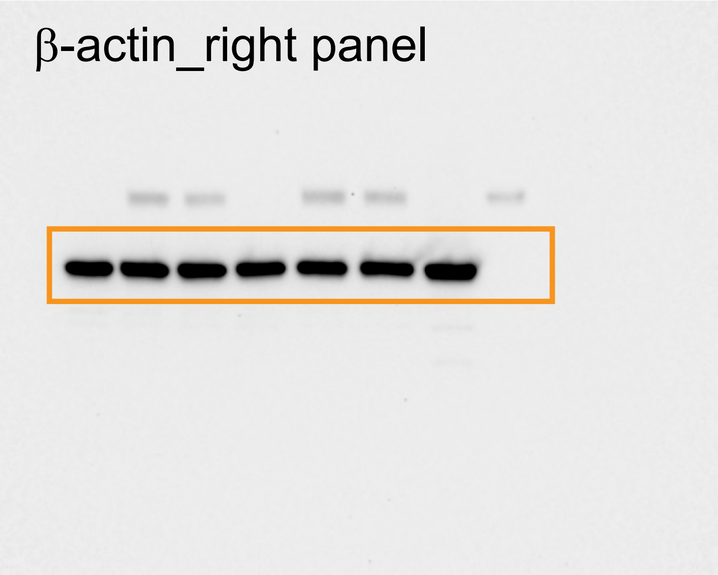

Extended Data Figure 4a

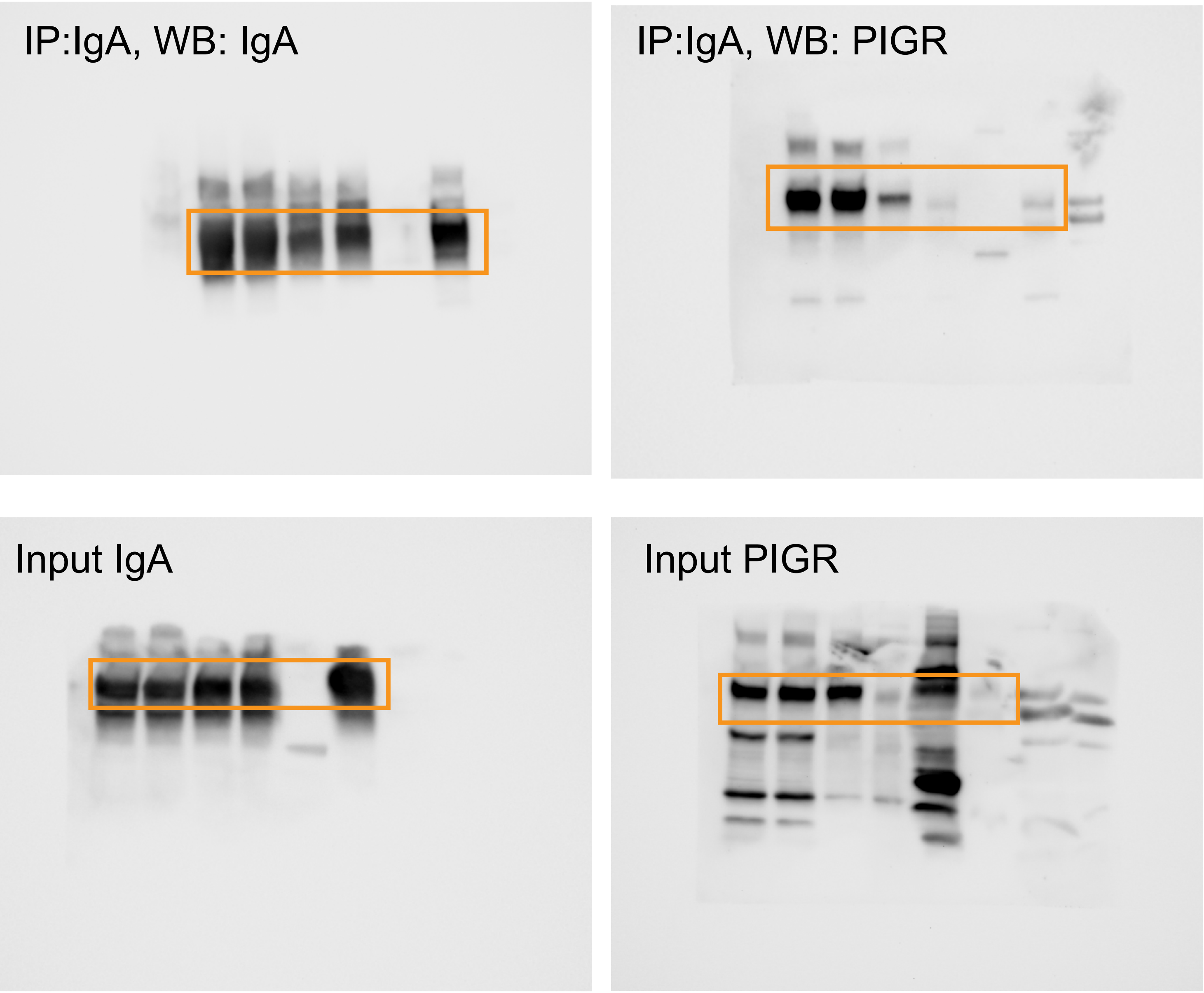

Extended Data Figure 4h

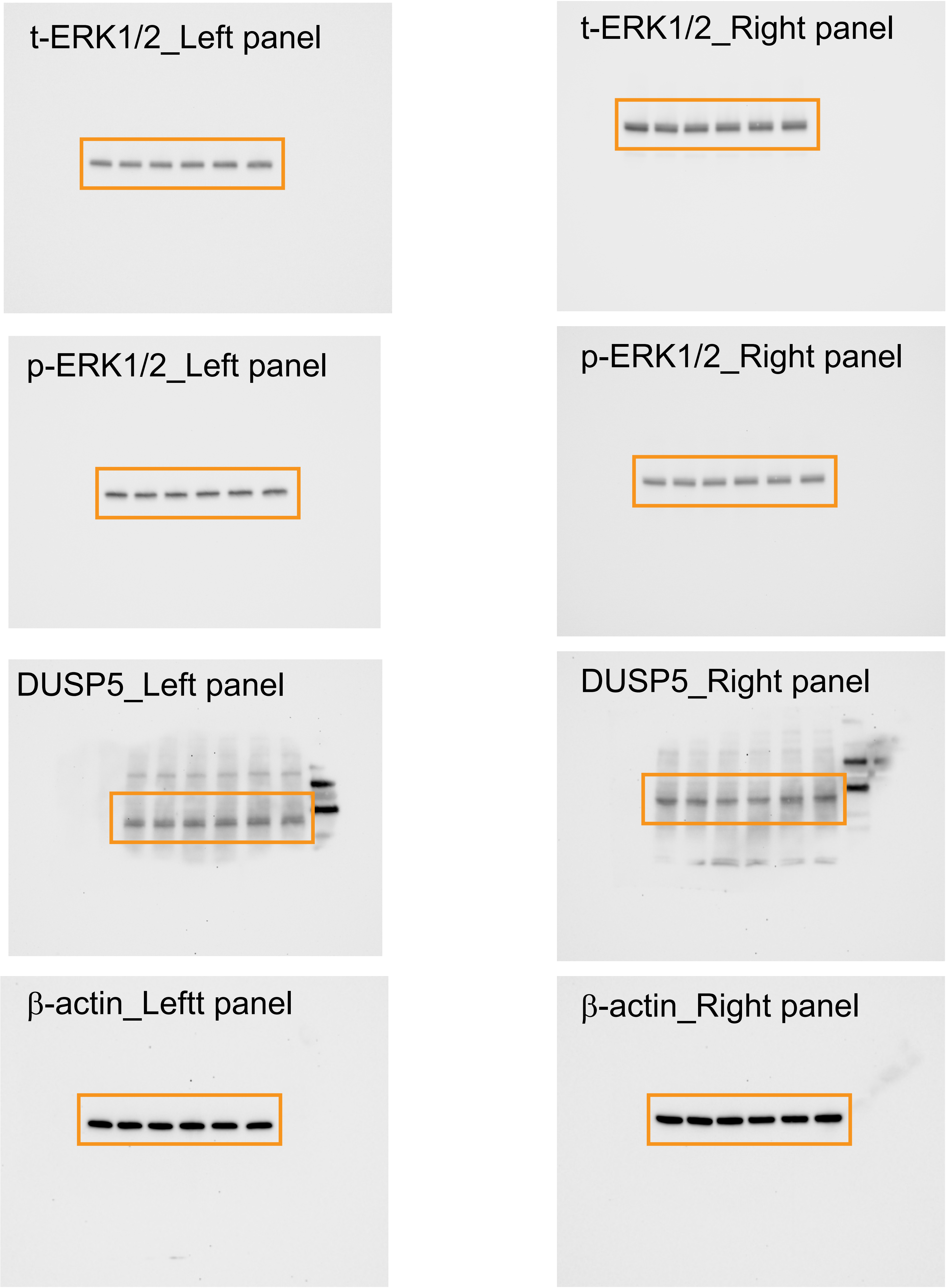

Supplementary Table 1

Statistical information associated with Figure 1a							
**Ig -class**	**Population**	**Min.**	**25th Percentile**	**Median**	**75th Percentile**	**Max.**	**IQR**
**IgA**	B cells	0.214285714	0.585060351	0.691489362	0.80126183	1	0.216201479
**IgA**	Plasma cells	0.333333333	0.402777778	0.493519442	0.644444444	0.909090909	0.241666667
**IgA**	Plasmablasts	0.289473684	0.42503639	0.537878788	0.666666667	1	0.241630277
**IgG**	B cells	0	0.011288805	0.031914894	0.075709779	0.333333333	0.064420974
**IgG**	Plasma cells	0.037220844	0.218181818	0.35413643	0.414755733	0.584541063	0.196573915
**IgG**	Plasmablasts	0	0.257575758	0.331491713	0.446969697	0.537425854	0.189393939
**IgM**	B cells	0	0.134241245	0.210526316	0.333333333	0.785714286	0.199092088
**IgM**	Plasma cells	0	0.076719577	0.130434783	0.2	0.397022333	0.123280423
**IgM**	Plasmablasts	0	0.051313436	0.102040816	0.166666667	0.243093923	0.115353231

Horizontal black lines with each box present median values; boxes extend from 25th to 75th percentile of values; whiskers extend to a maximum of 1.5 X IQR (interquartile range=75th percentile-25th percentile) beyond the boxes; dots denote the value of all observations for each box; the lowest dots are the minimum values and highest dots are the maximum values for each box.

**Comparison between**	***P* value**	**Test performed**
IgA *vs.* IgG	1e-10	Two-way ANOVA followed by Dunnett's ad hoc tests for multiple comparison was performed on arcsin-transformed percentage data
IgA *vs.* IgM	1e-10	

Supplementary Table 2

Statistical information associated with Figure 2a

Cell type	Treatment and time	Number of cells (*n*) quantified
WT OVCAR3	IgG, 0 min	319
WT OVCAR3	IgG, 15 min	290
WT OVCAR3	IgG, 1 hr	342
WT OVCAR3	IgG, 8 hr	366
WT OVCAR3	IgA, 0 min	348
WT OVCAR3	IgA, 15 min	287
WT OVCAR3	IgA, 1 hr	342
WT OVCAR3	IgA, 8 hr	278
WT OVCAR3	Pep-IgA, 0 min	212
WT OVCAR3	Pep-IgA, 15 min	307
WT OVCAR3	Pep-IgA, 1 hr	321
WT OVCAR3	Pep-IgA, 8 hr	278
PIGR^{CRSIPR} OVCAR3	IgA, 0 min	282
PIGR^{CRSIPR} OVCAR3	IgA, 15 min	260
PIGR^{CRSIPR} OVCAR3	IgA, 1 hr	313
PIGR^{CRSIPR} OVCAR3	IgA, 8 hr	304

Between treatment groups	*P* value	Test performed
WT OVCAR3 IgA_0 min *vs.* WT_OVACR3_IgA_15 min	<0.0001	Unpaired two-tailed t-test
WT_OVCAR3_IgA_15 min *vs.* WT_OVACR3_IgA_1 hr	<0.0001	Unpaired two-tailed t-test
WT_OVACR3_IgA_1 hr *vs.* WT_OVACR3_IgA_8 hr	<0.0001	Unpaired two-tailed t-test
WT_OVCAR3_IgG_8 hr *vs.* WT_OVACR3_IgA_8 hr	<0.0001	Unpaired two-tailed t-test
WT_OVCAR3_IgA_8 hr *vs.* WT_OVACR3_Pep-IgA_8 hr	<0.0001	Unpaired two-tailed t-test
WT_OVCAR3_IgA_8 hr *vs.* PIGR^{CRSIPR} _OVACR3_ IgA_8 hr	<0.0001	Unpaired two-tailed t-test

Statistical information associated with Figure 2f

Effector cell type	Target cell type	Target-effector ratio	*P* value	Test performed
NY-ESO-1-TCR-transduced T-cells	OVCAR3^{Luci}-NY-ESO-1	1:1	0.0127	ordinary one-way ANOVA
NY-ESO-1-TCR-transduced T-cells	OVCAR3^{Luci}-NY-ESO-1	1:10	0.0255	
NY-ESO-1-TCR-transduced T-cells	OVCAR3^{Luci}-NY-ESO-1	1:20	0.0063	
FSH-CER T-cells	OVCAR3^{Luci}	1:1	0.0248	
FSH-CER T-cells	OVCAR3^{Luci}	1:10	0.0006	
FSH-CER T-cells	OVCAR3^{Luci}	1:20	<0.0001	

Supplementary Table 3

Statistical information associated with Figure 3b

Growth curve statistics

Between treatment groups	*P* value	Test performed
Tumour#1: PBS *vs.* iIgA	0.0155	Paired two-tailed t-test
Tumour#1: iIgA *vs.* Ab1	0.0144	Paired two-tailed t-test
Tumour#2: PBS *vs.* iIgA	0.0175	Paired two-tailed t-test
Tumour#2: iIgA *vs.* Ab2	0.0044	Paired two-tailed t-test
Tumour#2: iIgA *vs.* Ab3	0.0061	Paired two-tailed t-test

Tumour weight statistics

Between treatment groups	*P* value	Test performed
Tumour#1: PBS *vs.* iIgA	<0.0001	Unpaired two-tailed t-test
Tumour#1: iIgA *vs.* Ab1	<0.0001	Unpaired two-tailed t-test
Tumour#2: PBS *vs.* iIgA	<0.0001	Unpaired two-tailed t-test
Tumour#2: iIgA *vs.* Ab2	<0.0001	Unpaired two-tailed t-test
Tumour#2: iIgA *vs.* Ab3	<0.0001	Unpaired two-tailed t-test

Supplementary Table 4

Statistical information associated with Figure 3d

Growth curve statistics

**Between treatment groups**	***P* value**	**Test performed**
iIgA *vs.* iIgG	0.0292	Paired two-tailed t-test
iIgA *vs.* αTSPAN7	0.0177	Paired two-tailed t-test
αTSPAN7 *vs.* αBDNF	0.0064	Paired two-tailed t-test

Tumour weight statistics

**Between treatment groups**	***P* value**	**Test performed**
iIgA *vs.* iIgG	0.0006	Unpaired two-tailed t-test
iIgA *vs.* αTSPAN7	0.0308	Unpaired two-tailed t-test
iIgA *vs.* αBDNF	0.0465	Unpaired two-tailed t-test

Supplementary Table 5

Statistical information associated with Figure 3f

Growth curve statistics

Between treatment groups	*P* value	Test performed
F(ab')$_2$-αTSPAN7 *vs.* F(ab')$_2$-αBDNF	0.0255	Paired two-tailed t-test
F(ab')$_2$-iIgA *vs.* F(ab')$_2$-iIgG	0.0563	Paired two-tailed t-test

Tumour weight statistics

Between treatment groups	*P* value	Test performed
F(ab')$_2$-αTSPAN7 *vs.* F(ab')$_2$-αBDNF	0.0030	Unpaired two-tailed t-test
F(ab')$_2$-iIgA *vs.* F(ab')$_2$-αBDNF	0.0006	Unpaired two-tailed t-test
F(ab')$_2$-iIgA *vs.* F(ab')$_2$-iIgG	0.6145	Unpaired two-tailed t-test
F(ab')$_2$-iIgA *vs.* F(ab')$_2$-αTSPAN7	0.3231	Unpaired two-tailed t-test

Supplementary Table 6

Statistical information associated with Figure 4a

Growth curve statistics

Between treatment groups	*P* value	Test performed
iIgA *vs.* PBS	0.0067	Paired two-tailed t-test
iIgA *vs.* αTSPAN7	0.1504	Paired two-tailed t-test

Tumour weight statistics

Between treatment groups	*P* value	Test performed
iIgA *vs.* PBS	<0.0001	Unpaired two-tailed t-test
iIgA *vs.* αTSPAN7	0.4336	Unpaired two-tailed t-test

Supplementary Table 7

Statistical information associated with Figure 4b

Growth curve statistics

Between treatment groups	*P* value	Test performed
iIgA *vs.* PBS	0.0197	Paired two-tailed t-test
iIgA *vs.* αTSPAN7	0.0140	Paired two-tailed t-test
iIgA *vs.* αTSPAN7+αNK1.1	0.0175	Paired two-tailed t-test

Tumour weight statistics

Between treatment groups	*P* value	Test performed
iIgA *vs.* PBS	<0.0001	Unpaired two-tailed t-test
iIgA *vs.* αTSPAN7	0.0004	Unpaired two-tailed t-test
αTSPAN7 *vs.* αTSPAN7+αNK1.1	0.9119	Unpaired two-tailed t-test

Supplementary Table 8

Statistical information associated with Figure 4d

**Between treatment groups**	***P* value**	**Test performed**
Uncoated:with-αCD351^{Neut} *vs.* Uncoated:no-αCD351^{Neut}	0.8115	Unpaired two-tailed t-test
αTSPAN7^{coated}:with-αCD351^{Neut} *vs.* αTSPAN7^{coated}:no-αCD351^{Neut}	0.0244	Unpaired two-tailed t-test

Statistical information associated with Figure 4e

**Between treatment groups**	***P* value**	**Test performed**
PBS *vs*. iIgA	0.0036	Unpaired two-tailed t-test
iIgA *vs.* αTSPAN7	0.0132	Unpaired two-tailed t-test
αTSPAN7 *vs.* αTSPAN7+αNK1.1	0.6264	Unpaired two-tailed t-test

Supplementary Table 9

Statistical information associated with Figure 4f

Growth curve statistics

Between treatment groups	*P* value	Test performed
WT:iIgA *vs.* WT:PBS	0.0068	Paired two-tailed t-test
WT:iIgA *vs.* CR:IgA	0.0015	Paired two-tailed t-test
WT:αBDNF *vs.* CR:αBDNF	0.0107	Paired two-tailed t-test
WT:αTSPAN7 *vs.* CR:αTSPAN7	0.0080	Paired two-tailed t-test
WT:PBS *vs.* CR:PBS	0.1143	Paired two-tailed t-test
WT:PBS *vs.* CR:iIgA	0.9062	Paired two-tailed t-test

Tumour weight statistics

Between treatment groups	*P* value	Test performed
WT:iIgA *vs.* CR:iIgA	0.0266	Unpaired two-tailed t-test
WT:αBDNF *vs.* CR:αBDNF	0.7354	Unpaired two-tailed t-test
WT:αTSPAN7 *vs.* CR:αTSPAN7	0.0009	Unpaired two-tailed t-test
WT:PBS *vs.* CR:PBS	0.8425	Unpaired two-tailed t-test
CR:PBS *vs.* CR:iIgA	0.8637	Unpaired two-tailed t-test

Supplementary Table 10

Patient Derived specimens			
**Specimen type**	**ID**	**Experiment**	**Description**
Solid tumours	S1	FACS, B cell immortalization	High grade Serous Ovarian Cancer
Solid tumours	S2	FACS, B cell immortalization	High grade Serous Ovarian Cancer
Solid tumours	S3	FACS	High grade Serous Ovarian Cancer
Solid tumours	S4	FACS	High grade Serous Ovarian Cancer
Solid tumours	S5	FACS, B cell immortalization	High grade Serous Ovarian Cancer
Solid tumours	S6	FACS	High grade Serous Ovarian Cancer
Solid tumours	S7	FACS, B cell immortalization	High grade Serous Ovarian Cancer
Solid tumours	S8	FACS	High grade Serous Ovarian Cancer
Solid tumours	S9	FACS	High grade Serous Ovarian Cancer
Solid tumours	S10	FACS, BCR sequencing	High grade Serous Ovarian Cancer
Solid tumours	S11	FACS, B cell immortalization	High grade Serous Ovarian Cancer
Solid tumours	S12	FACS	High grade Serous Ovarian Cancer
Solid tumours	S13	FACS	High grade Serous Ovarian Cancer
Solid tumours	S14	FACS, B cell immortalization	High grade Serous Ovarian Cancer
Solid tumours	S15	FACS, B cell immortalization	High grade Serous Ovarian Cancer
Solid tumours	S16	FACS	High grade Serous Ovarian Cancer
Solid tumours	S17	FACS	High grade Serous Ovarian Cancer
Solid tumours	S18	FACS, B cell immortalization	High grade Serous Ovarian Cancer
Solid tumours	S19	FACS	High grade Serous Ovarian Cancer
Solid tumours	S20	FACS	High grade Serous Ovarian Cancer
Solid tumours	S21	FACS, B cell immortalization	High grade Serous Ovarian Cancer
Solid tumours	S22	FACS, B cell immortalization	High grade Serous Ovarian Cancer
Solid tumours	S23	FACS	High grade Serous Ovarian Cancer
Solid tumours	S24	FACS	High grade Serous Ovarian Cancer
Solid tumours	S25	FACS	High grade Serous Ovarian Cancer
Solid tumours	S26	FACS	High grade Serous Ovarian Cancer
Solid tumours	S27	FACS	High grade Serous Ovarian Cancer
Solid tumours	S28	FACS	High grade Serous Ovarian Cancer
Solid tumours	S29	FACS, BCR sequencing	High grade Serous Ovarian Cancer
Ascites	A1	Co-Immunoprecipitation	High grade Serous Ovarian Cancer
Ascites	A2	Co-Immunoprecipitation	High grade Serous Ovarian Cancer
FFPE-TMA	MCC1	Multiplex Immunofluorescence	High grade Serous Ovarian Cancer
FFPE-TMA	MCC2	Multiplex Immunofluorescence	High grade Serous Ovarian Cancer
FFPE-TMA	MCC3	Multiplex Immunofluorescence	High grade Serous Ovarian Cancer
FFPE-TMA	MCC4	Multiplex Immunofluorescence	High grade Serous Ovarian Cancer
FFPE-TMA	MCC5	Multiplex Immunofluorescence	High grade Serous Ovarian Cancer
FFPE-TMA	MCC6	Multiplex Immunofluorescence	High grade Serous Ovarian Cancer
FFPE-TMA	MCC7	Multiplex Immunofluorescence	High grade Serous Ovarian Cancer
FFPE-TMA	MCC8	Multiplex Immunofluorescence	High grade Serous Ovarian Cancer
FFPE-TMA	MCC9	Multiplex Immunofluorescence	High grade Serous Ovarian Cancer
FFPE-TMA	MCC10	Multiplex Immunofluorescence	High grade Serous Ovarian Cancer
FFPE-TMA	MCC11	Multiplex Immunofluorescence	High grade Serous Ovarian Cancer
FFPE-TMA	MCC12	Multiplex Immunofluorescence	High grade Serous Ovarian Cancer
FFPE-TMA	MCC13	Multiplex Immunofluorescence	High grade Serous Ovarian Cancer
FFPE-TMA	MCC14	Multiplex Immunofluorescence	High grade Serous Ovarian Cancer
FFPE-TMA	MCC15	Multiplex Immunofluorescence	High grade Serous Ovarian Cancer

FFPE-TMA	MCC16	Multiplex Immunofluorescence	High grade Serous Ovarian Cancer
FFPE-TMA	MCC17	Multiplex Immunofluorescence	High grade Serous Ovarian Cancer
FFPE-TMA	MCC18	Multiplex Immunofluorescence	High grade Serous Ovarian Cancer
FFPE-TMA	MCC19	Multiplex Immunofluorescence	High grade Serous Ovarian Cancer
FFPE-TMA	MCC20	Multiplex Immunofluorescence	High grade Serous Ovarian Cancer
FFPE-TMA	MCC21	Multiplex Immunofluorescence	High grade Serous Ovarian Cancer
FFPE-TMA	MCC22	Multiplex Immunofluorescence	High grade Serous Ovarian Cancer
FFPE-TMA	MCC23	Multiplex Immunofluorescence	High grade Serous Ovarian Cancer
FFPE-TMA	MCC24	Multiplex Immunofluorescence	High grade Serous Ovarian Cancer
FFPE-TMA	MCC25	Multiplex Immunofluorescence	High grade Serous Ovarian Cancer
FFPE-TMA	MCC26	Multiplex Immunofluorescence	High grade Serous Ovarian Cancer
FFPE-TMA	MCC27	Multiplex Immunofluorescence	High grade Serous Ovarian Cancer
FFPE-TMA	MCC28	Multiplex Immunofluorescence	High grade Serous Ovarian Cancer
FFPE-TMA	MCC29	Multiplex Immunofluorescence	High grade Serous Ovarian Cancer
FFPE-TMA	MCC30	Multiplex Immunofluorescence	High grade Serous Ovarian Cancer
FFPE-TMA	MCC31	Multiplex Immunofluorescence	High grade Serous Ovarian Cancer
FFPE-TMA	MCC32	Multiplex Immunofluorescence	High grade Serous Ovarian Cancer
FFPE-TMA	MCC33	Multiplex Immunofluorescence	High grade Serous Ovarian Cancer
FFPE-TMA	MCC34	Multiplex Immunofluorescence	High grade Serous Ovarian Cancer
FFPE-TMA	MCC35	Multiplex Immunofluorescence	High grade Serous Ovarian Cancer
FFPE-TMA	MCC36	Multiplex Immunofluorescence	High grade Serous Ovarian Cancer
FFPE-TMA	MCC37	Multiplex Immunofluorescence	High grade Serous Ovarian Cancer
FFPE-TMA	MCC38	Multiplex Immunofluorescence	High grade Serous Ovarian Cancer
FFPE-TMA	MCC39	Multiplex Immunofluorescence	High grade Serous Ovarian Cancer
FFPE-TMA	MCC40	Multiplex Immunofluorescence	High grade Serous Ovarian Cancer
FFPE-TMA	MCC41	Multiplex Immunofluorescence	High grade Serous Ovarian Cancer
FFPE-TMA	MCC42	Multiplex Immunofluorescence	High grade Serous Ovarian Cancer
FFPE-TMA	MCC43	Multiplex Immunofluorescence	High grade Serous Ovarian Cancer
FFPE-TMA	MCC44	Multiplex Immunofluorescence	High grade Serous Ovarian Cancer
FFPE-TMA	MCC45	Multiplex Immunofluorescence	High grade Serous Ovarian Cancer
FFPE-TMA	MCC46	Multiplex Immunofluorescence	High grade Serous Ovarian Cancer
FFPE-TMA	MCC47	Multiplex Immunofluorescence	High grade Serous Ovarian Cancer
FFPE-TMA	MCC48	Multiplex Immunofluorescence	High grade Serous Ovarian Cancer
FFPE-TMA	MCC49	Multiplex Immunofluorescence	High grade Serous Ovarian Cancer
FFPE-TMA	MCC50	Multiplex Immunofluorescence	High grade Serous Ovarian Cancer
FFPE-TMA	MCC51	Multiplex Immunofluorescence	High grade Serous Ovarian Cancer
FFPE-TMA	MCC52	Multiplex Immunofluorescence	High grade Serous Ovarian Cancer
FFPE-TMA	MCC53	Multiplex Immunofluorescence	High grade Serous Ovarian Cancer
FFPE-TMA	MCC54	Multiplex Immunofluorescence	High grade Serous Ovarian Cancer
FFPE-TMA	MCC55	Multiplex Immunofluorescence	High grade Serous Ovarian Cancer
FFPE-TMA	MCC56	Multiplex Immunofluorescence	High grade Serous Ovarian Cancer
FFPE-TMA	MCC57	Multiplex Immunofluorescence	High grade Serous Ovarian Cancer
FFPE-TMA	MCC58	Multiplex Immunofluorescence	High grade Serous Ovarian Cancer
FFPE-TMA	MCC59	Multiplex Immunofluorescence	High grade Serous Ovarian Cancer
FFPE-TMA	MCC60	Multiplex Immunofluorescence	High grade Serous Ovarian Cancer
FFPE-TMA	MCC61	Multiplex Immunofluorescence	High grade Serous Ovarian Cancer
FFPE-TMA	MCC62	Multiplex Immunofluorescence	High grade Serous Ovarian Cancer
FFPE-TMA	MCC63	Multiplex Immunofluorescence	High grade Serous Ovarian Cancer
FFPE-TMA	MCC64	Multiplex Immunofluorescence	High grade Serous Ovarian Cancer
FFPE-TMA	MCC65	Multiplex Immunofluorescence	High grade Serous Ovarian Cancer
FFPE-TMA	MCC66	Multiplex Immunofluorescence	High grade Serous Ovarian Cancer
FFPE-TMA	MCC67	Multiplex Immunofluorescence	High grade Serous Ovarian Cancer
FFPE-TMA	MCC68	Multiplex Immunofluorescence	High grade Serous Ovarian Cancer

FFPE-TMA	MCC69	Multiplex Immunofluorescence	High grade Serous Ovarian Cancer
FFPE-TMA	MCC70	Multiplex Immunofluorescence	High grade Serous Ovarian Cancer
FFPE-TMA	MCC71	Multiplex Immunofluorescence	High grade Serous Ovarian Cancer
FFPE-TMA	MCC72	Multiplex Immunofluorescence	High grade Serous Ovarian Cancer
FFPE-TMA	MCC73	Multiplex Immunofluorescence	High grade Serous Ovarian Cancer
FFPE-TMA	MCC74	Multiplex Immunofluorescence	High grade Serous Ovarian Cancer
FFPE-TMA	MCC75	Multiplex Immunofluorescence	High grade Serous Ovarian Cancer
FFPE-TMA	MCC76	Multiplex Immunofluorescence	High grade Serous Ovarian Cancer
FFPE-TMA	MCC77	Multiplex Immunofluorescence	High grade Serous Ovarian Cancer
FFPE-TMA	MCC78	Multiplex Immunofluorescence	High grade Serous Ovarian Cancer
FFPE-TMA	MCC79	Multiplex Immunofluorescence	High grade Serous Ovarian Cancer
FFPE-TMA	MCC80	Multiplex Immunofluorescence	High grade Serous Ovarian Cancer
FFPE-TMA	MCC81	Multiplex Immunofluorescence	High grade Serous Ovarian Cancer
FFPE-TMA	MCC82	Multiplex Immunofluorescence	High grade Serous Ovarian Cancer
FFPE-TMA	MCC83	Multiplex Immunofluorescence	High grade Serous Ovarian Cancer
FFPE-TMA	MCC84	Multiplex Immunofluorescence	High grade Serous Ovarian Cancer
FFPE-TMA	MCC85	Multiplex Immunofluorescence	High grade Serous Ovarian Cancer
FFPE-TMA	MCC86	Multiplex Immunofluorescence	High grade Serous Ovarian Cancer
FFPE-TMA	MCC87	Multiplex Immunofluorescence	High grade Serous Ovarian Cancer
FFPE-TMA	MCC88	Multiplex Immunofluorescence	High grade Serous Ovarian Cancer
FFPE-TMA	MCC89	Multiplex Immunofluorescence	High grade Serous Ovarian Cancer
FFPE-TMA	MCC90	Multiplex Immunofluorescence	High grade Serous Ovarian Cancer
FFPE-TMA	MCC91	Multiplex Immunofluorescence	High grade Serous Ovarian Cancer
FFPE-TMA	MCC92	Multiplex Immunofluorescence	High grade Serous Ovarian Cancer
FFPE-TMA	MCC93	Multiplex Immunofluorescence	High grade Serous Ovarian Cancer
FFPE-TMA	NECC1	Multiplex Immunofluorescence	High grade Serous Ovarian Cancer
FFPE-TMA	NECC2	Multiplex Immunofluorescence	High grade Serous Ovarian Cancer
FFPE-TMA	NECC3	Multiplex Immunofluorescence	High grade Serous Ovarian Cancer
FFPE-TMA	NECC4	Multiplex Immunofluorescence	High grade Serous Ovarian Cancer
FFPE-TMA	NECC5	Multiplex Immunofluorescence	High grade Serous Ovarian Cancer
FFPE-TMA	NECC6	Multiplex Immunofluorescence	High grade Serous Ovarian Cancer
FFPE-TMA	NECC7	Multiplex Immunofluorescence	High grade Serous Ovarian Cancer
FFPE-TMA	NECC8	Multiplex Immunofluorescence	High grade Serous Ovarian Cancer
FFPE-TMA	NECC9	Multiplex Immunofluorescence	High grade Serous Ovarian Cancer
FFPE-TMA	NECC10	Multiplex Immunofluorescence	High grade Serous Ovarian Cancer
FFPE-TMA	NECC11	Multiplex Immunofluorescence	High grade Serous Ovarian Cancer
FFPE-TMA	NECC12	Multiplex Immunofluorescence	High grade Serous Ovarian Cancer
FFPE-TMA	NECC13	Multiplex Immunofluorescence	High grade Serous Ovarian Cancer
FFPE-TMA	NECC14	Multiplex Immunofluorescence	High grade Serous Ovarian Cancer
FFPE-TMA	NECC15	Multiplex Immunofluorescence	High grade Serous Ovarian Cancer
FFPE-TMA	NECC16	Multiplex Immunofluorescence	High grade Serous Ovarian Cancer
FFPE-TMA	NECC17	Multiplex Immunofluorescence	High grade Serous Ovarian Cancer
FFPE-TMA	NECC18	Multiplex Immunofluorescence	High grade Serous Ovarian Cancer
FFPE-TMA	NECC19	Multiplex Immunofluorescence	High grade Serous Ovarian Cancer
FFPE-TMA	NECC20	Multiplex Immunofluorescence	High grade Serous Ovarian Cancer
FFPE-TMA	NECC21	Multiplex Immunofluorescence	High grade Serous Ovarian Cancer
FFPE-TMA	NECC22	Multiplex Immunofluorescence	High grade Serous Ovarian Cancer
FFPE-TMA	NECC23	Multiplex Immunofluorescence	High grade Serous Ovarian Cancer
FFPE-TMA	NECC24	Multiplex Immunofluorescence	High grade Serous Ovarian Cancer
FFPE-TMA	NECC25	Multiplex Immunofluorescence	High grade Serous Ovarian Cancer
FFPE-TMA	NECC26	Multiplex Immunofluorescence	High grade Serous Ovarian Cancer
FFPE-TMA	NECC27	Multiplex Immunofluorescence	High grade Serous Ovarian Cancer
FFPE-TMA	NECC28	Multiplex Immunofluorescence	High grade Serous Ovarian Cancer

FFPE-TMA	NECC29	Multiplex Immunofluorescence	High grade Serous Ovarian Cancer
FFPE-TMA	NECC30	Multiplex Immunofluorescence	High grade Serous Ovarian Cancer
FFPE-TMA	NECC31	Multiplex Immunofluorescence	High grade Serous Ovarian Cancer
FFPE-TMA	NECC32	Multiplex Immunofluorescence	High grade Serous Ovarian Cancer
FFPE-TMA	NECC33	Multiplex Immunofluorescence	High grade Serous Ovarian Cancer
FFPE-TMA	NECC34	Multiplex Immunofluorescence	High grade Serous Ovarian Cancer
FFPE-TMA	NECC35	Multiplex Immunofluorescence	High grade Serous Ovarian Cancer
FFPE-TMA	NECC36	Multiplex Immunofluorescence	High grade Serous Ovarian Cancer
FFPE-TMA	NECC37	Multiplex Immunofluorescence	High grade Serous Ovarian Cancer
FFPE-TMA	NECC38	Multiplex Immunofluorescence	High grade Serous Ovarian Cancer
FFPE-TMA	NECC39	Multiplex Immunofluorescence	High grade Serous Ovarian Cancer
FFPE-TMA	NECC40	Multiplex Immunofluorescence	High grade Serous Ovarian Cancer
FFPE-TMA	NECC41	Multiplex Immunofluorescence	High grade Serous Ovarian Cancer
FFPE-TMA	NECC42	Multiplex Immunofluorescence	High grade Serous Ovarian Cancer
FFPE-TMA	NECC43	Multiplex Immunofluorescence	High grade Serous Ovarian Cancer
FFPE-TMA	NECC44	Multiplex Immunofluorescence	High grade Serous Ovarian Cancer
FFPE-TMA	NECC45	Multiplex Immunofluorescence	High grade Serous Ovarian Cancer
FFPE-TMA	NECC46	Multiplex Immunofluorescence	High grade Serous Ovarian Cancer
FFPE-TMA	NECC47	Multiplex Immunofluorescence	High grade Serous Ovarian Cancer
FFPE-TMA	NECC48	Multiplex Immunofluorescence	High grade Serous Ovarian Cancer
FFPE-TMA	NECC49	Multiplex Immunofluorescence	High grade Serous Ovarian Cancer
FFPE-TMA	NECC50	Multiplex Immunofluorescence	High grade Serous Ovarian Cancer
FFPE-TMA	NECC51	Multiplex Immunofluorescence	High grade Serous Ovarian Cancer
FFPE-TMA	NECC52	Multiplex Immunofluorescence	High grade Serous Ovarian Cancer
FFPE-TMA	NECC53	Multiplex Immunofluorescence	High grade Serous Ovarian Cancer
FFPE-TMA	NECC54	Multiplex Immunofluorescence	High grade Serous Ovarian Cancer
FFPE-TMA	NECC55	Multiplex Immunofluorescence	High grade Serous Ovarian Cancer
FFPE-TMA	NECC56	Multiplex Immunofluorescence	High grade Serous Ovarian Cancer
FFPE-TMA	NECC57	Multiplex Immunofluorescence	High grade Serous Ovarian Cancer
FFPE-TMA	NECC58	Multiplex Immunofluorescence	High grade Serous Ovarian Cancer
FFPE-TMA	NECC59	Multiplex Immunofluorescence	High grade Serous Ovarian Cancer
FFPE-TMA	NECC60	Multiplex Immunofluorescence	High grade Serous Ovarian Cancer
FFPE-TMA	NECC61	Multiplex Immunofluorescence	High grade Serous Ovarian Cancer
FFPE-TMA	NECC62	Multiplex Immunofluorescence	High grade Serous Ovarian Cancer
FFPE-TMA	NECC63	Multiplex Immunofluorescence	High grade Serous Ovarian Cancer
FFPE-TMA	NECC64	Multiplex Immunofluorescence	High grade Serous Ovarian Cancer
FFPE-TMA	NECC65	Multiplex Immunofluorescence	High grade Serous Ovarian Cancer
FFPE-TMA	NECC66	Multiplex Immunofluorescence	High grade Serous Ovarian Cancer
FFPE-TMA	NECC67	Multiplex Immunofluorescence	High grade Serous Ovarian Cancer
FFPE-TMA	NECC68	Multiplex Immunofluorescence	High grade Serous Ovarian Cancer
FFPE-TMA	NECC69	Multiplex Immunofluorescence	High grade Serous Ovarian Cancer
FFPE-TMA	NECC70	Multiplex Immunofluorescence	High grade Serous Ovarian Cancer
FFPE-TMA	NECC71	Multiplex Immunofluorescence	High grade Serous Ovarian Cancer
FFPE-TMA	NECC72	Multiplex Immunofluorescence	High grade Serous Ovarian Cancer
FFPE-TMA	NECC73	Multiplex Immunofluorescence	High grade Serous Ovarian Cancer
FFPE-TMA	NECC74	Multiplex Immunofluorescence	High grade Serous Ovarian Cancer
FFPE-TMA	NECC75	Multiplex Immunofluorescence	High grade Serous Ovarian Cancer
FFPE-TMA	NECC76	Multiplex Immunofluorescence	High grade Serous Ovarian Cancer
FFPE-TMA	NECC77	Multiplex Immunofluorescence	High grade Serous Ovarian Cancer
FFPE-TMA	NECC78	Multiplex Immunofluorescence	High grade Serous Ovarian Cancer
FFPE-TMA	NECC79	Multiplex Immunofluorescence	High grade Serous Ovarian Cancer
FFPE-TMA	NECC80	Multiplex Immunofluorescence	High grade Serous Ovarian Cancer
FFPE-TMA	NECC81	Multiplex Immunofluorescence	High grade Serous Ovarian Cancer

FFPE-TMA	NECC82	Multiplex Immunofluorescence	High grade Serous Ovarian Cancer
FFPE-TMA	NECC83	Multiplex Immunofluorescence	High grade Serous Ovarian Cancer
FFPE-TMA	NECC84	Multiplex Immunofluorescence	High grade Serous Ovarian Cancer
FFPE-TMA	NECC85	Multiplex Immunofluorescence	High grade Serous Ovarian Cancer
FFPE-TMA	NECC86	Multiplex Immunofluorescence	High grade Serous Ovarian Cancer
FFPE-TMA	NECC87	Multiplex Immunofluorescence	High grade Serous Ovarian Cancer
FFPE-TMA	NECC88	Multiplex Immunofluorescence	High grade Serous Ovarian Cancer
FFPE-TMA	NECC89	Multiplex Immunofluorescence	High grade Serous Ovarian Cancer
FFPE-TMA	NECC90	Multiplex Immunofluorescence	High grade Serous Ovarian Cancer
FFPE-TMA	NECC91	Multiplex Immunofluorescence	High grade Serous Ovarian Cancer
FFPE-TMA	NECC92	Multiplex Immunofluorescence	High grade Serous Ovarian Cancer
FFPE-TMA	NECC93	Multiplex Immunofluorescence	High grade Serous Ovarian Cancer
FFPE-TMA	NECC94	Multiplex Immunofluorescence	High grade Serous Ovarian Cancer
FFPE-TMA	NECC95	Multiplex Immunofluorescence	High grade Serous Ovarian Cancer
FFPE-TMA	NECC96	Multiplex Immunofluorescence	High grade Serous Ovarian Cancer
FFPE-TMA	NECC97	Multiplex Immunofluorescence	High grade Serous Ovarian Cancer
FFPE-TMA	NECC98	Multiplex Immunofluorescence	High grade Serous Ovarian Cancer
FFPE-TMA	NECC99	Multiplex Immunofluorescence	High grade Serous Ovarian Cancer
FFPE-TMA	NECC100	Multiplex Immunofluorescence	High grade Serous Ovarian Cancer
FFPE-TMA	NECC101	Multiplex Immunofluorescence	High grade Serous Ovarian Cancer
FFPE-TMA	NECC102	Multiplex Immunofluorescence	High grade Serous Ovarian Cancer
FFPE-TMA	NECC103	Multiplex Immunofluorescence	High grade Serous Ovarian Cancer
FFPE-TMA	NECC104	Multiplex Immunofluorescence	High grade Serous Ovarian Cancer
FFPE-TMA	NECC105	Multiplex Immunofluorescence	High grade Serous Ovarian Cancer
FFPE-TMA	NECC106	Multiplex Immunofluorescence	High grade Serous Ovarian Cancer
FFPE-TMA	NECC107	Multiplex Immunofluorescence	High grade Serous Ovarian Cancer
FFPE-TMA	NECC108	Multiplex Immunofluorescence	High grade Serous Ovarian Cancer
FFPE-TMA	NECC109	Multiplex Immunofluorescence	High grade Serous Ovarian Cancer
FFPE-TMA	NECC110	Multiplex Immunofluorescence	High grade Serous Ovarian Cancer
FFPE-TMA	NECC111	Multiplex Immunofluorescence	High grade Serous Ovarian Cancer
FFPE-TMA	NECC112	Multiplex Immunofluorescence	High grade Serous Ovarian Cancer
FFPE-TMA	NECC113	Multiplex Immunofluorescence	High grade Serous Ovarian Cancer
FFPE-TMA	NECC114	Multiplex Immunofluorescence	High grade Serous Ovarian Cancer
FFPE-TMA	NECC115	Multiplex Immunofluorescence	High grade Serous Ovarian Cancer
FFPE-TMA	NECC116	Multiplex Immunofluorescence	High grade Serous Ovarian Cancer
FFPE-TMA	NECC117	Multiplex Immunofluorescence	High grade Serous Ovarian Cancer
FFPE-TMA	NECC118	Multiplex Immunofluorescence	High grade Serous Ovarian Cancer
FFPE-TMA	NECC119	Multiplex Immunofluorescence	High grade Serous Ovarian Cancer
FFPE-TMA	NECC120	Multiplex Immunofluorescence	High grade Serous Ovarian Cancer
FFPE-TMA	NECC121	Multiplex Immunofluorescence	High grade Serous Ovarian Cancer
FFPE-TMA	NECC122	Multiplex Immunofluorescence	High grade Serous Ovarian Cancer
FFPE-TMA	NECC123	Multiplex Immunofluorescence	High grade Serous Ovarian Cancer
FFPE-TMA	NECC124	Multiplex Immunofluorescence	High grade Serous Ovarian Cancer
FFPE-TMA	NECC125	Multiplex Immunofluorescence	High grade Serous Ovarian Cancer
FFPE-TMA	NECC126	Multiplex Immunofluorescence	High grade Serous Ovarian Cancer
FFPE-TMA	NECC127	Multiplex Immunofluorescence	High grade Serous Ovarian Cancer
FFPE-TMA	NECC128	Multiplex Immunofluorescence	High grade Serous Ovarian Cancer
FFPE-TMA	NECC129	Multiplex Immunofluorescence	High grade Serous Ovarian Cancer
FFPE-TMA	NECC130	Multiplex Immunofluorescence	High grade Serous Ovarian Cancer
FFPE-TMA	NECC131	Multiplex Immunofluorescence	High grade Serous Ovarian Cancer
FFPE-TMA	NECC132	Multiplex Immunofluorescence	High grade Serous Ovarian Cancer
FFPE-TMA	NECC133	Multiplex Immunofluorescence	High grade Serous Ovarian Cancer
FFPE-TMA	NECC134	Multiplex Immunofluorescence	High grade Serous Ovarian Cancer

FFPE-TMA	NECC135	Multiplex Immunofluorescence	High grade Serous Ovarian Cancer
FFPE-TMA	NECC136	Multiplex Immunofluorescence	High grade Serous Ovarian Cancer
FFPE-TMA	NECC137	Multiplex Immunofluorescence	High grade Serous Ovarian Cancer
FFPE-TMA	NECC138	Multiplex Immunofluorescence	High grade Serous Ovarian Cancer
FFPE-TMA	NECC139	Multiplex Immunofluorescence	High grade Serous Ovarian Cancer
FFPE-TMA	NECC140	Multiplex Immunofluorescence	High grade Serous Ovarian Cancer
FFPE-TMA	NECC141	Multiplex Immunofluorescence	High grade Serous Ovarian Cancer
FFPE-TMA	NECC142	Multiplex Immunofluorescence	High grade Serous Ovarian Cancer
FFPE-TMA	NECC143	Multiplex Immunofluorescence	High grade Serous Ovarian Cancer
FFPE-TMA	NECC144	Multiplex Immunofluorescence	High grade Serous Ovarian Cancer
FFPE-TMA	NECC145	Multiplex Immunofluorescence	High grade Serous Ovarian Cancer
FFPE-TMA	NECC146	Multiplex Immunofluorescence	High grade Serous Ovarian Cancer
FFPE-TMA	NECC147	Multiplex Immunofluorescence	High grade Serous Ovarian Cancer
FFPE-TMA	NECC148	Multiplex Immunofluorescence	High grade Serous Ovarian Cancer
FFPE-TMA	NECC149	Multiplex Immunofluorescence	High grade Serous Ovarian Cancer
FFPE-TMA	NECC150	Multiplex Immunofluorescence	High grade Serous Ovarian Cancer
FFPE-TMA	NECC151	Multiplex Immunofluorescence	High grade Serous Ovarian Cancer
FFPE-TMA	NECC152	Multiplex Immunofluorescence	High grade Serous Ovarian Cancer
FFPE-TMA	NECC153	Multiplex Immunofluorescence	High grade Serous Ovarian Cancer
FFPE-TMA	NECC154	Multiplex Immunofluorescence	High grade Serous Ovarian Cancer
FFPE-TMA	NECC155	Multiplex Immunofluorescence	High grade Serous Ovarian Cancer
FFPE-TMA	NECC156	Multiplex Immunofluorescence	High grade Serous Ovarian Cancer
FFPE-TMA	NECC157	Multiplex Immunofluorescence	High grade Serous Ovarian Cancer
FFPE-TMA	NECC158	Multiplex Immunofluorescence	High grade Serous Ovarian Cancer
FFPE-TMA	NECC159	Multiplex Immunofluorescence	High grade Serous Ovarian Cancer
FFPE-TMA	NECC160	Multiplex Immunofluorescence	High grade Serous Ovarian Cancer
FFPE-TMA	NECC161	Multiplex Immunofluorescence	High grade Serous Ovarian Cancer
FFPE-TMA	NECC162	Multiplex Immunofluorescence	High grade Serous Ovarian Cancer
FFPE-TMA	NECC163	Multiplex Immunofluorescence	High grade Serous Ovarian Cancer
FFPE-TMA	NECC164	Multiplex Immunofluorescence	High grade Serous Ovarian Cancer
FFPE-TMA	NECC165	Multiplex Immunofluorescence	High grade Serous Ovarian Cancer
FFPE-TMA	NECC166	Multiplex Immunofluorescence	High grade Serous Ovarian Cancer
FFPE-TMA	NECC167	Multiplex Immunofluorescence	High grade Serous Ovarian Cancer
FFPE-TMA	NECC168	Multiplex Immunofluorescence	High grade Serous Ovarian Cancer
FFPE-TMA	NECC169	Multiplex Immunofluorescence	High grade Serous Ovarian Cancer
FFPE-TMA	NECC170	Multiplex Immunofluorescence	High grade Serous Ovarian Cancer
FFPE-TMA	NECC171	Multiplex Immunofluorescence	High grade Serous Ovarian Cancer
FFPE-TMA	NECC172	Multiplex Immunofluorescence	High grade Serous Ovarian Cancer
FFPE-TMA	NECC173	Multiplex Immunofluorescence	High grade Serous Ovarian Cancer
FFPE-TMA	NECC174	Multiplex Immunofluorescence	High grade Serous Ovarian Cancer
FFPE-TMA	NECC175	Multiplex Immunofluorescence	High grade Serous Ovarian Cancer
FFPE-TMA	NECC176	Multiplex Immunofluorescence	High grade Serous Ovarian Cancer
FFPE-TMA	NECC177	Multiplex Immunofluorescence	High grade Serous Ovarian Cancer
FFPE-TMA	NECC178	Multiplex Immunofluorescence	High grade Serous Ovarian Cancer
FFPE-TMA	NECC179	Multiplex Immunofluorescence	High grade Serous Ovarian Cancer
FFPE-TMA	NECC180	Multiplex Immunofluorescence	High grade Serous Ovarian Cancer
FFPE-TMA	NHS1	Multiplex Immunofluorescence	High grade Serous Ovarian Cancer
FFPE-TMA	NHS2	Multiplex Immunofluorescence	High grade Serous Ovarian Cancer
FFPE-TMA	NHS3	Multiplex Immunofluorescence	High grade Serous Ovarian Cancer
FFPE-TMA	NHS4	Multiplex Immunofluorescence	High grade Serous Ovarian Cancer
FFPE-TMA	NHS5	Multiplex Immunofluorescence	High grade Serous Ovarian Cancer
FFPE-TMA	NHS6	Multiplex Immunofluorescence	High grade Serous Ovarian Cancer
FFPE-TMA	NHS7	Multiplex Immunofluorescence	High grade Serous Ovarian Cancer

FFPE-TMA	NHS8	Multiplex Immunofluorescence	High grade Serous Ovarian Cancer
FFPE-TMA	NHS9	Multiplex Immunofluorescence	High grade Serous Ovarian Cancer
FFPE-TMA	NHS10	Multiplex Immunofluorescence	High grade Serous Ovarian Cancer
FFPE-TMA	NHS11	Multiplex Immunofluorescence	High grade Serous Ovarian Cancer
FFPE-TMA	NHS12	Multiplex Immunofluorescence	High grade Serous Ovarian Cancer
FFPE-TMA	NHS13	Multiplex Immunofluorescence	High grade Serous Ovarian Cancer
FFPE-TMA	NHS14	Multiplex Immunofluorescence	High grade Serous Ovarian Cancer
FFPE-TMA	NHS15	Multiplex Immunofluorescence	High grade Serous Ovarian Cancer
FFPE-TMA	NHS16	Multiplex Immunofluorescence	High grade Serous Ovarian Cancer
FFPE-TMA	NHS17	Multiplex Immunofluorescence	High grade Serous Ovarian Cancer
FFPE-TMA	NHS18	Multiplex Immunofluorescence	High grade Serous Ovarian Cancer
FFPE-TMA	NHS19	Multiplex Immunofluorescence	High grade Serous Ovarian Cancer
FFPE-TMA	NHS20	Multiplex Immunofluorescence	High grade Serous Ovarian Cancer
FFPE-TMA	NHS21	Multiplex Immunofluorescence	High grade Serous Ovarian Cancer
FFPE-TMA	NHS22	Multiplex Immunofluorescence	High grade Serous Ovarian Cancer
FFPE-TMA	NHS23	Multiplex Immunofluorescence	High grade Serous Ovarian Cancer
FFPE-TMA	NHS24	Multiplex Immunofluorescence	High grade Serous Ovarian Cancer
FFPE-TMA	NHS25	Multiplex Immunofluorescence	High grade Serous Ovarian Cancer
FFPE-TMA	NHS26	Multiplex Immunofluorescence	High grade Serous Ovarian Cancer
FFPE-TMA	NHS27	Multiplex Immunofluorescence	High grade Serous Ovarian Cancer
FFPE-TMA	NHS28	Multiplex Immunofluorescence	High grade Serous Ovarian Cancer
FFPE-TMA	NHS29	Multiplex Immunofluorescence	High grade Serous Ovarian Cancer
FFPE-TMA	NHS30	Multiplex Immunofluorescence	High grade Serous Ovarian Cancer
FFPE-TMA	NHS31	Multiplex Immunofluorescence	High grade Serous Ovarian Cancer
FFPE-TMA	NHS32	Multiplex Immunofluorescence	High grade Serous Ovarian Cancer
FFPE-TMA	NHS33	Multiplex Immunofluorescence	High grade Serous Ovarian Cancer
FFPE-TMA	NHS34	Multiplex Immunofluorescence	High grade Serous Ovarian Cancer
FFPE-TMA	NHS35	Multiplex Immunofluorescence	High grade Serous Ovarian Cancer
FFPE-TMA	NHS36	Multiplex Immunofluorescence	High grade Serous Ovarian Cancer
FFPE-TMA	NHS37	Multiplex Immunofluorescence	High grade Serous Ovarian Cancer
FFPE-TMA	NHS38	Multiplex Immunofluorescence	High grade Serous Ovarian Cancer
FFPE-TMA	NHS39	Multiplex Immunofluorescence	High grade Serous Ovarian Cancer
FFPE-TMA	NHS40	Multiplex Immunofluorescence	High grade Serous Ovarian Cancer
FFPE-TMA	NHS41	Multiplex Immunofluorescence	High grade Serous Ovarian Cancer
FFPE-TMA	NHS42	Multiplex Immunofluorescence	High grade Serous Ovarian Cancer
FFPE-TMA	NHS43	Multiplex Immunofluorescence	High grade Serous Ovarian Cancer
FFPE-TMA	NHS44	Multiplex Immunofluorescence	High grade Serous Ovarian Cancer
FFPE-TMA	NHS45	Multiplex Immunofluorescence	High grade Serous Ovarian Cancer
FFPE-TMA	NHS46	Multiplex Immunofluorescence	High grade Serous Ovarian Cancer
FFPE-TMA	NHS47	Multiplex Immunofluorescence	High grade Serous Ovarian Cancer
FFPE-TMA	NHS48	Multiplex Immunofluorescence	High grade Serous Ovarian Cancer
FFPE-TMA	NHS49	Multiplex Immunofluorescence	High grade Serous Ovarian Cancer
FFPE-TMA	NHS50	Multiplex Immunofluorescence	High grade Serous Ovarian Cancer
FFPE-TMA	NHS51	Multiplex Immunofluorescence	High grade Serous Ovarian Cancer
FFPE-TMA	NHS52	Multiplex Immunofluorescence	High grade Serous Ovarian Cancer
FFPE-TMA	NHS53	Multiplex Immunofluorescence	High grade Serous Ovarian Cancer
FFPE-TMA	NHS54	Multiplex Immunofluorescence	High grade Serous Ovarian Cancer
FFPE-TMA	NHS55	Multiplex Immunofluorescence	High grade Serous Ovarian Cancer
FFPE-TMA	NHS56	Multiplex Immunofluorescence	High grade Serous Ovarian Cancer
FFPE-TMA	NHS57	Multiplex Immunofluorescence	High grade Serous Ovarian Cancer
FFPE-TMA	NHS58	Multiplex Immunofluorescence	High grade Serous Ovarian Cancer
FFPE-TMA	NHS59	Multiplex Immunofluorescence	High grade Serous Ovarian Cancer
FFPE-TMA	NHS60	Multiplex Immunofluorescence	High grade Serous Ovarian Cancer

FFPE-TMA	NHS61	Multiplex Immunofluorescence	High grade Serous Ovarian Cancer
FFPE-TMA	NHS62	Multiplex Immunofluorescence	High grade Serous Ovarian Cancer
FFPE-TMA	NHS63	Multiplex Immunofluorescence	High grade Serous Ovarian Cancer
FFPE-TMA	NHS64	Multiplex Immunofluorescence	High grade Serous Ovarian Cancer
FFPE-TMA	NHS65	Multiplex Immunofluorescence	High grade Serous Ovarian Cancer
FFPE-TMA	NHS66	Multiplex Immunofluorescence	High grade Serous Ovarian Cancer
FFPE-TMA	NHS67	Multiplex Immunofluorescence	High grade Serous Ovarian Cancer
FFPE-TMA	NHS68	Multiplex Immunofluorescence	High grade Serous Ovarian Cancer
FFPE-TMA	NHS69	Multiplex Immunofluorescence	High grade Serous Ovarian Cancer
FFPE-TMA	NHS70	Multiplex Immunofluorescence	High grade Serous Ovarian Cancer
FFPE-TMA	NHS71	Multiplex Immunofluorescence	High grade Serous Ovarian Cancer
FFPE-TMA	NHS72	Multiplex Immunofluorescence	High grade Serous Ovarian Cancer
FFPE-TMA	NHS73	Multiplex Immunofluorescence	High grade Serous Ovarian Cancer
FFPE-TMA	NHS74	Multiplex Immunofluorescence	High grade Serous Ovarian Cancer
FFPE-TMA	NHS75	Multiplex Immunofluorescence	High grade Serous Ovarian Cancer
FFPE-TMA	NHS76	Multiplex Immunofluorescence	High grade Serous Ovarian Cancer
FFPE-TMA	NHS77	Multiplex Immunofluorescence	High grade Serous Ovarian Cancer
FFPE-TMA	NHS78	Multiplex Immunofluorescence	High grade Serous Ovarian Cancer
FFPE-TMA	NHS79	Multiplex Immunofluorescence	High grade Serous Ovarian Cancer
FFPE-TMA	NHS80	Multiplex Immunofluorescence	High grade Serous Ovarian Cancer
FFPE-TMA	NHS81	Multiplex Immunofluorescence	High grade Serous Ovarian Cancer
FFPE-TMA	NHS82	Multiplex Immunofluorescence	High grade Serous Ovarian Cancer
FFPE-TMA	NHS83	Multiplex Immunofluorescence	High grade Serous Ovarian Cancer
FFPE-TMA	NHS84	Multiplex Immunofluorescence	High grade Serous Ovarian Cancer
FFPE-TMA	NHS85	Multiplex Immunofluorescence	High grade Serous Ovarian Cancer
FFPE-TMA	NHS86	Multiplex Immunofluorescence	High grade Serous Ovarian Cancer
FFPE-TMA	NHS87	Multiplex Immunofluorescence	High grade Serous Ovarian Cancer
FFPE-TMA	NHS88	Multiplex Immunofluorescence	High grade Serous Ovarian Cancer
FFPE-TMA	NHS89	Multiplex Immunofluorescence	High grade Serous Ovarian Cancer
FFPE-TMA	NHS90	Multiplex Immunofluorescence	High grade Serous Ovarian Cancer
FFPE-TMA	NHS91	Multiplex Immunofluorescence	High grade Serous Ovarian Cancer
FFPE-TMA	NHS92	Multiplex Immunofluorescence	High grade Serous Ovarian Cancer
FFPE-TMA	NHS93	Multiplex Immunofluorescence	High grade Serous Ovarian Cancer
FFPE-TMA	NHS94	Multiplex Immunofluorescence	High grade Serous Ovarian Cancer
FFPE-TMA	NHS95	Multiplex Immunofluorescence	High grade Serous Ovarian Cancer
FFPE-TMA	NHS96	Multiplex Immunofluorescence	High grade Serous Ovarian Cancer
FFPE-TMA	NHS97	Multiplex Immunofluorescence	High grade Serous Ovarian Cancer
FFPE-TMA	NHS98	Multiplex Immunofluorescence	High grade Serous Ovarian Cancer
FFPE-TMA	NHS99	Multiplex Immunofluorescence	High grade Serous Ovarian Cancer
FFPE-TMA	NHS100	Multiplex Immunofluorescence	High grade Serous Ovarian Cancer
FFPE-TMA	NHS101	Multiplex Immunofluorescence	High grade Serous Ovarian Cancer
FFPE-TMA	NHS102	Multiplex Immunofluorescence	High grade Serous Ovarian Cancer
FFPE-TMA	NHS103	Multiplex Immunofluorescence	High grade Serous Ovarian Cancer
FFPE-TMA	NHS104	Multiplex Immunofluorescence	High grade Serous Ovarian Cancer
FFPE-TMA	NHS105	Multiplex Immunofluorescence	High grade Serous Ovarian Cancer
FFPE-TMA	NHS106	Multiplex Immunofluorescence	High grade Serous Ovarian Cancer
FFPE-TMA	NHS107	Multiplex Immunofluorescence	High grade Serous Ovarian Cancer
FFPE-TMA	NHS108	Multiplex Immunofluorescence	High grade Serous Ovarian Cancer
FFPE-TMA	NHS109	Multiplex Immunofluorescence	High grade Serous Ovarian Cancer
FFPE-TMA	NHS110	Multiplex Immunofluorescence	High grade Serous Ovarian Cancer
FFPE-TMA	NHS111	Multiplex Immunofluorescence	High grade Serous Ovarian Cancer
FFPE-TMA	NHS112	Multiplex Immunofluorescence	High grade Serous Ovarian Cancer
FFPE-TMA	NHS113	Multiplex Immunofluorescence	High grade Serous Ovarian Cancer

FFPE-TMA	NHS114	Multiplex Immunofluorescence	High grade Serous Ovarian Cancer
FFPE-TMA	NHS115	Multiplex Immunofluorescence	High grade Serous Ovarian Cancer
FFPE-TMA	NHS116	Multiplex Immunofluorescence	High grade Serous Ovarian Cancer
FFPE-TMA	NHS117	Multiplex Immunofluorescence	High grade Serous Ovarian Cancer
FFPE-TMA	NHS118	Multiplex Immunofluorescence	High grade Serous Ovarian Cancer
FFPE-TMA	NHS119	Multiplex Immunofluorescence	High grade Serous Ovarian Cancer
FFPE-TMA	NHS120	Multiplex Immunofluorescence	High grade Serous Ovarian Cancer
FFPE-TMA	NHS121	Multiplex Immunofluorescence	High grade Serous Ovarian Cancer
FFPE-TMA	NHS122	Multiplex Immunofluorescence	High grade Serous Ovarian Cancer
FFPE-TMA	NHS123	Multiplex Immunofluorescence	High grade Serous Ovarian Cancer
FFPE-TMA	NHS124	Multiplex Immunofluorescence	High grade Serous Ovarian Cancer
FFPE-TMA	NHS125	Multiplex Immunofluorescence	High grade Serous Ovarian Cancer
FFPE-TMA	NHS126	Multiplex Immunofluorescence	High grade Serous Ovarian Cancer
FFPE-TMA	NHS127	Multiplex Immunofluorescence	High grade Serous Ovarian Cancer
FFPE-TMA	NHS128	Multiplex Immunofluorescence	High grade Serous Ovarian Cancer
FFPE-TMA	NHS129	Multiplex Immunofluorescence	High grade Serous Ovarian Cancer
FFPE-TMA	NHS130	Multiplex Immunofluorescence	High grade Serous Ovarian Cancer
FFPE-TMA	NHS131	Multiplex Immunofluorescence	High grade Serous Ovarian Cancer
FFPE-TMA	NHS132	Multiplex Immunofluorescence	High grade Serous Ovarian Cancer
FFPE-TMA	NHS133	Multiplex Immunofluorescence	High grade Serous Ovarian Cancer
FFPE-TMA	NHS134	Multiplex Immunofluorescence	High grade Serous Ovarian Cancer
FFPE-TMA	NHS135	Multiplex Immunofluorescence	High grade Serous Ovarian Cancer
FFPE-TMA	NHS136	Multiplex Immunofluorescence	High grade Serous Ovarian Cancer
FFPE-TMA	NHS137	Multiplex Immunofluorescence	High grade Serous Ovarian Cancer
FFPE-TMA	NHS138	Multiplex Immunofluorescence	High grade Serous Ovarian Cancer
FFPE-TMA	NHS139	Multiplex Immunofluorescence	High grade Serous Ovarian Cancer
FFPE-TMA	NHS140	Multiplex Immunofluorescence	High grade Serous Ovarian Cancer
FFPE-TMA	NHS141	Multiplex Immunofluorescence	High grade Serous Ovarian Cancer
FFPE-TMA	NHS142	Multiplex Immunofluorescence	High grade Serous Ovarian Cancer
FFPE-TMA	NHS143	Multiplex Immunofluorescence	High grade Serous Ovarian Cancer
FFPE-TMA	NHS144	Multiplex Immunofluorescence	High grade Serous Ovarian Cancer
FFPE-TMA	NHS145	Multiplex Immunofluorescence	High grade Serous Ovarian Cancer
FFPE-TMA	NHS146	Multiplex Immunofluorescence	High grade Serous Ovarian Cancer
FFPE-TMA	NHS147	Multiplex Immunofluorescence	High grade Serous Ovarian Cancer
FFPE-TMA	NHS148	Multiplex Immunofluorescence	High grade Serous Ovarian Cancer
FFPE-TMA	NHS149	Multiplex Immunofluorescence	High grade Serous Ovarian Cancer
FFPE-TMA	NHS150	Multiplex Immunofluorescence	High grade Serous Ovarian Cancer
FFPE-TMA	NHS151	Multiplex Immunofluorescence	High grade Serous Ovarian Cancer
FFPE-TMA	NHS152	Multiplex Immunofluorescence	High grade Serous Ovarian Cancer
FFPE-TMA	NHS153	Multiplex Immunofluorescence	High grade Serous Ovarian Cancer
FFPE-TMA	NHS154	Multiplex Immunofluorescence	High grade Serous Ovarian Cancer
FFPE-TMA	NHS155	Multiplex Immunofluorescence	High grade Serous Ovarian Cancer
FFPE-TMA	NHS156	Multiplex Immunofluorescence	High grade Serous Ovarian Cancer
FFPE-TMA	NHS157	Multiplex Immunofluorescence	High grade Serous Ovarian Cancer
FFPE-TMA	NHS158	Multiplex Immunofluorescence	High grade Serous Ovarian Cancer
FFPE-TMA	NHS159	Multiplex Immunofluorescence	High grade Serous Ovarian Cancer
FFPE-TMA	NHS160	Multiplex Immunofluorescence	High grade Serous Ovarian Cancer
FFPE-TMA	NHS161	Multiplex Immunofluorescence	High grade Serous Ovarian Cancer
FFPE-TMA	NHS162	Multiplex Immunofluorescence	High grade Serous Ovarian Cancer
FFPE-TMA	NHS163	Multiplex Immunofluorescence	High grade Serous Ovarian Cancer
FFPE-TMA	NHS164	Multiplex Immunofluorescence	High grade Serous Ovarian Cancer
FFPE-TMA	NHS165	Multiplex Immunofluorescence	High grade Serous Ovarian Cancer
FFPE-TMA	NHS166	Multiplex Immunofluorescence	High grade Serous Ovarian Cancer

FFPE-TMA	NHS167	Multiplex Immunofluorescence	High grade Serous Ovarian Cancer
FFPE-TMA	NHS168	Multiplex Immunofluorescence	High grade Serous Ovarian Cancer
FFPE-TMA	NHS169	Multiplex Immunofluorescence	High grade Serous Ovarian Cancer
FFPE-TMA	NHS170	Multiplex Immunofluorescence	High grade Serous Ovarian Cancer
FFPE-TMA	NHS171	Multiplex Immunofluorescence	High grade Serous Ovarian Cancer
FFPE-TMA	NHS172	Multiplex Immunofluorescence	High grade Serous Ovarian Cancer
FFPE-TMA	NHS173	Multiplex Immunofluorescence	High grade Serous Ovarian Cancer
FFPE-TMA	NHS174	Multiplex Immunofluorescence	High grade Serous Ovarian Cancer
FFPE-TMA	NHS175	Multiplex Immunofluorescence	High grade Serous Ovarian Cancer
FFPE-TMA	NHS176	Multiplex Immunofluorescence	High grade Serous Ovarian Cancer
FFPE-TMA	NHS177	Multiplex Immunofluorescence	High grade Serous Ovarian Cancer
FFPE-TMA	NHS178	Multiplex Immunofluorescence	High grade Serous Ovarian Cancer
FFPE-TMA	NHS179	Multiplex Immunofluorescence	High grade Serous Ovarian Cancer
FFPE-TMA	NHS180	Multiplex Immunofluorescence	High grade Serous Ovarian Cancer
FFPE-TMA	NHS181	Multiplex Immunofluorescence	High grade Serous Ovarian Cancer
FFPE-TMA	NHS182	Multiplex Immunofluorescence	High grade Serous Ovarian Cancer
FFPE-TMA	NHS183	Multiplex Immunofluorescence	High grade Serous Ovarian Cancer
FFPE-TMA	NHS184	Multiplex Immunofluorescence	High grade Serous Ovarian Cancer
FFPE-TMA	NHS185	Multiplex Immunofluorescence	High grade Serous Ovarian Cancer
FFPE-TMA	NHS186	Multiplex Immunofluorescence	High grade Serous Ovarian Cancer
FFPE-TMA	NHS187	Multiplex Immunofluorescence	High grade Serous Ovarian Cancer
FFPE-TMA	NHS188	Multiplex Immunofluorescence	High grade Serous Ovarian Cancer
FFPE-TMA	NHS189	Multiplex Immunofluorescence	High grade Serous Ovarian Cancer
FFPE-TMA	NHS190	Multiplex Immunofluorescence	High grade Serous Ovarian Cancer
FFPE-TMA	NHS191	Multiplex Immunofluorescence	High grade Serous Ovarian Cancer
FFPE-TMA	NHS192	Multiplex Immunofluorescence	High grade Serous Ovarian Cancer
FFPE-TMA	NHS193	Multiplex Immunofluorescence	High grade Serous Ovarian Cancer
FFPE-TMA	NHS194	Multiplex Immunofluorescence	High grade Serous Ovarian Cancer
FFPE-TMA	NHS195	Multiplex Immunofluorescence	High grade Serous Ovarian Cancer
FFPE-TMA	NHS196	Multiplex Immunofluorescence	High grade Serous Ovarian Cancer
FFPE-TMA	NHS197	Multiplex Immunofluorescence	High grade Serous Ovarian Cancer
FFPE-TMA	NHS198	Multiplex Immunofluorescence	High grade Serous Ovarian Cancer
FFPE-TMA	NHS199	Multiplex Immunofluorescence	High grade Serous Ovarian Cancer
FFPE-TMA	NHS200	Multiplex Immunofluorescence	High grade Serous Ovarian Cancer
FFPE-TMA	NHS201	Multiplex Immunofluorescence	High grade Serous Ovarian Cancer
FFPE-TMA	NHS202	Multiplex Immunofluorescence	High grade Serous Ovarian Cancer
FFPE-TMA	NHS203	Multiplex Immunofluorescence	High grade Serous Ovarian Cancer
FFPE-TMA	NHS204	Multiplex Immunofluorescence	High grade Serous Ovarian Cancer
FFPE-TMA	NHS205	Multiplex Immunofluorescence	High grade Serous Ovarian Cancer
FFPE-TMA	NHS206	Multiplex Immunofluorescence	High grade Serous Ovarian Cancer
FFPE-TMA	NHS207	Multiplex Immunofluorescence	High grade Serous Ovarian Cancer
FFPE-TMA	NHS208	Multiplex Immunofluorescence	High grade Serous Ovarian Cancer
FFPE-TMA	NHS209	Multiplex Immunofluorescence	High grade Serous Ovarian Cancer
FFPE-TMA	NHS210	Multiplex Immunofluorescence	High grade Serous Ovarian Cancer
FFPE-TMA	NHS211	Multiplex Immunofluorescence	High grade Serous Ovarian Cancer
FFPE-TMA	NHS212	Multiplex Immunofluorescence	High grade Serous Ovarian Cancer
FFPE-TMA	NHS213	Multiplex Immunofluorescence	High grade Serous Ovarian Cancer
FFPE-TMA	NHS214	Multiplex Immunofluorescence	High grade Serous Ovarian Cancer
FFPE-TMA	NHS215	Multiplex Immunofluorescence	High grade Serous Ovarian Cancer
FFPE-TMA	NHS216	Multiplex Immunofluorescence	High grade Serous Ovarian Cancer
FFPE-TMA	NHS217	Multiplex Immunofluorescence	High grade Serous Ovarian Cancer
FFPE-TMA	NHS218	Multiplex Immunofluorescence	High grade Serous Ovarian Cancer
FFPE-TMA	NHS219	Multiplex Immunofluorescence	High grade Serous Ovarian Cancer

FFPE-TMA	NHS220	Multiplex Immunofluorescence	High grade Serous Ovarian Cancer
FFPE-TMA	NHS221	Multiplex Immunofluorescence	High grade Serous Ovarian Cancer
FFPE-TMA	NHS222	Multiplex Immunofluorescence	High grade Serous Ovarian Cancer
FFPE-TMA	NHS223	Multiplex Immunofluorescence	High grade Serous Ovarian Cancer
FFPE-TMA	NHS224	Multiplex Immunofluorescence	High grade Serous Ovarian Cancer
FFPE-TMA	NHS225	Multiplex Immunofluorescence	High grade Serous Ovarian Cancer
FFPE-TMA	NHS226	Multiplex Immunofluorescence	High grade Serous Ovarian Cancer
FFPE-TMA	NHS227	Multiplex Immunofluorescence	High grade Serous Ovarian Cancer
FFPE-TMA	NHS228	Multiplex Immunofluorescence	High grade Serous Ovarian Cancer
FFPE-TMA	NHS229	Multiplex Immunofluorescence	High grade Serous Ovarian Cancer
FFPE-TMA	NHS230	Multiplex Immunofluorescence	High grade Serous Ovarian Cancer
FFPE-TMA	NHS231	Multiplex Immunofluorescence	High grade Serous Ovarian Cancer
FFPE-TMA	NHS232	Multiplex Immunofluorescence	High grade Serous Ovarian Cancer
FFPE-TMA	NHS233	Multiplex Immunofluorescence	High grade Serous Ovarian Cancer
FFPE-TMA	NHS234	Multiplex Immunofluorescence	High grade Serous Ovarian Cancer
FFPE-TMA	NHS235	Multiplex Immunofluorescence	High grade Serous Ovarian Cancer
FFPE-TMA	NHS236	Multiplex Immunofluorescence	High grade Serous Ovarian Cancer
FFPE-TMA	NHS237	Multiplex Immunofluorescence	High grade Serous Ovarian Cancer
FFPE-TMA	NHS238	Multiplex Immunofluorescence	High grade Serous Ovarian Cancer
FFPE-TMA	NHS239	Multiplex Immunofluorescence	High grade Serous Ovarian Cancer
FFPE-TMA	NHS240	Multiplex Immunofluorescence	High grade Serous Ovarian Cancer
FFPE-TMA	NHS241	Multiplex Immunofluorescence	High grade Serous Ovarian Cancer
FFPE-TMA	NHS242	Multiplex Immunofluorescence	High grade Serous Ovarian Cancer
FFPE-TMA	NHS243	Multiplex Immunofluorescence	High grade Serous Ovarian Cancer
FFPE-TMA	NHS244	Multiplex Immunofluorescence	High grade Serous Ovarian Cancer
FFPE-TMA	NHS245	Multiplex Immunofluorescence	High grade Serous Ovarian Cancer
FFPE-TMA	NHS246	Multiplex Immunofluorescence	High grade Serous Ovarian Cancer
FFPE-TMA	NHS247	Multiplex Immunofluorescence	High grade Serous Ovarian Cancer
FFPE-TMA	NHS248	Multiplex Immunofluorescence	High grade Serous Ovarian Cancer
FFPE-TMA	NHS249	Multiplex Immunofluorescence	High grade Serous Ovarian Cancer
FFPE-TMA	NHS250	Multiplex Immunofluorescence	High grade Serous Ovarian Cancer
FFPE-TMA	NHS251	Multiplex Immunofluorescence	High grade Serous Ovarian Cancer
FFPE-TMA	NHS252	Multiplex Immunofluorescence	High grade Serous Ovarian Cancer
FFPE-TMA	NHS253	Multiplex Immunofluorescence	High grade Serous Ovarian Cancer
FFPE-TMA	NHS254	Multiplex Immunofluorescence	High grade Serous Ovarian Cancer
FFPE-TMA	NHS255	Multiplex Immunofluorescence	High grade Serous Ovarian Cancer
FFPE-TMA	NHS256	Multiplex Immunofluorescence	High grade Serous Ovarian Cancer
FFPE-TMA	NHS257	Multiplex Immunofluorescence	High grade Serous Ovarian Cancer
FFPE-TMA	NHS258	Multiplex Immunofluorescence	High grade Serous Ovarian Cancer
FFPE-TMA	NHS259	Multiplex Immunofluorescence	High grade Serous Ovarian Cancer
FFPE-TMA	NHS260	Multiplex Immunofluorescence	High grade Serous Ovarian Cancer
FFPE-TMA	NHS261	Multiplex Immunofluorescence	High grade Serous Ovarian Cancer

Supplementary Table 11

Statistical information associated with Extended data figure 6e

**Cell type**	**Treatment**	**Number of cells (*n*) quantified**
WT OVCAR3	αTSPAN7	338
PIGR^{CRSIPR} OVCAR3	αTSPAN7	319
WT OVCAR3	αBDNF	400
PIGR^{CRSIPR} OVCAR3	αBDNF	346
WT OVCAR3	iIgA	244
PIGR^{CRSIPR} OVCAR3	iIgA	311
WT OVCAR3	iIgG	420

**Between treatment groups**	*P* value	Test performed
WT: **α**TSPAN7 *vs.* PIGR^{CRISPR}: **α**TSPAN7	<0.0001	Unpaired two-tailed t-test
WT: **α**BDNF *vs.* PIGR^{CRISPR}: **α**BDNF	<0.0001	Unpaired two-tailed t-test
WT:iIgA *vs.* PIGR^{CRISPR}:iIgA	<0.0001	Unpaired two-tailed t-test

Supplementary Table 12

Statistical information associated with Extended data figure 6g

Growth curve statistics

**Between treatment groups**	***P* value**	**Test performed**
Tumour#1: PIGR^{+}:PBS *vs.* PIGR^{+}:iIgA	0.0155	Paired two-tailed t-test
Tumour#1: PIGR^{+}:iIgA *vs.* PIGR^{+}:Ab1	0.0144	Paired two-tailed t-test
Tumour#1: PIGR^{+}:Ab1 *vs.* PIGR^{CRISPR}:Ab1	0.0172	Paired two-tailed t-test
Tumour#1: PIGR^{+}:iIgA *vs.* PIGR^{CRISPR}:iIgA	0.0125	Paired two-tailed t-test
Tumour#1: PIGR^{CRISPR}:iIgA *vs.* PIGR^{CRISPR}:Ab1	0.0120	Paired two-tailed t-test
Tumour#2: PIGR^{+}:PBS *vs.* PIGR^{+}:iIgA	0.0175	Paired two-tailed t-test
Tumour#2: PIGR^{+}:iIgA *vs.* PIGR^{+}:Ab2	0.0044	Paired two-tailed t-test
Tumour#2: PIGR^{+}:iIgA *vs.* PIGR^{+}:Ab3	0.0061	Paired two-tailed t-test
Tumour#2: PIGR^{+}:iIgA *vs.* PIGR^{CRISPR}:iIgA	0.0155	Paired two-tailed t-test
Tumour#2: PIGR^{+}:Ab2 *vs.* PIGR^{CRISPR}	0.0113	Paired two-tailed t-test
Tumour#2: PIGR^{+}:Ab3 *vs.* PIGR^{CRISPR}	0.0089	Paired two-tailed t-test
Tumour#2: PIGR^{CRISPR}:iIgA *vs.* PIGR^{CRISPR}	0.0095	Paired two-tailed t-test
Tumour#2: PIGR^{CRISPR}:iIgA *vs.* PIGR^{CRISPR}	0.0107	Paired two-tailed t-test

Tumour weight statistics

**Between treatment groups**	***P* value**	**Test performed**
Tumour#1: PIGR^{+}:iIgA *vs.* PIGR^{+}:Ab1	<0.0001	Unpaired two-tailed t-test
Tumour#1: PIGR^{+}:iIgA *vs.* PIGR^{CRISPR}:iIgA	<0.0001	Unpaired two-tailed t-test
Tumour#1: PIGR^{+}:Ab1 *vs.* PIGR^{CRISPR}:Ab1	<0.0001	Unpaired two-tailed t-test
Tumour#1: PIGR^{CRISPR}:iIgA *vs.* PIGR^{CRISPR}:Ab1	<0.0001	Unpaired two-tailed t-test
Tumour#2: PIGR^{+}:PBS *vs.* PIGR^{+}:iIgA	<0.0001	Unpaired two-tailed t-test
Tumour#2: PIGR^{+}:iIgA *vs.* PIGR^{+}:Ab2	<0.0001	Unpaired two-tailed t-test
Tumour#2: PIGR^{+}:iIgA *vs.* PIGR^{+}:Ab3	<0.0001	Unpaired two-tailed t-test
Tumour#2: PIGR^{+}:Ab2 *vs.* PIGR^{CRISPR}:Ab2	<0.0001	Unpaired two-tailed t-test
Tumour#2: PIGR^{+}:Ab3 *vs.* PIGR^{CRISPR}:Ab3	<0.0001	Unpaired two-tailed t-test
Tumour#2: PIGR^{CRISPR}:iIgA *vs.* PIGR^{CRISPR}:Ab2	<0.0001	Unpaired two-tailed t-test
Tumour#2: PIGR^{CRISPR}:iIgA *vs.* PIGR^{CRISPR}:Ab3	<0.0001	Unpaired two-tailed t-test
