# Supplementary material for: IgA transcytosis and antigen recognition govern ovarian cancer immunity
Source: Nature. 2021 Feb 3;591(7850):464–70. doi: 10.1038/s41586-020-03144-0 (PMC7969354; doi:10.1038/s41586-020-03144-0)
Supplement: Supplementary file 2 — Reporting Summary [file 41586_2020_3144_MOESM2_ESM.pdf]

nature research

Corresponding author(s): Jose R Conejo-Garcia

Last updated by author(s): Dec 4, 2020

Reporting Summary

Nature Research wishes to improve the reproducibility of the work that we publish. This form provides structure for consistency and transparency in reporting. For further information on Nature Research policies, see our Editorial Policies and the Editorial Policy Checklist.

Statistics

For all statistical analyses, confirm that the following items are present in the figure legend, table legend, main text, or Methods section.

n/a	Confirmed	
☐	☒	The exact sample size (*n*) for each experimental group/condition, given as a discrete number and unit of measurement
☐	☒	A statement on whether measurements were taken from distinct samples or whether the same sample was measured repeatedly
☐	☒	The statistical test(s) used AND whether they are one- or two-sided<br>*Only common tests should be described solely by name; describe more complex techniques in the Methods section.*
☐	☒	A description of all covariates tested
☐	☒	A description of any assumptions or corrections, such as tests of normality and adjustment for multiple comparisons
☐	☒	A full description of the statistical parameters including central tendency (e.g. means) or other basic estimates (e.g. regression coefficient) AND variation (e.g. standard deviation) or associated estimates of uncertainty (e.g. confidence intervals)
☐	☒	For null hypothesis testing, the test statistic (e.g. *F*, *t*, *r*) with confidence intervals, effect sizes, degrees of freedom and *P* value noted<br>*Give P values as exact values whenever suitable.*
☒	☐	For Bayesian analysis, information on the choice of priors and Markov chain Monte Carlo settings
☒	☐	For hierarchical and complex designs, identification of the appropriate level for tests and full reporting of outcomes
☐	☒	Estimates of effect sizes (e.g. Cohen's *d*, Pearson's *r*), indicating how they were calculated

Our web collection on statistics for biologists contains articles on many of the points above.

Software and code

Policy information about availability of computer code

Data collection	FACS data was collected from BD FACS LSR II and BD FACS ARIA using BD FACS Diva v8.0.1. Multiplex data was collected from PerkinElmer Vectra®3 Automated Quantitative Pathology Imaging System inForm v2.4.8. RNA Sequencing reads were collected using bcl2fastq v2.20. Confocal microscopy image acquisition was performed and data collected from Leica SP8 using LAS X (v3.5.5.19976). For quantitative analysis, fluorescence image acquisition was performed in Zeiss Imager Z2 upright microscope and data was collected using ZEN 2.3 (blue edition) software. H/E histology slides were scanned in Aperio-Leica Scanner Console (v102.0.7.5) and data were collected.
Data analysis	All statistical tests were run using Graphpad Prism (v7.0) or R (v3.6.1). Flow cytometry data was analyzed with FlowJo v10.7.1. Dot plots from Multiplex images were done using FCS Image v7.0. Multiplex images were analyzed in inForm (v2.4.8) and HALO (v3.0.311.328). RNA-seq sequence processing and statistics was performed using cutadapt (v1.8.1), STAR (v2.5.3a), featureCounts (v1.5.3), htseq-count (v0.6.1), and DESeq2 (v1.30.0). GSEA (v4.0.2) was performed using gene sets from the MSigDB database. Single-cell V(D)J (BCR) sequence data was analyzed using CellRanger V(D)J (v3.1.0). RNA-seq and single-cell data visualization was performed in R (v3.6.1). CZI image files were imported into Definiens Tissue Studio (v4.7) and analyzed.

For manuscripts utilizing custom algorithms or software that are central to the research but not yet described in published literature, software must be made available to editors and reviewers. We strongly encourage code deposition in a community repository (e.g. GitHub). See the Nature Research guidelines for submitting code & software for further information.

Data

Policy information about availability of data

All manuscripts must include a data availability statement. This statement should provide the following information, where applicable:

- Accession codes, unique identifiers, or web links for publicly available datasets
- A list of figures that have associated raw data
- A description of any restrictions on data availability

The RNA sequencing data and single cell BCR sequencing data related to this study are available at the NCBI Gene Expression Omnibus (GEO) under accession number GSE146820. The mass spectrometry proteomics data are available in PRIDE with identifier PXD018079. Source data are provided with this paper. Molecular and clinical data from The Cancer Genome Atlas for Ovarian Serous Cystadenocarcinoma (OV) are available at the cBio Cancer Genomics Portal (http://www.cbioportal.org/), Broad Firehose website (https://gdac.broadinstitute.org/), and Genomic Data Commons Data Portal (https://portal.gdc.cancer.gov/). Source data are provided with this paper. The datasets generated during the current study are available from the corresponding author upon reasonable request.

Field-specific reporting

Please select the one below that is the best fit for your research. If you are not sure, read the appropriate sections before making your selection.

☒ Life sciences ☐ Behavioural & social sciences ☐ Ecological, evolutionary & environmental sciences

For a reference copy of the document with all sections, see nature.com/documents/nr-reporting-summary-flat.pdf

Life sciences study design

All studies must disclose on these points even when the disclosure is negative.

Sample size	Sample size for every experiment has been described in the manuscript and is based on the availability of adequate samples. No sample size calculations were performed prior to the study for human specimens. For most functional in vitro analyses, sample sizes were chosen based on the availability of target cells. Animal experiments used at least five mice per group per experiments. Since this study focuses on ovarian cancer, only female mice were included in the experimental design.
Data exclusions	No data were excluded
Replication	Experiments were performed at least two times and/or with sufficient cells/animals per group to demonstrate statistical significance.
Randomization	The experiments were not randomized.<br>-HGSOC tumour and ascites specimens were obtained from de-identified patients and were not randomized. PBMC from de-identified donors without cancer were acquired and analyzed.<br>- Animals were not intentionally randomized. All animal experiments were conducted using 4-6 week-old female RAG1-deficient or NSG mice with procured from Charles River Laboratories and Jackson Laboratory, respectively.
Blinding	-Tumour volumes in mice were measured using code names on the cages and ear tags, instead of specific information about the treatments that the animals received. Apart from this, no blinding method was used for animal studies.<br>-RNA sequencing, BCR sequencing, Multiplex immunohistochemistry quantifications, fluorescence microscopy quantifications or LC-MS/MS were performed with unidentifiable demarcation.<br>-In case of in vitro experiments, samples often assigned code numbers to facilitate blinded flow cytometry, microscopy, luciferase assay. After all data were collected, the results were analyzed and decoded.<br>-For analysis of human specimens blinding is not applicable as no interventions were tested.

Reporting for specific materials, systems and methods

We require information from authors about some types of materials, experimental systems and methods used in many studies. Here, indicate whether each material, system or method listed is relevant to your study. If you are not sure if a list item applies to your research, read the appropriate section before selecting a response.

Materials & experimental systems

n/a	Involved in the study	
☐	☒	Antibodies
☐	☒	Eukaryotic cell lines
☒	☐	Palaeontology and archaeology
☐	☒	Animals and other organisms
☐	☒	Human research participants
☒	☐	Clinical data
☒	☐	Dual use research of concern

Methods

n/a	Involved in the study	
☒	☐	ChIP-seq
☐	☒	Flow cytometry
☒	☐	MRI-based neuroimaging

Antibodies

Antibodies used

anti-Human antibodies:
anti-CD45-BV786, BD Biosciences, 563716, HI30, Lot-7226988;
anti-CD3-BUV737, BD Biosciences, 564307, UCHT1, Lot- 7241949;
anti-CD19-BUV496, BD Biosciences, 564655, SJ25C1, Lot-7279814;
anti-CD20-PECy7, BioLegend, 302312, 2H7, Lot-B124738;
anti-CD38-BV421, BD Biosciences, 562445, HIT2, Lot-7019700;
anti-CD138-AlexaFluor700, BioLegend, 356512, MI15, Lot-B227191;
anti-CD27-PerCP-Cy5.5, BD Biosciences, 560612, M-T2171, Lot-7167876;
anti-EpCAM-BUV805, BD Biosciences, 748381, KS1/4, Lot-9311213;
anti-IgA-FITC, Tonbo Biosciences, 35-8016, M001, Lot-S358016001001;
anti-IgG-APC, BioLegend, 410720, M1310G05, Lot-B243888;
anti-IgM-PE, BioLegend, 314508, MHM-88, Lot-B235317;
anti-IgD-BV480, BD Biosciences, 566138, IA6-2, Lot-6294773;
anti-IgE-BV711, BD Biosciences, 744319, G7-26, Lot-8002780;
anti-pIgR, ThermoScientific, PA5-35340, Lot-VE3000758;
anti-pIgR, Abcam, ab96196, Lot-GR3263410-2;
anti-Phospho-Erk1/2, CST, 5726, D1H6G, Lot-1;
anti-Erk1/2, CST, 4696, L34F12, Lot-23;
anti-DUSP5, Abcam, ab200708, EPR19684, Lot-GR3186774-2;
anti-β-actin, CST, 4970, 13E5, Lot-15 and CST, 5125, 13E5, Lot-6;
anti-IgA, Abcam, ab124716, EPR5367-76, Lot-GR87907-13;
anti-IgG, Abcam, ab109489, EPR4421, Lot-GR3258727-4;
anti-pIgR-Secretory component, Abcam, ab3924, SC-05, GR3299752-2;
anti-CD3, Dako, A0452, Lot-20057103;
anti-CD4, Cell Marque, 104R-25, EP204, Lot- 53921;
anti-CD8, Dako, M7103, C8/144B, Lot- 20055137;
anti-CD19, Dako, M7296, LE-CD19, Lot-20067725;
anti-CD20, Dako, M0755, L26, Lot-20042864;
anti-CD138, Dako, M7228, MI15, Lot-20050508;
anti-Pan Cytokeratin, Dako, M3515, AE1/AE3, Lot-10149487;

anti-mouse antibodies:
anti-CD45-APC-Cy7, BioLegend, 103116, 30-F11, Lot-B257634;
anti-CD11b-PerCP-Cy5.5, BioLegend, 101228, M1/70, Lot-B247471;
anti-CD351-PE, BioLegend, 137306, TX61, Lot-B265236
anti-CD351, BioLegend, 137303, TX61, Lot- B275404;
anti-NK1.1-PE/Dazzle594, BioLegend, 108748, PK136, Lot- B213746;
anti-NK1.1, BioXCell, BE0036, PK136, Lot-693118J3;

Human native antibodies:
Native Human IgA, Abcam, ab91025, Lot-GR3246098-6;
Native Human IgG, Abcam, ab98981, Lot-GR3269929-2

Validation

Antibodies used in flow cytometry- Most of the primary antibodies used for flow cytometry in this study are widely used and well validated. The mentioned antibodies are tested by flow cytometry analysis by the manufacturer and/or in our lab:
anti-CD45- staining of human peripheral blood lymphocytes by flow cytometry
anti-CD3- staining of human peripheral blood lymphocytes by flow cytometry
anti-CD19- staining of human peripheral blood leucocytes by flow cytometry
anti-CD20- staining of human peripheral blood lymphocytes by flow cytometry
anti-CD38- staining of human peripheral blood lymphocytes by flow cytometry
anti-CD138- staining of human myeloma cell line U266 by flow cytometry
anti-CD27- staining of human peripheral blood lymphocytes by flow cytometry
anti-EpCAM- staining of human OVCAR3 cell line by flow cytometry
anti-IgA- staining of human peripheral blood lymphocytes flow cytometry
anti-IgG- staining of human peripheral blood lymphocytes by flow cytometry
anti-IgM- staining of by human peripheral blood lymphocytes flow cytometry
anti-IgD- staining of by human peripheral blood lymphocytes flow cytometry
anti-IgE- staining of human peripheral blood lymphocytes by flow cytometry
anti-pIgR- staining of human HepG2 cell line by flow cytometry
anti-CD45- staining of C57BL/6 mouse splenocytes by flow cytometry
anti-CD11b- staining of C57BL/6 mouse splenocytes by flow cytometry
anti-CD351- staining of Con-A stimulated C57BL/6 splenocytes by flow cytometry
anti-NK-1.1- staining of C57BL/6 mouse splenocytes by flow cytometry

----

Antibodies used in in vitro or in vivo neutralization- We used following neutralizing antibodies with relevant publication references available at the manufacturer's website:
anti-CD351- block interaction with the Fcα/μ receptor (ref: Shibuya A, et al., Nat Immunol. 2000 Nov;1(5):441-6. doi: 10.1038/80886. PMID: 11062505)
anti-NK-1.1- in vivo depletion of NK1.1 expressing cells, mice (ref: Glasner A, et al., Immunity. 2018 Jan 16;48(1):107-119.e4. doi:

10.1016/j.immuni.2017.12.007. Epub 2018 Jan 9. PMID: 29329948 or Burrack KS et al., Immunity. 2018 Apr 17;48(4):760-772.e4. doi: 10.1016/j.immuni.2018.03.012. Epub 2018 Apr 3. PMID: 29625893)

Antibodies used in western blot and IP- Most of the primary antibodies used for western blotting and immunoprecipitation in this study are widely used and well validated. The mentioned antibodies are tested by the manufacturer and/or in our lab:
anti-pIgR- staining of Molt4 whole cell lysate by western blot analysis
anti-IgA- staining of lysates from human tonsil, plasma and spleen by western blot analysis; Immunoprecipitation of IgA from human plasma and developed by western blot analysis
anti-phospho-Erk1/2- staining of extracts from A-431, HeLA, C6, COS-7 cell lines by western blot analysis
anti-Erk1/2- staining of extracts from NIH/3T3, PC12 and COS cells by western blot analysis
anti-DUSP5- staining of MEF (Mouse embryonic fibroblast cell line) whole cell lysate by western blot analysis
anti-β-actin- staining of extracts from ACTA1, ACTA2, ACTB, ACTC, ACTG1, ACTG2, HeLa cell lines by western blot analysis
anti-β-actin- staining of extracts from NIH/3T3, HeLa, COS, PC12 cell lines by western blot analysis
anti-IgA- secretory component staining of MCF7 and MDA-MB-361 whole cell lysates by western blot analysis

----
Antibodies used in multiplex immunohistochemistry- Most of the primary antibodies used for multiplex immunohistochemistry in this study are widely used and well validated. The mentioned antibodies are tested by immunohistochemical analysis by the manufacturer and/or in our core facility:

anti-CD3- staining of sections of human tonsil tissues by immunohistochemistry
anti-CD4- staining of sections of human tonsil and lymph node tissues by immunohistochemistry
anti-CD8- staining of sections of human tonsil and spleen tissues by immunohistochemistry
anti-CD19- staining of sections of human lymphoma, tonsil, precursor B-cell lymphoblastic leukemia/lymphoma, B-cell chronic lymphocytic leukemia/small lymphocytic lymphoma tissues by immunohistochemistry
anti-CD20- staining of sections of human tonsil, mantle cell lymphoma, B-cell chronic lymphocytic leukemia/small lymphocytic lymphoma tissues by immunohistochemistry
anti-CD138- staining of sections of human high-grade myeloma, tonsil, large B-cell lymphoma and appendix by immunohistochemistry
anti-pan-cytokeratin- staining of sections of human tonsil, seminoma, liver, merkel cell tumour tissues by immunohistochemistry
anti-pIgR- staining of sections of Cal27 xenograft tissue and HepG2 cells by immunohistochemistry
anti-IgA- staining of sections of human colon and tonsil tissues by Immunohistochemistry
anti-IgG- staining of sections of human tonsil tissues by Immunohistochemistry

Eukaryotic cell lines

Policy information about cell lines

Cell line source(s)	OVCAR3, A549, NIH-H23, HEK-293T, K562, THP1 cells were purchased from ATCC (Manassas, VA); OVCAR4, OVCAR5 and OVACR8 cells were procured from National Cancer Institute (Bethesda, MD); Kuramochi cell line was procured from JCRB Cell Bank, Japan. Human Ovarian Surface Epithelial (OSE) Cells were purchased from ScienCell Research Laboratories.
Authentication	Cell lines were not authenticated
Mycoplasma contamination	Cell lines were routinely tested negative for mycoplasma contamination
Commonly misidentified lines (See ICLAC register)	None

Animals and other organisms

Policy information about studies involving animals; ARRIVE guidelines recommended for reporting animal research

Laboratory animals	Female, 4-6 weeks old RAG1-deficient (B6.129S7-RAG1 knock-out) mice and NSG mice of same age groups were procured from Charles River Laboratories and Jackson Laboratory, respectively
Wild animals	None
Field-collected samples	None
Ethics oversight	Institutional Animal Care and Use Committee at the University of South Florida

Note that full information on the approval of the study protocol must also be provided in the manuscript.

Human research participants

Policy information about studies involving human research participants

Population characteristics	-Human high grade serous ovarian cancer (HGSOC) tissues were procured under protocols approved by the Committee for the Protection of Human Subjects at Dartmouth-Hitchcock Medical Center, and under a protocol approved by H. Lee Moffitt Cancer Center. All specimens were classified as surgical discard and remained totally de-identified. Stage III-IV human HGSOC

specimens and malignant ascites samples were procured. Use of samples in MCC cohort TMA was approved by institutional review board at Moffitt Cancer Center. Use of samples in the NHS cohort was approved by institutional review board at Brigham and Women's Hospital and Harvard T.H. Chan School of Public Health. Use of samples in NECC cohort TMA was approved by institutional review boards at Brigham and Women's Hospital and Dartmouth Medical School. Universal consent form was obtained for all subjects. All HGSOC specimens analyzed in this study are described in Supplementary Table 1.

- We obtained randomly selected peripheral blood mononuclear cells (PBMCs) from de-identified, cancer-free female donors (Moffitt Cancer Center).

Recruitment	None
Ethics oversight	Moffitt Cancer Center Institutional Review Board

Note that full information on the approval of the study protocol must also be provided in the manuscript.

Flow Cytometry

Plots

Confirm that:

☒ The axis labels state the marker and fluorochrome used (e.g. CD4-FITC).

☒ The axis scales are clearly visible. Include numbers along axes only for bottom left plot of group (a 'group' is an analysis of identical markers).

☒ All plots are contour plots with outliers or pseudocolor plots.

☒ A numerical value for number of cells or percentage (with statistics) is provided.

Methodology

Sample preparation	Flow cytometry was performed by staining with Zombie NIR (BioLegend) or Zombie Yellow (BioLegend) or DAPI (ThermoScientific) viability dye, blocking with anti-CD16/32 (BioLegend), and staining for 30 min at 4ºC with the following anti-human antibodies: CD45 (BD Biosciences, HI30, 1:300), CD3 (BD Biosciences, SK7, 1:200), CD19 (BD Biosciences, HIB19, 1:200), CD20 (BioLegend, 2H7, 1:200), CD38 (BD Biosciences, HIT2, 1:200), CD138 (BioLegend, MI15, 1:200), CD27 (BD Biosciences, M-T271, 1:200), IgA (Tonbo Biosciences, 35-8016-M001, 1:20), IgG (BioLegend, M1310G05, 1:200), IgM (BioLegend, MHM-88, 1:200), IgD (BD Biosciences, IA6-2, 1:100), IgE (BD Biosciences, G7-26, 1:100), EpCAM (BD Biosciences, KS1/4, 1:200), pIgR (ThermoScientific, PA5-35340, 1:50) or tetramers against TSPAN7 or BDNF. For intracellular staining for immunoglobulin isotypes, cells were first incubated with surface staining antibodies (30 min in ice), followed by fixation (30 min in RT) (eBioscience) and finally incubation with the antibodies in the permeabilization buffer (eBioscience) with antibodies for intracellular markers (45 min in RT).<br>Mice xenograft tumour single cell suspensions or splenocytes were blocked with Fc blocker (BioLegend) and analyzed by flow cytometry after incubation for 30 min at 4ºC with following anti-mouse antibodies: CD45 (BioLegend, 30-F11), CD11b (BioLegend, M1/70), CD351 (BioLegend, TX61) or with APC-conjugated human IgA. Splenocytes from RAG1-deficient mice were mechanically dissociated and RBCs were removed, followed by neutralization of Fcα/μ receptor (Fcα/μR) by incubation with CD351-neutralizing antibodies (BioLegend, TX61, 137303) or with isotype controls (BioLegend, 400123) at a concentration of 2.0 μg/106 cells in 100 μl volume for 30 min in ice. After washing, splenocytes were then incubated with APC-conjugated human IgA for another 30 min in ice and analyzed by flow cytometry.
Instrument	FACS LSR-II and FACS Aria sorter, BD Biosciences
Software	FACS Diva and FlowJo_V10
Cell population abundance	Post-sort purity was analysed for samples with more than 5000 target cells collected. In all cases purity was greater than 95%
Gating strategy	Gating strategy is shown in Extended Data Figures 26-29

☒ Tick this box to confirm that a figure exemplifying the gating strategy is provided in the Supplementary Information.
